# HIV Vaccine Design to Target Germline Precursors of Glycan-Dependent Broadly Neutralizing Antibodies

**DOI:** 10.1016/j.immuni.2016.08.016

**Published:** 2016-09-20

**Authors:** Jon M. Steichen, Daniel W. Kulp, Talar Tokatlian, Amelia Escolano, Pia Dosenovic, Robyn L. Stanfield, Laura E. McCoy, Gabriel Ozorowski, Xiaozhen Hu, Oleksandr Kalyuzhniy, Bryan Briney, Torben Schiffner, Fernando Garces, Natalia T. Freund, Alexander D. Gitlin, Sergey Menis, Erik Georgeson, Michael Kubitz, Yumiko Adachi, Meaghan Jones, Andrew A. Mutafyan, Dong Soo Yun, Christian T. Mayer, Andrew B. Ward, Dennis R. Burton, Ian A. Wilson, Darrell J. Irvine, Michel C. Nussenzweig, William R. Schief

**Affiliations:** 1 Department of Immunology and Microbial Science, The Scripps Research Institute, La Jolla, CA 92037, USA; 2 IAVI Neutralizing Antibody Center, The Scripps Research Institute, La Jolla, CA 92037, USA; 3 Center for HIV/AIDS Vaccine Immunology and Immunogen Discovery, The Scripps Research Institute, La Jolla, CA 92037, USA; 4 Koch Institute for Integrative Cancer Research, MIT, Cambridge, MA 02139, USA; 5 Laboratory of Molecular Immunology, The Rockefeller University, New York, NY 10065, USA; 6 Department of Integrative Structural and Computational Biology, The Scripps Research Institute, La Jolla, California, USA; 7 The Ragon Institute of Massachusetts General Hospital, Massachusetts Institute of Technology and Harvard University, Cambridge, MA 02139, USA; 8 Skaggs Institute for Chemical Biology, The Scripps Research Institute, La Jolla, CA 92037, USA; 9 Howard Hughes Medical Institute, Chevy Chase, MD 20815, USA; 10 Departments of Biological Engineering and Materials Science & Engineering, MIT, Cambridge, MA 02139, USA

## Abstract

Broadly neutralizing antibodies (bnAbs) against the N332 supersite of the HIV envelope (Env) trimer are the most common bnAbs induced during infection, making them promising leads for vaccine design. Wild-type Env glycoproteins lack detectable affinity for supersite-bnAb germline precursors and are therefore unsuitable immunogens to prime supersite-bnAb responses. We employed mammalian cell surface display to design stabilized Env trimers with affinity for germline-reverted precursors of PGT121-class supersite bnAbs. The trimers maintained native-like antigenicity and structure, activated PGT121 inferred-germline B cells ex vivo when multimerized on liposomes, and primed PGT121-like responses in PGT121 inferred-germline knockin mice. Design intermediates have levels of epitope modification between wild-type and germline-targeting trimers; their mutation gradient suggests sequential immunization to induce bnAbs, in which the germline-targeting prime is followed by progressively less-mutated design intermediates and, lastly, with native trimers. The vaccine design strategies described could be utilized to target other epitopes on HIV or other pathogens.

## Highlights

• Developed mammalian cell display to design germline-targeting native-like trimers
• Germline-targeting trimers retain native-like antigenicity and structure
• Germline-targeting trimers prime glycan-dependent HIV bnAb responses in knockin mice
• Designed boosting schemes intended to induce bnAbs

## Teaser

Elicitation of broadly neutralizing antibodies (bnAbs) is a critical HIV vaccine goal. Steichen et al. have developed immunogens that prime germline-precursor B cells for the bnAb PGT121 and can therefore initiate bnAb induction. The authors have also designed boosting immunogens to shepherd the antibody maturation to develop bnAbs.

## Introduction

A vaccine is needed for global HIV prevention. Broadly neutralizing antibodies (bnAbs) directed against relatively conserved epitopes in the otherwise highly antigenically variable HIV envelope (Env) glycoprotein trimer offer important guides for vaccine design. BnAbs have been isolated from a small minority of HIV-infected individuals and have been shown to protect against challenge in various animal models, but have not been induced by vaccination in humans or standard animal models (Burton and Hangartner, 2016, Mascola and Haynes, 2013, West et al., 2014). BnAbs recovered from natural infection are typically highly mutated (Klein et al., 2013a, Mouquet et al., 2010, Pancera et al., 2010, Scheid et al., 2009, Walker et al., 2011, Xiao et al., 2009, Zhou et al., 2010) and many also contain insertions and/or deletions (Kepler et al., 2014), owing to chronic stimulation of B cells by mutating Env. Many bnAbs also possess unusually long or short heavy-chain complementarity determining region 3 (CDR3) loops (Scheid et al., 2011, Walker et al., 2009, Walker et al., 2011, Wu et al., 2011, Zhou et al., 2010) and some are polyreactive (Haynes et al., 2005). Less mutated bnAbs with fewer unusual features have been engineered, offering more tractable goals for consistent vaccine elicitation (Georgiev et al., 2014, Jardine et al., 2016b, Sok et al., 2013). Overall, bnAb elicitation by vaccination presents a major challenge.

Recombinant native-like trimers are promising HIV vaccine components because they contain the conformational epitopes of most known bnAbs and lack many non-neutralizing epitopes present on less native constructs (Julien et al., 2013, Kong et al., 2016, Kwon et al., 2015, Lyumkis et al., 2013, Pancera et al., 2014, Sanders et al., 2013, Scharf et al., 2015). However, native-like trimers have features that might impede bnAb induction; they are highly glycosylated and expose both strain-specific neutralizing epitopes and non-neutralizing epitopes. Immunization with native-like trimers in standard mouse, rabbit, and macaque models has thus far elicited either non-neutralizing antibodies (Hu et al., 2015) or neutralizing antibodies only against the immunogen strain (de Taeye et al., 2016, Sanders et al., 2015) analogous to the strain-specific responses to the seasonal flu vaccine in humans. Induction of HIV bnAbs will likely require development of vaccination strategies that focus responses to relatively conserved, sub-dominant epitopes and avoid or suppress responses to non-neutralizing and strain-specific epitopes.

Germline targeting, a vaccine priming strategy to initiate the affinity maturation of specific germline-precursor B cells, could help solve this immunofocusing problem by preferentially activating bnAb precursors (Dimitrov, 2010, Xiao et al., 2009). The strategy aims to activate bnAb-precursor B cells, select productive (bnAb-like) somatic mutations, and produce memory B cells that can be boosted subsequently to select additional productive mutations (Dosenovic et al., 2015, Jardine et al., 2015). For some bnAbs, inferred precursors have affinity for Env from particular HIV isolates (Andrabi et al., 2015, Doria-Rose et al., 2014, Gorman et al., 2016, Liao et al., 2013), facilitating design of priming immunogens based on Env from those isolates (Haynes et al., 2012). For other bnAbs, efforts to identify wild-type (WT) Env that bind inferred precursors have failed (Hoot et al., 2013, Jardine et al., 2013, McGuire et al., 2013, Scheid et al., 2011, Xiao et al., 2009, Zhou et al., 2010). These latter cases require design of modified Env to serve as a priming immunogen (Dimitrov, 2010, Pancera et al., 2010, Xiao et al., 2009, Zhou et al., 2010). Proof of principle that designed germline-targeting immunogens can activate their intended precursors and generate a potentially boostable memory response was recently demonstrated in knockin mice with B cell precursors for VRC01-class bnAbs directed to the CD4-binding site (Dosenovic et al., 2015, Jardine et al., 2015, McGuire et al., 2016). After a germline-targeting prime, induction of bnAbs is expected to require a succession of boosts, driving a succession of germinal-center reactions, in order to select sufficient mutations (Dimitrov, 2010, Dosenovic et al., 2015, Haynes et al., 2012, Jardine et al., 2013, Jardine et al., 2015, Jardine et al., 2016b, Klein et al., 2013b, Liao et al., 2013, McGuire et al., 2013, McGuire et al., 2016, Pancera et al., 2010, Wu et al., 2011, Xiao et al., 2009, Zhou et al., 2010). Supporting the concept that sequential immunization with different immunogens will be required to develop a bnAb response, native-like trimers but not germline-targeting immunogens were found to boost near-bnAb B cells (bearing a mature VRC01-class bnAb heavy chain) to induce cross-neutralizing Abs (Dosenovic et al., 2015).

Glycan-dependent bnAbs in general, and N332-supersite bnAbs in particular, are important targets for germline-targeting vaccine design. In a recent longitudinal study of HIV infection in Africa, more than half of the HIV-infected individuals who produced bnAb responses produced them against glycan-directed epitopes, the majority of which were within the N332 supersite (Landais et al., 2016). The prevalence of N332-supersite bnAb responses is probably due in part to the high accessibility of their epitopes on top of the trimer.

Among N332-supersite bnAbs, PGT121-class bnAbs have been particularly well characterized, providing strong rationale for germline-targeting efforts. PGT121-class bnAbs are among the most potent bnAbs (Mouquet et al., 2012, Walker et al., 2011), and PGT121 delivered passively to macaques protects against SHIV (simian-human immunodeficiency virus) infection (Moldt et al., 2012, Shingai et al., 2014) and can suppress viremia when delivered after infection (Barouch et al., 2013, Shingai et al., 2013). However, PGT121-class inferred precursors show no measureable affinity for WT Env proteins that have been evaluated (Mouquet et al., 2012, Sok et al., 2013). Thus, development of a priming immunogen for PGT121-class precursors requires either design of a modified Env or identification of a natural Env with PGT121-class germline-binding capacity. Crystal structures have been determined for several PGT121-class bnAbs in complex with either BG505 SOSIP native-like trimers or gp120 (Garces et al., 2015, Garces et al., 2014, Julien et al., 2013, Kong et al., 2016, Pancera et al., 2014) and for unliganded structures of two germline-reverted PGT121 variants (Mouquet et al., 2012, Sok et al., 2013), providing critical information to guide design of modified Env for PGT121-class germline targeting.

PGT121-class bnAbs interact with conformationally flexible structures on HIV Env, including several glycans and the V1 variable loop, making computational design of germline-targeting Env challenging. Here, we developed a structure-guided directed evolution approach, by using mammalian cell surface display, to design PGT121-class germline-targeting stabilized-trimer immunogens. We multimerized these trimers on liposomes and evaluated trimer and liposome immunogens via biophysical, structural, and ex vivo B cell activation analyses. We further evaluated germline-targeting trimers by vaccination in PGT121 inferred-germline knockin mice. Our design process produced design intermediates with increasing levels of epitope modification between WT and germline-targeting trimers. These results led to our hypothesizing prime-boosting strategies in which a germline-targeting prime is followed by boosts with progressively less modified design intermediates and then with WT Env, followed ultimately by a cocktail of Env variants to expand breadth. Evaluation of several of these prime-boosting strategies in PGT121 germline and chimeric knockin mice is described in a related study (Escolano et al., 2016).

## Results

### Design of Germline-Targeting gp120s

**Figure 1:**
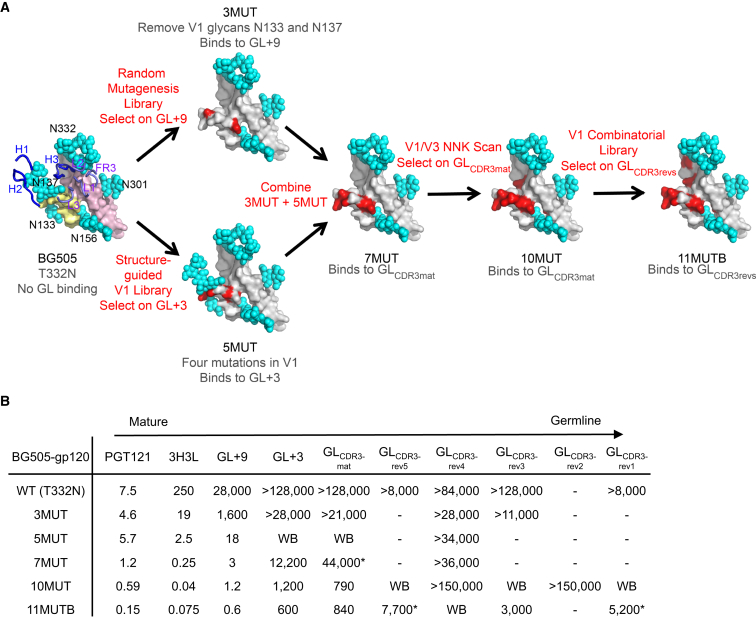
Mammalian-Display Directed-Evolution Design Pathway for PGT121 Germline-Targeting Env-Based Immunogens (A) Models of the PGT121 epitope are shown for each immunogen, with positions of germline-targeting mutations colored red and glycans depicted with cyan spheres. The epitope of BG505 is colored yellow (variable loop 1) and pink (variable loop 3). The paratope of PGT122 is mapped onto the epitope of BG505 and shown in tube representation (heavy chain, blue; light chain, purple). (B) Binding K_D_s of mature, intermediately mutated, and germline-reverted variants of PGT121 for BG505 gp120 and germline-targeting gp120s, determined by SPR. SPR K_D_s are the average of two or three experiments. Asterisk, complex binding kinetics; WB, weak binding; -, not done.

We identified mammalian cell surface display as a desirable platform for engineering modified HIV Env constructs with affinity for inferred-germline PGT121 Abs because it should allow for optimization of monomeric or multimeric antigens bearing mammalian glycans (Chen et al., 2008). Therefore, we developed a lentivirus-based mammalian-cell-surface-display method to carry out directed evolution of HIV gp120 monomers and gp140 trimers (Figure S1). Structural analysis of the PGT121 interaction with gp120 (Julien et al., 2013, Pancera et al., 2014) led us to hypothesize that the V1 and V3 loops were the key sites for germline-targeting mutations. For selection agents, we assembled a collection of six germline-reverted Abs, all using heavy-chain genes VH4-59, D3-3, and J6 and light-chain genes V3-21 and J3, with varying degrees of mutation in the D gene and L-CDR3 and with differences in the non-templated regions at the V-D and D-J boundaries (Figure S2). Ranked by similarity to the germline D and L-CDR3 sequences, these Abs are GL_CDR3rev1_ (most similar to germline D and L-CDR3), GL_CDR3rev2_, GL_CDR3rev3_, GL_CDR3rev4_, GL_CDR3rev5_, and GL_CDR3mat_ (germline V and J genes but with mature CDR3 loops). We began by screening libraries based on the template genes BG505 T332N gp120 and BG505 SOSIP T332N gp140 (Sanders et al., 2013), in which the conserved glycosylation site at position 332 absent in BG505 was introduced (Figure 1A). These molecules had no detectable affinity for germline-reverted PGT121 Abs (Figure 1B and Table S1). Therefore, we employed a “bootstrapping” approach: for initial screening we utilized two variants of GL_CDR3mat_, one with nine PGT121 light-chain mutations (GL+9, with 28 μM affinity for BG505 T332N gp120) and another with three light-chain mutations (GL+3, no detectable affinity for BG505 T332N gp120) (Figure S2). Screening a gp120 random mutagenesis library for binding to GL+9 led to the molecule 3MUT, with mutations T135A and T139I, which eliminate the V1 loop glycosylation sites at positions 133 and 137 (Figure 1A). Screening a gp140 structure-guided V1 loop library for binding to GL+3 led to the isolation of 5MUT, with four different mutations (V134Y, N136P, I138L, and D140N) in the V1 loop. Combining the mutations in 3MUT and 5MUT produced 7MUT gp120, our first construct with quantifiable affinity for GL_CDR3mat_ (*K*_D_ = 44 μM, Figures 1A and 1B). To improve this affinity further, we screened a gp120 V1- and V3-loop saturation mutagenesis library for binding to GL_CDR3mat_; combining the most enriched mutations (N137F, T320F, and Q328M) with 7MUT produced 10MUT, with *K*_D_ ≈1 μM for GL_CDR3mat_ (Figures 1A and 1B). Finally, to increase affinity and breadth, we screened a gp120 V1 loop directed mutagenesis library for binding to GL_CDR3rev2_ and GL_CDR3rev4_ (Figure S2). This approach culminated in 11MUT_B_, with *K*_D_s of ∼5 μM, ∼3 μM, and ∼8 μM for GL_CDR3rev1_, GL_CDR3rev3_, and GL_CDR3rev5_, respectively, and detectable but not quantifiable binding to GL_CDR3rev4_ (Figure 1A, Figure S3, and Table S1). Thus, mammalian display-directed evolution enabled the design of germline-targeting gp120 molecules with appreciable affinity for PGT121 germline-reverted antibodies.

### Design of Stabilized and Germline-Targeting Trimers

**Figure 2:**
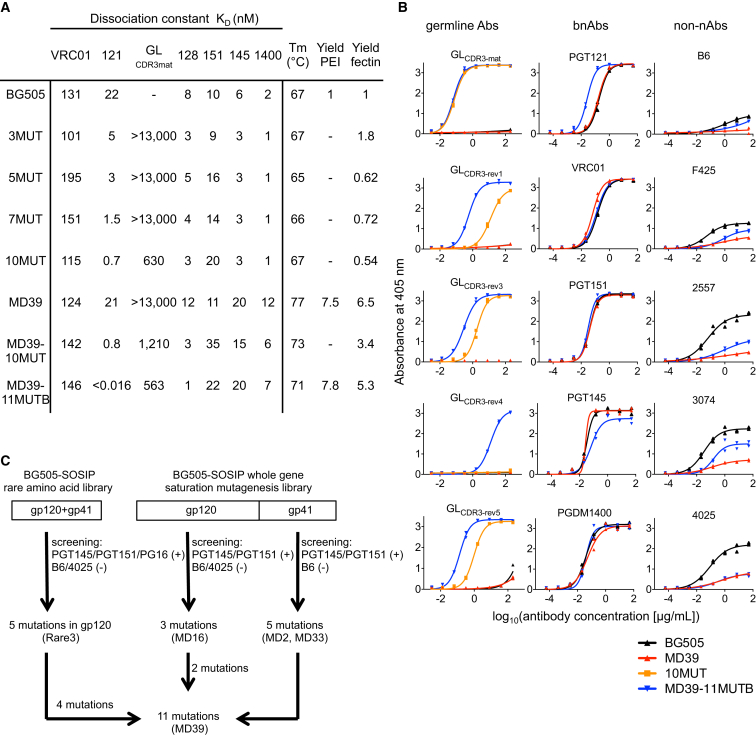
Design of Mutations to Stabilize BG505-SOSIP and Germline-Targeting Native-Like Trimers (A) Biophysical properties of stabilized BG505-SOSIP and germline-targeting trimers. Antigenic profile was assessed by SPR, thermostability measurements were made by DSC, and expression was determined as yield of purified protein relative to BG505-SOSIP made with PEI or 293Fectin transfection reagents in 293F cells. Monovalent *K*_D_s were measured by SPR with trimer ligand and Fab analyte except for PGT145 and PGDM1400, for which monovalent *K*_D_s were determined with IgG ligand and trimer analyte. For PGT151, a one-to-one binding model gave a relatively poor kinetic fit. (B) Antigenic profile of stabilized BG505-SOSIP and germline-targeting trimers by ELISA. Data are representative of two independent experiments, each done in duplicate. (C) Mammalian display-directed evolution design pathways for engineering stabilized native-like trimers.

For initial design of germline-targeting and boosting trimers, we transferred the germline-targeting mutations from the gp120 versions of 3MUT, 5MUT, 7MUT and 10MUT onto the BG505 SOSIP trimer platform. These molecules displayed characteristics of native-like trimers, such as high affinity for the trimer-specific bnAb PGT151 (Falkowska et al., 2014) and a melting temperature (*T*_m_) similar to that of BG505 SOSIP (Figure 2A). Furthermore, all had similar monovalent affinities for PGT121 and GL_CDR3mat_ as their gp120 counterparts (Figure 2A), indicating that the germline-targeting mutations were transferable to a native-like trimer.

In addition to binding bnAb putative precursors, germline-targeting trimers should have an otherwise native-like antigenic profile, with high affinity for bnAbs and no significant affinity for non-neutralizing antibodies directed to epitopes exposed on monomeric gp120 but buried or conformationally absent on the trimer. BG505 SOSIP gp140, the trimer on which our PGT121-class germline-targeting designs were based, displays undesirable binding to V3 non-neutralizing antibodies (Sanders et al., 2013) (Figure 2B and Figure S4) and induces non-neutralizing V3 responses in mice, rabbits, and macaques (de Taeye et al., 2016, Hu et al., 2015, Sanders et al., 2015), indicating that this trimer samples conformational states that expose non-neutralizing epitopes. Furthermore, BG505 SOSIP gp140 displayed on mammalian cells via a PDGFR linker showed strong binding to trimer-structure-dependent bnAbs (PGT151 and PGT145) (Falkowska et al., 2014, Walker et al., 2011) but also to non-neutralizing antibodies directed to the V3 loop (4025) (Gorny et al., 2011) and the CD4-binding site (b6) (Barbas et al., 1992) (not shown), suggesting the coexistence on the cell surface of native-like trimers along with non-native trimers, dimers, and/or monomers. We also found that adding germline-targeting mutations to BG505 SOSIP reduced the already modest expression by 50% (Figure 2A). Therefore, we sought to use mammalian-display directed evolution to improve the antigenic profile, thermal stability, and expression of the BG505 SOSIP trimer and germline-targeting trimers.

Our trimer improvement effort focused on two types of libraries: (1) whole-gene saturation mutagenesis libraries and (2) a combinatorial library sampling the one or two most common HIV residues at Env positions where BG505 uses rare (frequency < 10%) HIV residues (Figure 2C). The rare library, which allowed variation at eleven positions in gp120 and two in gp41, was screened for binding to trimer-structure-preferring bnAbs PGT145, PGT151, and PG16 and for lack of binding to non-neutralizing antibodies b6 and 4025. This produced the Rare3 clone with five mutations in gp120 (T106E, M271I, F288L, T290A, N363Q) and with the expression yield improved by a factor of ∼2 and the *T*_m_ increased by 1.4°C (Figure S5). The saturation mutagenesis library was constructed in three segments, two covering gp120 and one for gp41 (Figure 2C). Next-generation sequencing and bioinformatics were employed to analyze the results of the first two sorts (Jardine et al., 2016a), and Sanger sequencing was used to identify enriched clones that survived four or five sorts. Enriched mutations from both sequencing methods were combined and tested in soluble trimers and were also assembled into combinatorial libraries and re-screened with the same antibodies as before. The gp41 library produced MD2, with an L568D point mutation that increased expression levels by a factor of ∼4, and MD33, with four additional mutations (F519S, A561P, V570H, and R585H) that increased the *T*_m_ by 4°C and improved expression by a factor of ∼7, relative to BG505 SOSIP (Figure S5). The gp120 library produced MD16, with three mutations (F223W, R304V, and A319Y) and reduced binding to V3 non-neutralizing antibodies (Figure S4). Finally, mutations from Rare3, MD16, and MD33 were combined to produce MD39 with 11 mutations (F223W and T290A did not improve the biophysical properties of the trimer and were excluded; data not shown). Compared to BG505 SOSIP.D664, the MD39 yield improved by a factor of ∼7, *T*_m_ increased by 10°C, and antigenic profile improved, with reduced V3 Ab reactivity and similar bnAb binding except for slightly reduced affinities for V2 apex bnAbs (Figures 2A and 2B and Figure S5).

Combining the MD39 mutations with germline-targeting mutations produced germline-targeting trimers with improved properties. MD39-10MUT had a 6-fold-improved yield and 6°C higher *T*_m_ as compared to 10MUT (Figure 2A and Figure S5). Our most advanced germline-targeting trimer, MD39-11MUT_B_, the only trimer with detectable affinity for five of six PGT121 germline reverted variants tested (Figure 2B), had excellent yield, thermal stability, and antigenic profile (Figures 2A and 2B). Directed evolution therefore produced native-like trimers with improved potential functionality via both stabilization and germline-targeting mutations.

### Structural Analysis

**Figure 3:**
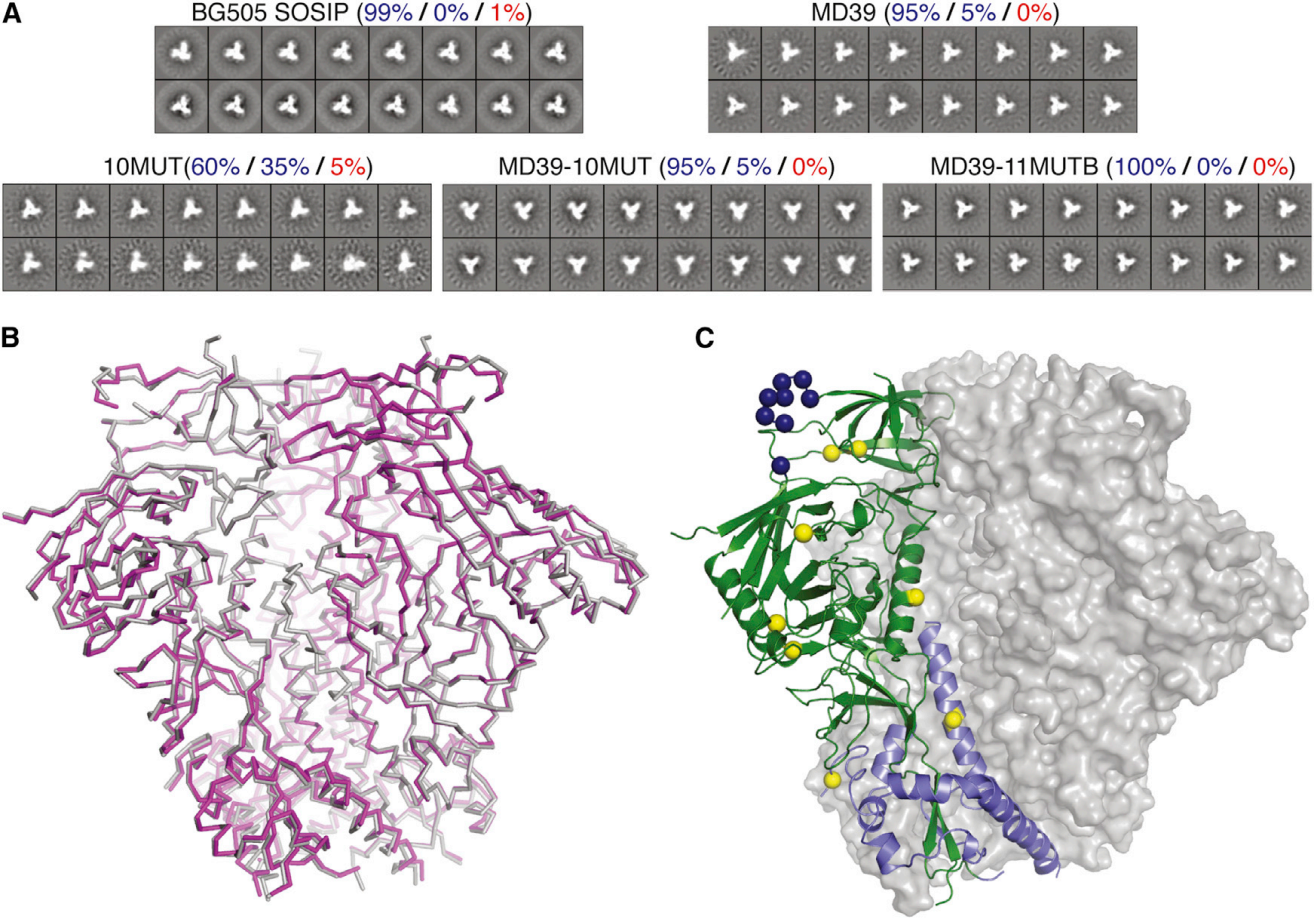
Structural Analysis of Stabilized Germline-Targeting Trimers (A) Negative-stain EM analysis of the indicated trimers, in which the 2D class averages are shown and classified as percent closed native-like (blue), partially open native-like (blue), or non-native (red) (Pugach et al., 2015). We observed ± 5% variation between experiments. (B) The crystal structure of a stabilized germline-targeting trimer (MD39-10MUT_A_, in purple) is shown aligned to BG505-SOSIP (PDB: 5CEZ), in gray. (C) Crystal structure of MD39-10MUT_A_ (one subunit of gp41 is shown as a purple cartoon and one subunit of gp120 is shown as a green cartoon) highlighting the MD39 stabilizing mutations in yellow spheres and germline-targeting mutations in blue spheres.

To ascertain whether stabilized, PGT121 germline-targeting trimers maintain native-like structure, we conducted crystallography and electron microscopy (EM) studies. Negative-stain EM two-dimensional (2D) classification revealed that all four trimers tested (MD39, 10MUT, MD39-10MUT, and MD39-11MUT_B_) were characterized by a high fraction (≥95%) of native-like structural features and were similar in appearance to BG505 SOSIP (Figure 3A). The MD39 mutations improved the structural uniformity of the 10MUT trimer; the amount of flexible, native open conformations dropped from 35% to 5% between 10MUT and MD39-10MUT (see the Experimental Procedures for a description of the 2D classification system). Our best germline-targeting trimer, MD39-11MUT_B_, exhibited 100% native closed conformations and was indistinguishable from BG505 SOSIP by EM. For higher resolution analysis, we solved a 4.5 Å resolution crystal structure of MD39-10MUT_A_, a variant of MD39-10MUT with one mutation added and another removed (see Supplemental Experimental Procedures), complexed with 35O22 and PGT124 (Garces et al., 2014, Sok et al., 2013). Although this resolution precluded analyses of side-chain conformations, and the interface between trimer and PGT124 could not be analyzed due to missing V1 loop density, the structure accurately determined the backbone positions for most (1,659 of 1,692) residues of gp140. Superposition of the gp140 backbones in this structure and in the 3.0 Å structure of BG505 SOSIP N137A complexed with 3H109L and 35O22 (PDB: 5CEZ) or the 3.1 Å structure of BG505 SOSIP bound to PGT122 and 35O22 (PDB: 4TVP) gives backbone root-mean-square deviation values of 0.7 and 1.1 Å, respectively (Figure 3B). We conclude that MD39-10MUT_A_, with 20 mutations relative to BG505 SOSIP T332N (Figure 3C), retains an overall native-like conformation.

### Liposome Platform

**Figure 4:**
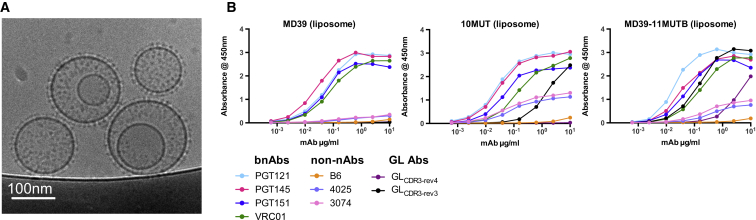
Structure and Antigenicity of Trimer Liposomes (A) Cryo-EM image of MD39-11MUT_B_ trimer liposomes. (B) ELISA analysis of trimer liposomes for MD39, 10MUT, and MD39-11MUT_B_. Data are representative of two experiments.

We have previously found that highly multimeric particulate immunogens are superior to trimeric immunogens for B cell activation ex vivo (Jardine et al., 2013) and for generation of antigen-specific memory B cells after immunization in vivo (Jardine et al., 2015). Therefore, we developed trimer-conjugated liposomes to improve the immunogenic potential of our germline-targeting trimers. Trimers with a C-terminal His-tag were attached to 145-nm mean diameter uni-lamellar liposomes (DSPC [1,2-distearoyl-sn-glycero-3-phosphocholine], 66.5%; cholesterol, 28.5%; DGS-NTA(Ni), 5%) via the histidine-Ni-NTA interaction. On average, 522 ± 92 trimers were attached to each liposome. Cryo-EM examination of trimer-decorated liposomes confirmed the dense particulate array of trimers (Figure 4A). ELISA analysis on intact vesicles indicated that trimer-decorated liposomes maintained the native-like antigenic profile and germline-binding properties of the soluble trimers (Figure 4B).

### Ex Vivo B Cell Activation

**Figure 5:**
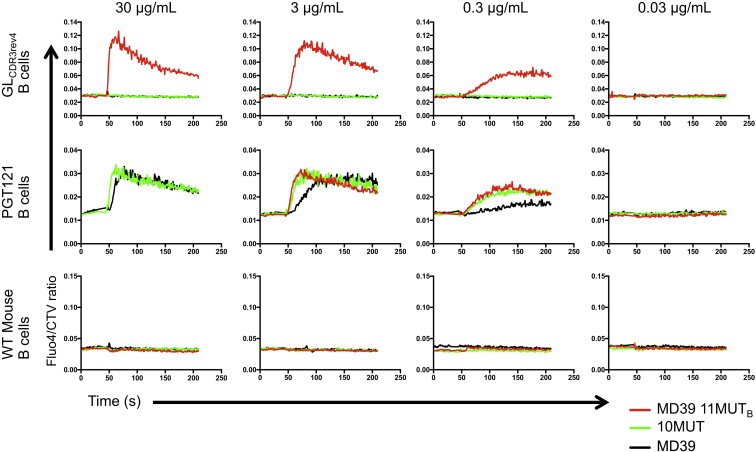
Ex Vivo B Cell Activation Assay Ca^2+^ flux transients detected as increases in Fluo-4 fluorescence after addition of trimer liposomes (MD39, 10MUT, MD39-11MUT_B_) at the indicated gp140 concentrations. Data are shown for germline-reverted PGT121 (GL_CDR3rev4_) B cells (top), mature PGT121 B cells (middle), and WT mouse B cells (bottom). Data are representative of two experiments.

To determine whether germline-targeting trimers or trimer liposomes can specifically activate germline or mature PGT121 B cells, we conducted ex vivo experiments with B cells harvested from PGT121 GL_CDR3rev4_ knockin mice, PGT121 bnAb knockin mice, and wild-type (WT) mice (Escolano et al., 2016) (PGT121 GL_CDR3rev4_ is referred to as GL_HL_121 in Escolano et al.). B cell activation was measured by a Ca^2+^-flux assay (Ota et al., 2012) for MD39, 10MUT, and MD39-11MUT_B_ (as trimers and trimer liposomes) and was compared to positive control activators (ionomycin and IgM) and negative control activators (ovalbumin) (Figure 5). Soluble trimers did not specifically activate germline PGT121 B cells (Figure S6) but did activate PGT121 bnAb B cells in a dose-dependent manner (Figure S6), with the strongest activation by MD39-11MUT_B_, in accordance with its higher affinity for PGT121 as compared to MD39 and 10MUT (Figure 2C). MD39-11MUT_B_ liposomes activated PGT121 germline B cells at concentrations as low as 0.3 μg/mL, whereas MD39 or 10MUT liposomes failed to activate at concentrations up to 300 μg/mL (Figure 5 and Figure S6). We conclude that MD39-11MUT_B_ liposomes have promise as a PGT121-class germline-targeting prime.

### Priming Germline PGT121 Responses in Knockin Mice

**Figure 6:**
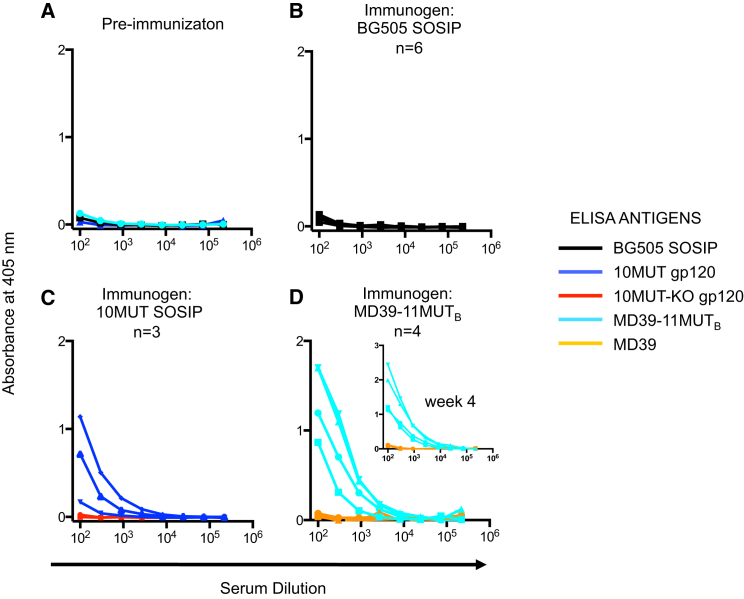
Serum ELISA Binding Specificity of PGT121 Germline-Reverted (GL_CDR3rev4_) Knockin Mice Before and After a Single Immunization with BG505 SOSIP, 10MUT SOSIP, or MD39-11MUT_B_ SOSIP (A) Pre-immunization sera showed no reactivity to the four antigens tested (10MUT gp120, 10MUT-KO gp120, MD39, and MD39-11MUT_B_). (B) Mouse sera from 2 weeks after immunization with BG505 SOSIP showed no reactivity to BG505 SOSIP. (C) Mouse sera from 2 weeks after immunization with 10MUT SOSIP showed reactivity to 10MUT gp120 and not to 10MUT-KO gp120. (D) Mouse sera from 2 weeks after immunization with MD39-11MUT_B_ SOSIP showed reactivity to the immunogen and not to MD39 SOSIP; mouse sera from 4 weeks after immunization showed similar results (inset). The number of mice used for each experiment is indicated. A duplicate experiment for (C) with two additional mice gave similar results.

To determine whether germline-targeting trimers can activate germline PGT121 B cells in vivo, we conducted priming immunizations in PGT121-GL_CDR3rev4_ knockin mice (Escolano et al., 2016). A combined five mice were immunized with 10MUT SOSIP, in two separate experiments, and four mice were immunized with MD39-11MUT_B_ SOSIP. In a control experiment, six mice were immunized with BG505 SOSIP. Two weeks after a single immunization, the sera was analyzed for immunogen- and epitope-specific responses. Sera of BG505-SOSIP-immunized mice showed no detectable binding to the BG505 SOSIP immunogen (Figure 6). In contrast, 4/5 10MUT-immunized mice and 4/4 11MUT_B_-immunized mice showed epitope-specific serum responses to either 10MUT-gp120 or MD39-11MUT_B_ SOSIP (Figure 6). We conclude that germline-targeting mutations, such as those in 10MUT and 11MUT_B_, are required for activation of inferred PGT121 germline B cells in vivo by BG505-based native-like trimers.

### Sequential Boosting Strategies

**Figure 7:**
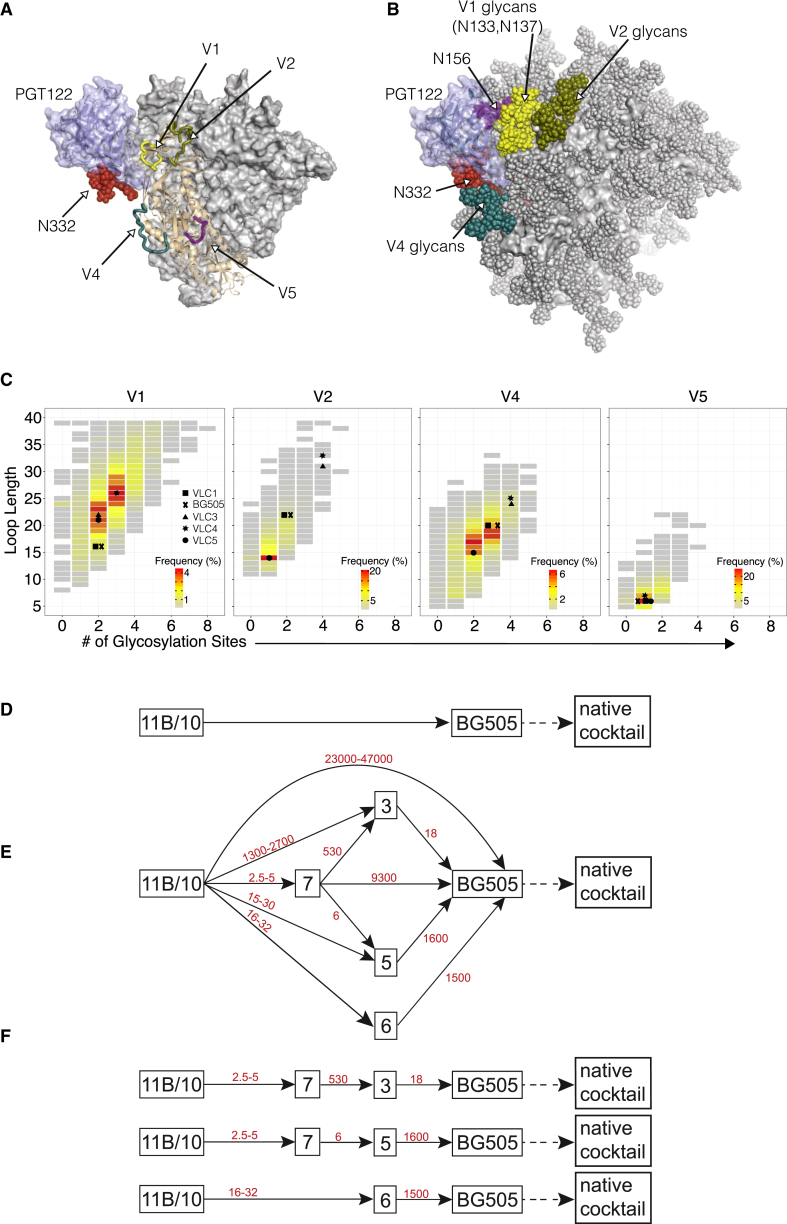
Sequential Boosting Schemes Employing a Native-Like Trimer Cocktail and Germline-Targeting Design Intermediates (A) Side view of a single PGT122 Fab (light blue cartoon and semi-transparent surface) bound to the BG505 SOSIP native-like gp140 trimer, based on PDB: 4NCO. The PGT122-bound gp140 subunit is shown in wheat-colored cartoon; the V1, V2, V4, and V5 variable loops on that subunit, modeled wherever missing in the crystal structure, are shown in yellow (V1), olive (V2), teal (V4), and magenta (V5); the N332 glycan is shown as red spheres; and the two other gp140 subunits are shown as gray surfaces. (B) Same model as in (A), except that glycosylation sites on the trimer have been decorated with Man_8_GlcNAc_2_ glycans shown as spheres (V1 glycans, red; V2 glycans, olive; V4 glycans, teal; N156 glycan, magenta; N332 glycan, red; all other glycans, gray), and all trimer subunits are shown as gray surfaces. (C) 2D histogram of variable loop (V1, V2, V4, and V5) length and number of glycosylation sites among 3,897 unique HIV Env sequences isolated from infected individuals and obtained from http://www.hiv.lanl.govcontent/index. Frequency is indicated by the color scale shown for each loop. The length and number of glycosylation sites for each loop of the native-like VLC trimers are indicated. Further elaboration of this cocktail could include accounting for sequence variation at non-variable-loop positions within the N332-epitope region (Figure S7C). (D) Basic scheme in which a germline-targeting prime (10MUT or 11MUT_B_) is boosted by a native-like trimer (BG505) and then by a cocktail of native-like trimers. (E) Diagram illustrating seven boosting schemes employing germline-targeting design intermediates (7MUT, 6MUT, 5MUT, and 3MUT) as boosts after a germline-targeting prime and before a native-like trimer; the scheme in (A) is included for reference. Relative affinity drops (in fold affinity decrease) for each boost, computed from Figure 1B as described in the text, are indicated as red numbers. (F) Linear diagrams of three of the best boosting schemes as ranked by favoring those with the smallest maximum affinity drop.

As noted above, induction of bnAbs after a germline-targeting prime is expected to require sequential boosting with epitope variants to mature the response. With PGT121-class germline-targeting candidates (10MUT and 11MUT_B_) in hand, we developed boosting strategies aiming to select PGT121-like mutations and induce bnAbs. We hypothesized that any sequential immunization strategy starting with a germline-targeting trimer should end with a native-like trimer, such as BG505 MD39 SOSIP, so as to select mutations productive for high-affinity interaction with native trimers present on circulating HIV strains. However, in order for PGT121-class antibodies to engage their epitope including the N137 glycan on the V1 loop, such antibodies must accommodate V1 loops diverse not only in sequence but also in length and number of glycosylation sites (Figures 7A–7C), implying that boosting with a single native-like trimer bearing a single V1 loop might not be sufficient. Indeed, boosting only with a BG505 native-like trimer would present a V1 loop that is significantly shorter than most (Figure 7B). Furthermore, modeling of variable loops and glycan conformations (not shown) suggested that diversity in the V2 and V4 loops might potentially impact the PGT121 epitope by altering conformational sampling of the V1 loop or N332 glycan, respectively (Figures 7A–7C), and immunodominant responses involving V2 or V4 could potentially sterically interfere with PGT121-class boosting. On the basis of these considerations, we hypothesized that a cocktail of native-like trimers displaying diverse variants of the V1, V2, and V4 loops, especially variants within hotspots of more frequently occurring combinations of length and number of glycosylation sites (Figure 7B), might be needed to select PGT121-class mutations favoring neutralization breadth. We therefore designed and produced four native-like trimers based on BG505 MD39 SOSIP and containing diverse loops for V1, V2, V4, and V5 (Figure 7C, Figures S7A and S7B, and Supplemental Experimental Procedures). These trimers, together with BG505 MD39 SOSIP, form a five-member variable loop cocktail (VLC) that might broaden PGT121-like responses initiated by a germline-targeting trimer (Figure 7D).

We then considered the question of what intermediate boosts, if any, might be employed between a germline-targeting prime and a native-like trimer. Our germline-targeting design intermediates become increasingly more native-like in the PGT121 epitope (e.g., 7MUT, 5MUT, and 3MUT have six, four, and two epitope mutations, respectively), but the 5MUT and 3MUT mutations are mutually exclusive (3MUT lacks two V1 glycans while 5MUT has those glycans but has four other V1 loop mutations) (Figure 1A). These considerations impose directionality on any boosting scheme (e.g., 7MUT should not be used after 5MUT or 3MUT or WT and 3MUT should not be used after 5MUT), thus limiting the number of possible schemes (Table S3). Considering only the most efficient directional schemes, those employing boosting pairs that differ by more than one mutation or involve substantial affinity changes (Table S4), we identified a total of seven potential boosting schemes (Figures 7E).

We sought to rank these schemes to allow prioritization for experimental testing. We reasoned that the least mutated antibody that shows measurable affinity for all of the potential boosting immunogens, GL+9, could serve as a proxy for intermediate PGT121-class antibodies developing after a germline-targeting prime and before a native-like boost. We further reasoned that the affinity drop, the ratio of GL+9 affinities for two immunogens, could be used to estimate the likelihood of successfully boosting memory B cells when the two immunogens are used in sequence (e.g., the GL+9 K_D_s for 7MUT and 3MUT are 3 nM and 1,600 nM respectively, so when immunizing with 7MUT followed by 3MUT, the affinity drop would be 1,600/3 = 530). One expects that a boost immunogen with very different epitope structure from the previous immunogen might result in too large an affinity drop to activate memory B cells generated by the prior immunogen. We estimated the affinity drops for all seven boosting schemes (Figure 7E), ranked them according to the largest affinity drop in that boosting scheme, and listed the three most likely to succeed (Figure 7F).

In collaborating work, Escolano et al. (2016) evaluated boosting schemes following the 10MUT trimer prime in PGT121 germline (GL_CDR3-rev4_) knockin mice and PGT121 mature-heavy-and-germline-light-chain knockin mice. Relying on the directionality of the boosting immunogens developed here, Escolano et al. used serum ELISA against boost candidates after each immunization to select the most native-like directional boost for which at least weak serum reactivity could be detected; that process resulted in the testing of the second scheme in Figure 7F and the finding that this scheme induces PGT121-like bnAbs with substantial breadth and potency. Although the first scheme in Figure 7F remains to be tested, the data in Escolano et al. support the validity of the logic underlying these boosting schemes.

We note that the affinity drop analysis also provides clues as to how to improve boosting schemes: to minimize the probability of a boost failure at a high affinity drop, one could redesign immunogens to equalize the affinity drops in any given scheme. Thus the germline-targeting design process is capable of defining potential boost immunogens and directional boosting schemes, and it can guide prioritization and improvement of such schemes.

## Discussion

Germline-targeting vaccine design offers the potential to initiate the induction of specific classes of protective antibodies against HIV or other pathogens that have eluded vaccine development. Many protective bnAbs against HIV are directed toward glycan-dependent epitopes on the trimeric glycoprotein spike (Burton and Hangartner, 2016, Mascola and Haynes, 2013, West et al., 2014). Therefore, methods are needed to develop trimer immunogens for germline targeting and boosting of glycan-dependent bnAbs. Trimer immunogens should be stabilized, to maximize the probability of retaining native-like conformational epitopes in vivo and to minimize the probability of eliciting non-neutralizing Abs that could potentially detract from priming or boosting the targeted bnAb responses.

Here, we (1) developed a mammalian-cell-surface-display directed-evolution method for optimization of multimeric antigens bearing human glycans; (2) engineered stabilized HIV Env trimers with affinity for both germline and mature PGT121-class glycan-dependent bnAbs; (3) showed by crystallography and EM that these trimers maintain native-like conformations; (4) demonstrated that germline-targeting trimers multimerized on liposomes potently activate PGT121 germline and mature B cells ex vivo; and (5) showed that soluble germline-targeting trimers can prime PGT121-class responses in vivo, in a PGT121 inferred-germline knockin mouse. Our data indicate that 11MUT_B_ trimers and trimer-liposomes are promising candidates for priming PGT121-class glycan-dependent bnAb responses in immune systems with diverse antibody repertoires, although the frequency of PGT121-class precursors in humans and the germline-targeting affinities and/or avidities necessary to prime those precursors remain to be determined.

This work could provide a more general template for HIV bnAb germline-targeting than previous work on germline-targeting for VRC01-class bnAbs directed to the CD4-binding site. VRC01-class bnAbs generally do not depend on glycans for their activity, evidenced by the fact that elimination of glycans surrounding the VRC01 epitope generally increases neutralization potency (Jardine et al., 2016b); this has led to removal of all epitope-proximal native glycans from germline-targeting candidates (Jardine et al., 2013, Jardine et al., 2015, Jardine et al., 2016a, McGuire et al., 2013, McGuire et al., 2014, McGuire et al., 2016). However, the activity of many HIV bnAbs requires engagement of one or more glycans within their epitope, and germline-targeting primes should probably retain such key glycans, as was the case here with the N332, N301, and N156 glycans. Furthermore, owing to the relative inaccessibility of the VRC01 epitope on native-like trimers, efforts to design VRC01-class germline-targeting primes have converged on strategies to increase epitope exposure by presentation on minimal domains rather than on trimers (Jardine et al., 2013, Jardine et al., 2015, Jardine et al., 2016a, McGuire et al., 2013, McGuire et al., 2016), although boosting with native-like trimers is anticipated to be required to mature the response (Jardine et al., 2016b). In contrast, many bnAb proteo-glycan epitopes are well exposed on native-like trimers, and some are formed only on intact trimers, making native-like trimers like those designed here the preferred platform for germline targeting. Indeed, multiple different bnAbs could potentially be primed with a single trimer harboring multiple germline-targeting epitopes.

Because germline-targeting vaccine design requires developing not only the vaccine prime but also boost immunogens to mature the response in order to elicit bnAbs, we developed both a stabilized native-like trimer (MD39) and a cocktail of native-like trimers (VLC) that could be employed as boosts to potentially refine and expand the breadth of responses initiated by a germline-targeting prime. However, considering that memory B cells induced by the germline-targeting prime might not be sufficiently mutated to be boosted by a native-like trimer, intermediate boosts might be needed to mature the response prior to native-like boosts. In the process of developing PGT121-class germline-targeting immunogens, we created design intermediates with increasing levels of epitope modification between wild-type and germline-targeting trimers. These molecules are candidate boost immunogens that, if used in sequence, offer directional and gradual epitope changes to guide maturation of the B cell response. We proposed seven potential sequential immunization schemes, and our analysis of affinity drops provided a ranking of those schemes. In a related paper (Escolano et al., 2016), a subset of these prime-boosting schemes were evaluated in PGT121 germline knockin mice and PGT121 mature-heavy-and-germline-light-chain knockin mice, and one such scheme was found to be effective for inducing bnAbs in both mouse models, supporting the germline-targeting vaccine design process described here and encouraging its expanded use and further improvement.

Although here we have described strategies for designing trimer immunogens with changes in the structure of an epitope in order to prime and mature an epitope-specific response, the ultimate success of these strategies might also require modification of antigenic surfaces outside the epitope of interest, to minimize boosting of off-target responses that might hinder or interfere with the desired epitope-specific response.

The approaches employed here could be adapted for immunogen design to other bnAb targets on HIV and other pathogens. The “bootstrapping” strategy of using partially mutated antibodies (such as GL+3 and GL+9) as initial selection agents and then using antibodies closer to germline in successive iterations could be useful for design of germline-targeting and boosting immunogens for other bnAbs, such as HIV V2 Apex glycan-dependent bnAbs (Andrabi et al., 2015, Gorman et al., 2016) or influenza virus hemagglutinin stem-directed bnAbs. Our mammalian display methods allowing directed evolution on native-like trimers should be useful in those endeavors and could also be used to stabilize monomeric or multimeric glycoprotein immunogens for diverse viral vaccines.

In summary, we have developed stabilized native-like trimer immunogens for germline-targeting and boosting of glycan-dependent PGT121-class bnAb responses against HIV. The immunogens and boosting schemes we created are candidates for human vaccine testing and further optimization, and the methods developed here are applicable to immunogen design for other epitopes and pathogens and thus are of relevance for future vaccine design.

## Experimental Procedures

### DNA Gene Synthesis and Protein Production

Genes were synthesized by GenScript. gp120s, gp140s, antigen binding fragments (Fabs), and IgGs were expressed in 293 cells and purified as described in the Supplemental Experimental Procedures.

### Library Assembly

The BG505 SOSIP whole-gene saturation mutagenesis and “rare” amino acid libraries were synthesized by Integrated DNA Technologies and GenScript, respectively. Libraries for germline targeting were created by error-prone PCR (GeneMorph II, Agilent), site-directed mutagenesis (QuikChange, Agilent) or two-step assembly PCR of degenerate primers with the Q5 High-Fidelity DNA Polymerase (New England Biolabs) and cloned into a modified version of the gateway cloning entry vector pENTR/D-TOPO (Ota et al., 2012) with the circular polymerase extension cloning (CPEC) method (Quan and Tian, 2014) or Gibson Assembly (New England Biolabs), according to the manufacturer’s instructions. All libraries were then transferred to the lentiviral vector pLenti CMVTRE3G puro Dest (Ota et al., 2012) with the LR Clonase II enzyme mix (Thermo Scientific).

### Lentivirus Production and Stable Cell Generation

293T cells cultured in Advanced DMEM (GIBCO) supplemented with 5% fetal calf serum, GlutaMAX (GIBCO), 2-mercaptoethanol (GIBCO), and Antibiotic-Antimycotic (GIBCO) were co-transfected with 10.8 μg pLenti CMVTRE3G puro Dest gene library, 7.0 μg psPAX2, and 3.8 μg pMD2.G, as previously described (Salmon and Trono, 2007). 293T cells stably expressing rtTA3G from the pLenti CMV rtTA3G Blast vector (Ota et al., 2012) were transduced at low MOI (< 0.1) in a T75 or T225 flask in the presence of 10 μg/mL blasticidin and, after 24 hr, were transferred to medium supplemented with 2 μg/mL puromycin.

### Cell Surface Expression and FACS

293T cells containing the stable library were induced with doxycycline (1 μg/mL) and harvested the next day in fluorescence-activated cell sorting (FACS) buffer (HBSS, 1 mM EDTA, 0.5% BSA). Cells containing BG505-SOSIP libraries were transfected with furin 24 hr prior to induction. Cells were stained with IgGs or Fabs for ∼30 min, washed with FACS buffer, and then stained with fluorescein isothiocyanate (FITC)-labeled α-cMyc (Immunology Consultants Laboratory). IgGs were labeled with phycoerythrin (PE)-conjugated α-human IgG (Sigma), Fabs containing HA epitope tags (PGT145, PGT151, and PG16) were labeled with α-HA-PE (Miltenyi Biotec), and Fabs containing V5 epitope tags (B6 and 4025) were labeled with α-V5-FITC (GeneTex). Cells were sorted on a BD Influx (BD Biosciences) FACS sorter. Approximately 2 × 10^5^ double positive cells were collected and expanded for approximately one week in the presence of puromycin and blasticidin before the next round of enrichment. Once the desired population had been obtained, chromosomal DNA was extracted from the cell culture with the GenElute Mammalian Genomic DNA Miniprep Kit (Sigma). The gp120 or gp140 gene was PCR amplified from the genomic DNA and inserted back into the pENTR vector via CPEC cloning or Gibson Assembly and transformed into TOP10 competent cells (Invitrogen); colonies were sequenced at Genewiz.

### Next-Generation Sequencing

Sequencing and bioinformatic analysis of the BG505-SOSIP whole-gene saturation mutagenesis libraries were done essentially as described previously (Jardine et al., 2016a).

### Trimer-Conjugated Liposome Synthesis and Characterization

Unilamellar liposomes comprised of DSPC:cholesterol:DGS-NTA(Ni) lipids in a 66.5:28.5:5 mole ratio were synthesized by lipid film rehydration and membrane extrusion, followed by post-synthesis binding of 6xHis-tagged trimer for 2 hr at 4°C. Unconjugated trimer was removed by size exclusion chromatography. Total conjugated trimer was quantified by ELISA in the presence of 1% triton-X and 100 mM imidazole to fully disrupt liposomes and Ni-6xHis interactions, respectively, for uninhibited detection via an α-6xHis antibody. Antigenic profiles were determined by ELISA on intact liposomes.

### Ca^2+^-Flux Measurements and Immunizations

Details about Ca^2+^-flux assays and mouse immunizations can be found in the Supplemental Experimental Procedures and in Escolano et al., 2016.

### Negative-Stain EM

Purified SOSIP trimers were analyzed by negative-stain EM with a protocol adapted from de Taeye et al., 2016.

### DSC and SPR Methods

Differential scanning calorimetry (DSC) and surface plasmon resonance (SPR) methods are described in the Supplemental Experimental Procedures.

### Crystallization and Data Collection

Description of crystallization, data collection, and refinement can be found in the Supplemental Experimental Procedures. Statistics for data collection and final refinement are listed in Table S2.

## Author Contributions

J.M.S., D.W.K., and W.R.S. conceived the immunogen design strategies, developed mammalian display, and designed immunogens and boosting schemes. L.E.M., B.B., and D.R.B. assisted with mammalian display directed evolution and provided intermediate mutated Abs for selection agents. S.M. and D.W.K. carried out loop and glycan modeling. A.E. and M.C.N. provided the plasmid for GL_CDR3-rev4_. Y.A., M.K., E.G., M.J., and D.W.K. produced immunogens and Abs. O.K., X.H., T.S., L.E.M., J.M.S., and D.W.K. characterized immunogens and Abs with biophysical analysis methods. R.L.S., F.G., I.A.W., G.O., and A.B.W. characterized immunogens and Abs with structural analysis methods. T.T., D.J.I., D.W.K., and W.R.S. conceived trimer-liposome immunogens. T.T., A.M., D.S.Y., and D.J.I. developed and characterized trimer liposomes by using biophysical and structural analysis. N.T.F., A.D.G., A.E., and M.C.N. created and characterized PGT121 GL knockin mice, encouraging germline-targeting efforts. P.D., M.C.N., J.M.S., T.T., D.J.I., D.W.K., W.R.S planned ex vivo B cell activation studies. P.D., C.T.M., and M.C.N. performed and analyzed ex vivo B cell activation studies. A.E. and M.C.N. planned, performed, and analyzed immunization studies. J.M.S., D.W.K., and W.R.S. wrote the paper. M.C.N., D.J.I., D.R.B., I.A.W., A.B.W., T.T., R.L.S., L.E.M., G.O., X.H., O.K., and T.S. helped write the paper.

## Accession Numbers

The accession number for the coordinates and structure factors for MD39-10MUT_A_ in complex with 35O22 and PGT124 is PDB: 5T3S.

## Supplemental Information

Document S1. Supplemental Experimental Procedures, Figures S1–S7, and Tables S1–S3 Document S1. Supplemental Experimental Procedures, Figures S1–S7, and Tables S1–S3

Document S2. Article plus Supplemental Information Document S2. Article plus Supplemental Information
