# Supplementary material for: HIV Vaccine Design to Target Germline Precursors of Glycan-Dependent Broadly Neutralizing Antibodies
Source: Immunity. 2016 Sep 20;45(3):483–96. doi: 10.1016/j.immuni.2016.08.016 (PMC5040827; doi:10.1016/j.immuni.2016.08.016)
Supplement: Document S1. Supplemental Experimental Procedures, Figures S1–S7, and Tables S1–S3 [file mmc1.pdf]

Immunity, Volume *45*

Supplemental Information

HIV Vaccine Design to Target Germline Precursors of Glycan-Dependent Broadly Neutralizing Antibodies

Jon M. Steichen, Daniel W. Kulp, Talar Tokatlian, Amelia Escolano, Pia Dosenovic, Robyn L. Stanfield, Laura E. McCoy, Gabriel Ozorowski, Xiaozhen Hu, Oleksandr Kalyuzhniy, Bryan Briney, Torben Schiffner, Fernando Garces, Natalia T. Freund, Alexander D. Gitlin, Sergey Menis, Erik Georgeson, Michael Kubitz, Yumiko Adachi, Meaghan Jones, Andrew A. Mutafyan, Dong Soo Yun, Christian T. Mayer, Andrew B. Ward, Dennis R. Burton, Ian A. Wilson, Darrell J. Irvine, Michel C. Nussenzweig, and William R. Schief

Figure S1

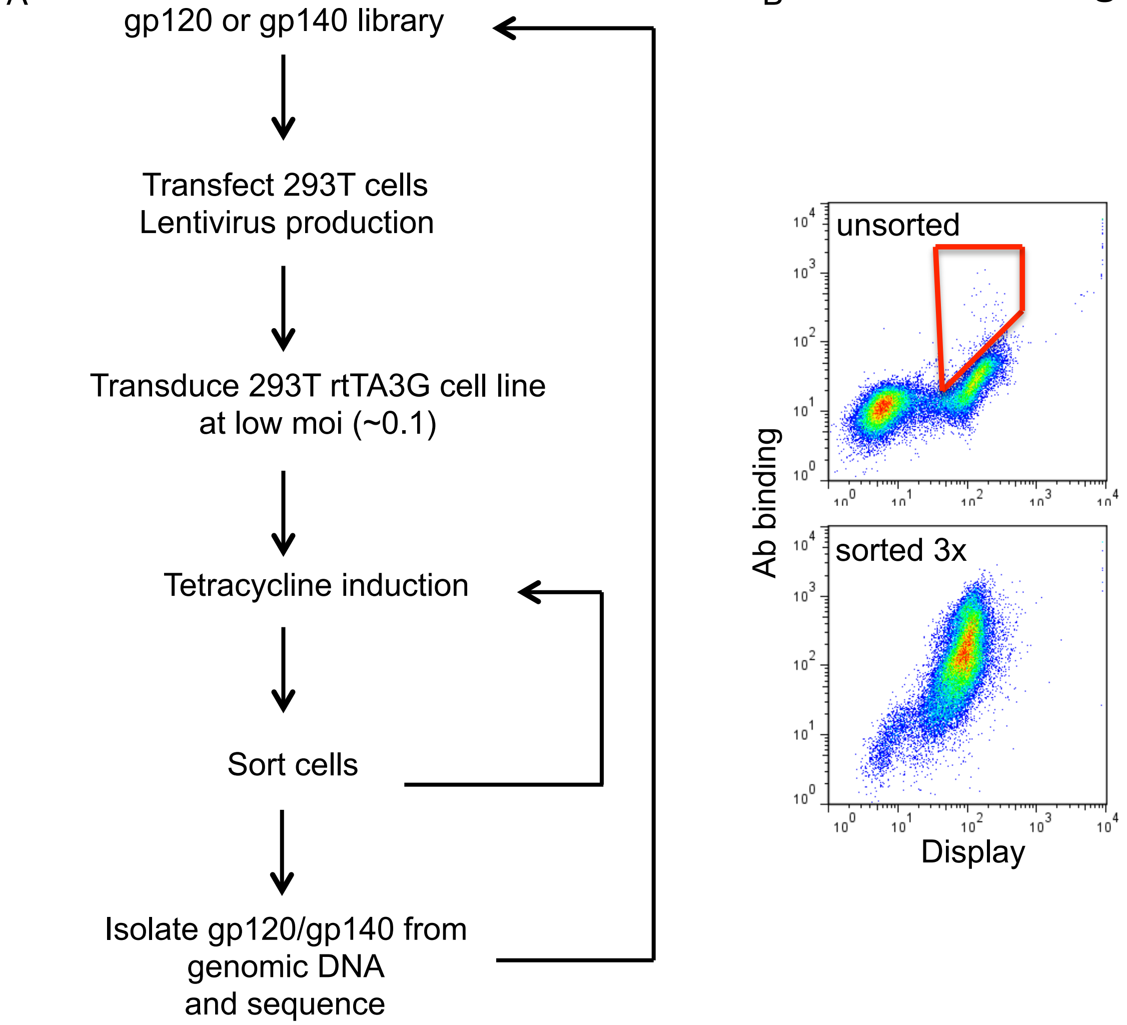

C

BG505-SOSIP.664_PDGFR_TM
MDAMKRGLCCVLLLCGAVFVSPSQEIHARFRRGARAENLWVTVYYGVPVWKDAETTLFCASDAKAYETEKHNVWATHACVPTDPNPQEIHLENVTEEFN
MWKNNMVEQMHTDIISLWDQSLKPCVKLTPLCVTLQCTNVTNNITDDMRGELKNCSFNMTTELRDKKQKVYSLFYRLDVVQINENQGNRSNNSNKEYRL
INCNTSAITQACPKVSFEPIPIHYCAPAGFAILKCKDKKFNGTGPCPSVSTVQCTHGIKPVVSTQLLLNGSLAEEEVMIRSENITNNAKNILVQFNTPV
QINCTRPNNNTRKSIRIGPGQAFYATGDIIGDIRQAHCNVSKATWNETLGKVVKQLRKHFGNNTIIRFANSSGGDLEVTTHSFNCGGEFFYCNTSGLFN
STWISNTSVQGSNSTGSNDSITLPCRIKQIINMWQRIGQAMYAPPIQGVIRCVSNITGLILTRDGGSTNSTTETFRPGGGDMRDNWRSELYKYKVVKIE
PLGVAPTRCKRRVVGRRRRRRAVGIGAVFLGFLGAAGSTMGAASMTLTVQARNLLSGIVQQQSNLLRAPEAQQHLLKLTVWGIKQLQARVLAVERYLRD
QQLLGIWGCSGKLICCTNVPWNSSWSNRNLSEIWDNMTWLQWDKEISNYTQIIYGLLEESQNQQEKNEQDLLALDGGGSGGSGGSEQKLISEEDLGGSG
GSGGSNAVGQDTQEVIVVPHSLPFKVVVISAILALVVLTIISLIILIMLWQKKPR

BG505-gp120_PDGFR_TM
METDTLLLWVLLLWVPGSTGDAENLWVTVYYGVPVWKDAETTLFCASDAKAYETEKHNVWATHACVPTDPNPQEIHLENVTEEFNMWKNNMVEQMHTD
IISLWDQSLKPCVKLTPLCVTLQCTNVTNNITDDMRGELKNCSFNMTTELRDKKQKVYSLFYRLDVVQINENQGNRSNNSNKEYRLINCNTSAITQAC
PKVSFEPIPIHYCAPAGFAILKCKDKKFNGTGPCPSVSTVQCTHGIKPVVSTQLLLNGSLAEEEVMIRSENITNNAKNILVQFNTPVQINCTRPNNNT
RKSIRIGPGQAFYATGDIIGDIRQAHCNVSKATWNETLGKVVKQLRKHFGNNTIIRFANSSGGDLEVTTHSFNCGGEFFYCNTSGLFNSTWISNTSVQ
GSNSTGSNDSITLPCRIKQIINMWQRIGQAMYAPPIQGVIRCVSNITGLILTRDGGSTNSTTETFRPGGGDMRDNWRSELYKYKVVKIEPLGVAPTRA
KRRVVGGGSGGSGGSEQKLISEEDLGGSGGSGGSNAVGQDTQEVIVVPHSLPFKVVVISAILALVVLTIISLIILIMLWQKKPR

Figure S2

Heavy Chain

```
                 V
V4-59/D3-3/J6    QVQLQESGPGLVKPSETLSLTCTVSGGSISSYYWSWIRQPPGKGLEWIGYIYYSGSTNYNPSLKSRVTIS
GL-CDR3rev1      QVQLQESGPGLVKPSETLSLTCTVSGGSISSYYWSWIRQPPGKGLEWIGYIYYSGSTNYNPSLKSRVTIS
GL-CDR3rev2      QVQLQESGPGLVKPSETLSLTCTVSGGSISSYYWSWIRQPPGKGLEWIGYIYYSGSTNYNPSLKSRVTIS
GL-CDR3rev3      QVQLQESGPGLVKPSETLSLTCTVSGGSISSYYWSWIRQPPGKGLEWIGYIYYSGSTNYNPSLKSRVTIS
GL-CDR3rev4      QVQLQESGPGLVKPSETLSLTCTVSGGSISSYYWSWIRQPPGKGLEWIGYIYYSGSTNYNPSLKSRVTIS
GL-CDR3rev5      QVQLQESGPGLVKPSETLSLTCTVSGGSISSYYWSWIRQPPGKGLEWIGYIYYSGSTNYNPSLKSRVTIS
GL+3             QVQLQESGPGLVKPSETLSLTCTVSGGSISSYYWSWIRQPPGKGLEWIGYIYYSGSTNYNPSLKSRVTIS
GL-CDR3mat       QVQLQESGPGLVKPSETLSLTCTVSGGSISSYYWSWIRQPPGKGLEWIGYIYYSGSTNYNPSLKSRVTIS
GL+9             QVQLQESGPGLVKPSETLSLTCTVSGGSISSYYWSWIRQPPGKGLEWIGYIYYSGSTNYNPSLKSRVTIS
PGT121           QMQLQESGPGLVKPSETLSLTCSVSGASISDSYWSWIRRSPGKGLEWIGYVHKSGDTNYSPSLKSRVNLS

                 D   J
V4-59/D3-3/J6    VDTSKNQFSLKLSSVTAADTAVYYCAR----ITIFGVVII--YYYYYYMDVWGKGTTVTVSS
GL-CDR3rev1      VDTSKNQFSLKLSSVTAADTAVYYCARTLHGITIFGVVAFKEYYYYYYMDVWGKGTTVTVSS
GL-CDR3rev2      VDTSKNQFSLKLSSVTAADTAVYYCARTLHGITIFGVVIIKEYYYYYYMDVWGKGTTVTVSS
GL-CDR3rev3      VDTSKNQFSLKLSSVTAADTAVYYCARTQQGKRIYGVVSFGEYYYYYYMDVWGKGTTVTVSS
GL-CDR3rev4      VDTSKNQFSLKLSSVTAADTAVYYCARTQQGKRIYGVVSFGDYYYYYYMDVWGKGTTVTVSS
GL-CDR3rev5      VDTSKNQFSLKLSSVTAADTAVYYCARTLHGITIFGVVAFKEYYYYYYMDVWGKGTTVTVSS
GL+3             VDTSKNQFSLKLSSVTAADTAVYYCARTLHGRRIYGIVAFKEWFTYYYMDVWGKGTTVTVSS
GL-CDR3mat       VDTSKNQFSLKLSSVTAADTAVYYCARTLHGRRIYGIVAFKEWFTYYYMDVWGKGTTVTVSS
GL+9             VDTSKNQFSLKLSSVTAADTAVYYCARTLHGRRIYGIVAFKEWFTYYYMDVWGKGTTVTVSS
PGT121           LDTSKNQVSLSLVAATAADSGKYYCARTLHGRRIYGIVAFNEWFTYFYMDVWGNGTQVTVSS
```

Light Chain

```
                 V
V3-21/J3         SYVLTQPPSVSVAPGQTARITCGGNNIGSKSVHWYQQKPGQAPVLVVYDDSDRPSGIPERFSGSNS---G
GL-CDR3rev1      SYVLTQPPSVSVAPGQTARITCGGNNIGSKSVHWYQQKPGQAPVLVVYDDSDRPSGIPERFSGSNS---G
GL-CDR3rev2      SYVLTQPPSVSVAPGQTARITCGGNNIGSKSVHWYQQKPGQAPVLVVYDDSDRPSGIPERFSGSNS---G
GL-CDR3rev3      SYVLTQPPSVSVAPGQTARITCGGNNIGSKSVHWYQQKPGQAPVLVVYDDSDRPSGIPERFSGSNS---G
GL-CDR3rev4      SYVLTQPPSVSVAPGQTARITCGGNNIGSKSVHWYQQKPGQAPVLVVYDDSDRPSGIPERFSGSNS---G
GL-CDR3rev5      SYVLTQPPSVSVAPGQTARITCGGNNIGSKSVHWYQQKPGQAPVLVVYDDSDRPSGIPERFSGSNS---G
GL+3             SYVLTQPPSVSVAPGKTARITCGGNNIGSKSVHWYQQKPGQAPVLVIYDDSDRPSGIPERFSGSNS---G
GL-CDR3mat       SYVLTQPPSVSVAPGQTARITCGGNNIGSKSVHWYQQKPGQAPVLVVYDDSDRPSGIPERFSGSNS---G
GL+9             SYVLTQPPSVSVAPGETARISCGGNNIGSKSVQWYQQKPGQAPVLVVYNNQDRPSGIPERFSGSPD---G
PGT121           -------SDISVAPGETARISCGEKSLGSRAVQWYQHRAGQAPSLIIYNNQDRPSGIPERFSGSPDSPFG

                 J
V3-21/J3         NTATLTISRVEAGDEADYYCQVWDSSSDHPWVFGGGTKLTVL
GL-CDR3rev1      NTATLTISRVEAGDEADYYCQVWDSSSDHPWVFGGGTKLTVL
GL-CDR3rev2      NTATLTISRVEAGDEADYYCQVWDSRGPTNWVFGGGTKLTVL
GL-CDR3rev3      NTATLTISRVEAGDEADYYCQVWDSSSDHPWVFGGGTKLTVL
GL-CDR3rev4      NTATLTISRVEAGDEADYYCQVWDSSSDHPWVFGGGTKLTVL
GL-CDR3rev5      NTATLTISRVEAGDEADYYCQVWDSRGPTNWVFGGGTKLTVL
GL+3             NTATLTISRVEAGDEADYYCQVWDSRSDHPWVFGGGTKLTVL
GL-CDR3mat       NTATLTISRVEAGDEADYYCQVWDSRGPTNWVFGGGTKLTVL
GL+9             NTATLTISRVEAGDEADYYCQVWDSRSDHPWVFGGGTKLTVL
PGT121           TTATLTITSVEAGDEADYYCHIWDSRVPTKWVFGGGTTLTVL
```

Figure S3

```
BG505_SOSIP.664           AENLWVTVYYGVPVWKDAETTLFCASDAKAYETEKHNVWATHACVPTDPNPQEIHLENVTEEFNMWKNNMVEQMHTDIISLWDQSLKPCVK
BG505_SOSIP.664_2MUT      AENLWVTVYYGVPVWKDAETTLFCASDAKAYETEKHNVWATHACVPTDPNPQEIHLENVTEEFNMWKNNMVEQMHTDIISLWDQSLKPCVK
BG505_SOSIP.664_3MUT      AENLWVTVYYGVPVWKDAETTLFCASDAKAYETEKHNVWATHACVPTDPNPQEIHLENVTEEFNMWKNNMVEQMHTDIISLWDQSLKPCVK
BG505_SOSIP.664_5MUT      AENLWVTVYYGVPVWKDAETTLFCASDAKAYETEKHNVWATHACVPTDPNPQEIHLENVTEEFNMWKNNMVEQMHTDIISLWDQSLKPCVK
BG505_SOSIP.664_6MUT      AENLWVTVYYGVPVWKDAETTLFCASDAKAYETEKHNVWATHACVPTDPNPQEIHLENVTEEFNMWKNNMVEQMHTDIISLWDQSLKPCVK
BG505_SOSIP.664_7MUT      AENLWVTVYYGVPVWKDAETTLFCASDAKAYETEKHNVWATHACVPTDPNPQEIHLENVTEEFNMWKNNMVEQMHTDIISLWDQSLKPCVK
BG505_SOSIP.664_9MUTA     AENLWVTVYYGVPVWKDAETTLFCASDAKAYETEKHNVWATHACVPTDPNPQEIHLENVTEEFNMWKNNMVEQMHTDIISLWDQSLKPCVK
BG505_gp120_9MUTB         -------------VWKDAETTLFCASDAKAYETEKHNVWATHACVPTDPNPQEIHLENVTEEFNMWKNNMVEQMHTDIISAWDQSLKPCVK
BG505_gp120_10MUT_KO      -------------VWKDAETTLFCASDAKAYETEKHNVWATHACVPTDPNPQEIHLENVTEEFNMWKNNMVEQMHTDIISAWDQSLKPCVK
BG505_gp120_10MUT         -------------VWKDAETTLFCASDAKAYETEKHNVWATHACVPTDPNPQEIHLENVTEEFNMWKNNMVEQMHTDIISAWDQSLKPCVK
BG505_SOSIP.664_10MUT     AENLWVTVYYGVPVWKDAETTLFCASDAKAYETEKHNVWATHACVPTDPNPQEIHLENVTEEFNMWKNNMVEQMHTDIISLWDQSLKPCVK
BG505_SOSIP_MD39_10MUTA   AENLWVTVYYGVPVWKDAETTLFCASDAKAYETEKHNVWATHACVPTDPNPQEIHLENVTEEFNMWKNNMVEQMHEDIISLWDQSLKPCVK
BG505_SOSIP.664_11MUTA    AENLWVTVYYGVPVWKDAETTLFCASDAKAYETEKHNVWATHACVPTDPNPQEIHLENVTEEFNMWKNNMVEQMHTDIISLWDQSLKPCVK
BG505_SOSIP_MD39_11MUTB   AENLWVTVYYGVPVWKDAETTLFCASDAKAYETEKHNVWATHACVPTDPNPQEIHLENVTEEFNMWKNNMVEQMHEDIISLWDQSLKPCVK

BG505_SOSIP.664           LTPLCVTLQCTNVTNNITDDMRGELKNCSFNMTTELRDKKQKVYSLFYRLDVVQINENQGNRSNNSNKEYRLINCNTSAITQACPKVSFEP
BG505_SOSIP.664_2MUT      LTPLCVTLQCTNVTNNIIDDMRGELKNCSFNMTTELRDKKQKVYSLFYRLDVVQINENQGNRSNNSNKEYRLINCNTSAITQACPKVSFEP
BG505_SOSIP.664_3MUT      LTPLCVTLQCTNVANNIIDDMRGELKNCSFNMTTELRDKKQKVYSLFYRLDVVQINENQGNRSNNSNKEYRLINCNTSAITQACPKVSFEP
BG505_SOSIP.664_5MUT      LTPLCVTLQCTNYTPNLTNDMRGELKNCSFNMTTELRDKKQKVYSLFYRLDVVQINENQGNRSNNSNKEYRLINCNTSAITQACPKVSFEP
BG505_SOSIP.664_6MUT      LTPLCVTLQCTNYTPNLINDMRGELKNCSFNMTTELRDKKQKVYSLFYRLDVVQINENQGNRSNNSNKEYRLINCNTSAITQACPKVSFEP
BG505_SOSIP.664_7MUT      LTPLCVTLQCTNYAPNLINDMRGELKNCSFNMTTELRDKKQKVYSLFYRLDVVQINENQGNRSNNSNKEYRLINCNTSAITQACPKVSFEP
BG505_SOSIP.664_9MUTA     LTPLCVTLQCTNYAPFLINDMRGELKNCSFNMTTELRDKKQKVYSLFYRLDVVQINENQGNRSNNSNKEYRLINCNTSAITQACPKVSFEP
BG505_gp120_9MUTB         LTPLCVTLQCTNYRPNLINDMRGELKNCSFNMTTELRDKKQKVYSLFYRLDVVQINENQGNRSNNSNKEYRLINCNTSAITQACPKVSFEP
BG505_gp120_10MUT_KO      LTPLCVTLQCTNYAPFLINDMRGELKNCSFNMTTELRDKKQKVYSLFYRLDVVQINENQGNRSNNSNKEYRLINCNTSAITQACPKVSFEP
BG505_gp120_10MUT         LTPLCVTLQCTNYAPFLINDMRGELKNCSFNMTTELRDKKQKVYSLFYRLDVVQINENQGNRSNNSNKEYRLINCNTSAITQACPKVSFEP
BG505_SOSIP.664_10MUT     LTPLCVTLQCTNYAPFLINDMRGELKNCSFNMTTELRDKKQKVYSLFYRLDVVQINENQGNRSNNSNKEYRLINCNTSAITQACPKVSFEP
BG505_SOSIP_MD39_10MUTA   LTPLCVTLQCTNYAPFLINNMRGELKNCSFNMTTELRDKKQKVYSLFYRLDVVQINENQGNRSNNSNKEYRLINCNTSAITQACPKVSFEP
BG505_SOSIP.664_11MUTA    LTPLCVTLQCTNYAPFLINNMRGELKNCSFNMTTELRDKKQKVYSLFYRLDVVQINENQGNRSNNSNKEYRLINCNTSAITQACPKVSFEP
BG505_SOSIP_MD39_11MUTB   LTPLCVTLQCTNYAPNLLSNMRGELKNCSFNMTTELRDKKQKVYSLFYRLDVVQINENQGNRSNNSNKEYRLINCNTSAITQACPKVSFEP

BG505_SOSIP.664           IPIHYCAPAGFAILKCKDKKFNGTGPCPSVSTVQCTHGIKPVVSTQLLLNGSLAEEEVMIRSENITNNAKNILVQFNTPVQINCTRPNNNT
BG505_SOSIP.664_2MUT      IPIHYCAPAGFAILKCKDKKFNGTGPCPSVSTVQCTHGIKPVVSTQLLLNGSLAEEEVMIRSENITNNAKNILVQFNTPVQINCTRPNNNT
BG505_SOSIP.664_3MUT      IPIHYCAPAGFAILKCKDKKFNGTGPCPSVSTVQCTHGIKPVVSTQLLLNGSLAEEEVMIRSENITNNAKNILVQFNTPVQINCTRPNNNT
BG505_SOSIP.664_5MUT      IPIHYCAPAGFAILKCKDKKFNGTGPCPSVSTVQCTHGIKPVVSTQLLLNGSLAEEEVMIRSENITNNAKNILVQFNTPVQINCTRPNNNT
BG505_SOSIP.664_6MUT      IPIHYCAPAGFAILKCKDKKFNGTGPCPSVSTVQCTHGIKPVVSTQLLLNGSLAEEEVMIRSENITNNAKNILVQFNTPVQINCTRPNNNT
BG505_SOSIP.664_7MUT      IPIHYCAPAGFAILKCKDKKFNGTGPCPSVSTVQCTHGIKPVVSTQLLLNGSLAEEEVMIRSENITNNAKNILVQFNTPVQINCTRPNNNT
BG505_SOSIP.664_9MUTA     IPIHYCAPAGFAILKCKDKKFNGTGPCPSVSTVQCTHGIKPVVSTQLLLNGSLAEEEVMIRSENITNNAKNILVQFNTPVQINCTRPNNNT
BG505_gp120_9MUTB         IPIHYCAPAGFAILKCKDKKFNGTGPCPSVSTVQCTHGIKPVVSTQLLLNGSLAEEEVMIRSENITNNAKNILVQFNTPVQINCTRPNNNT
BG505_gp120_10MUT_KO      IPIHYCAPAGFAILKCKDKKFNGTGPCPSVSTVQCTHGIKPVVSTQLLLNGSLAEEEVMIRSENITNNAKNILVQFNTPVQINCTRPNNNA
BG505_gp120_10MUT         IPIHYCAPAGFAILKCKDKKFNGTGPCPSVSTVQCTHGIKPVVSTQLLLNGSLAEEEVMIRSENITNNAKNILVQFNTPVQINCTRPNNNT
BG505_SOSIP.664_10MUT     IPIHYCAPAGFAILKCKDKKFNGTGPCPSVSTVQCTHGIKPVVSTQLLLNGSLAEEEVMIRSENITNNAKNILVQFNTPVQINCTRPNNNT
BG505_SOSIP_MD39_10MUTA   IPIHYCAPAGFAILKCKDKKFNGTGPCPSVSTVQCTHGIKPVVSTQLLLNGSLAEEEVIIRSENITNNAKNILVQLNTPVQINCTRPNNNT
BG505_SOSIP.664_11MUTA    IPIHYCAPAGFAILKCKDKKFNGTGPCPSVSTVQCTHGIKPVVSTQLLLNGSLAEEEVMIRSENITNNAKNILVQFNTPVQINCTRPNNNT
BG505_SOSIP_MD39_11MUTB   IPIHYCAPAGFAILKCKDKKFNGTGPCPSVSTVQCTHGIKPVVSTQLLLNGSLAEEEVIIRSENITNNAKNILVQLNTPVQINCTRPNNNT

BG505_SOSIP.664           RKSIRIGPGQAFYATGDIIGDIRQAHCNVSKATWNETLGKVVKQLRKHFGNNTIIRFANSSGGDLEVTTHSFNCGGEFFYCNTSGLFNSTW
BG505_SOSIP.664_2MUT      RKSIRIGPGQAFYATGDIIGDIRQAHCNVSKATWNETLGKVVKQLRKHFGNNTIIRFANSSGGDLEVTTHSFNCGGEFFYCNTSGLFNSTW
BG505_SOSIP.664_3MUT      RKSIRIGPGQAFYATGDIIGDIRQAHCNVSKATWNETLGKVVKQLRKHFGNNTIIRFANSSGGDLEVTTHSFNCGGEFFYCNTSGLFNSTW
BG505_SOSIP.664_5MUT      RKSIRIGPGQAFYATGDIIGDIRQAHCNVSKATWNETLGKVVKQLRKHFGNNTIIRFANSSGGDLEVTTHSFNCGGEFFYCNTSGLFNSTW
BG505_SOSIP.664_6MUT      RKSIRIGPGQAFYATGDIIGDIRQAHCNVSKATWNETLGKVVKQLRKHFGNNTIIRFANSSGGDLEVTTHSFNCGGEFFYCNTSGLFNSTW
BG505_SOSIP.664_7MUT      RKSIRIGPGQAFYATGDIIGDIRQAHCNVSKATWNETLGKVVKQLRKHFGNNTIIRFANSSGGDLEVTTHSFNCGGEFFYCNTSGLFNSTW
BG505_SOSIP.664_9MUTA     RKSIRIGPGQAFYATGDIIGDIRMAHCNVSKATWNETLGKVVKQLRKHFGNNTIIRFANSSGGDLEVTTHSFNCGGEFFYCNTSGLFNSTW
BG505_gp120_9MUTB         RKSIRIGPGQAFYATGDIIGDIRMAHCNVSKATWNETLGKVVKQLRKHFGNNTIIRFANSSGGDLEVTTHSFNCGGEFFYCNTSGLFNSTW
BG505_gp120_10MUT_KO      RKSIRIGPGQAFYAFGDIIGDIRMAACTVSKATWNETLGKVVKQLRKHFGNNTIIRFANSSGGDLEVTTHSFNCGGEFFYCNTSGLFNSTW
BG505_gp120_10MUT         RKSIRIGPGQAFYAFGDIIGDIRMAHCNVSKATWNETLGKVVKQLRKHFGNNTIIRFANSSGGDLEVTTHSFNCGGEFFYCNTSGLFNSTW
BG505_SOSIP.664_10MUT     RKSIRIGPGQAFYAFGDIIGDIRMAHCNVSKATWNETLGKVVKQLRKHFGNNTIIRFANSSGGDLEVTTHSFNCGGEFFYCNTSGLFNSTW
BG505_SOSIP_MD39_10MUTA   VKSIRIGPGQAFYYTGDIIGDIRMAHCNVSKATWNETLGKVVKQLRKHFGNNTIIRFAQSSGGDLEVTTHSFNCGGEFFYCNTSGLFNSTW
BG505_SOSIP.664_11MUTA    RKSIRIGPGQAFYAFGDIIGDIRMAHCNVSKATWNETLGKVVKQLRKHFGNNTIIRFANSSGGDLEVTTHSFNCGGEFFYCNTSGLFNSTW
BG505_SOSIP_MD39_11MUTB   VKSIRIGPGQAFYYFGDIIGDIRMAHCNVSKATWNETLGKVVKQLRKHFGNNTIIRFAQSSGGDLEVTTHSFNCGGEFFYCNTSGLFNSTW

BG505_SOSIP.664           ISNTSVQGSNSTGSNDSITLPCRIKQIINMWQRIGQAMYAPPIQGVIRCVSNITGLILTRDGGSTNSTTETFRPGGGDMRDNWRSELYKYK
BG505_SOSIP.664_2MUT      ISNTSVQGSNSTGSNDSITLPCRIKQIINMWQRIGQAMYAPPIQGVIRCVSNITGLILTRDGGSTNSTTETFRPGGGDMRDNWRSELYKYK
BG505_SOSIP.664_3MUT      ISNTSVQGSNSTGSNDSITLPCRIKQIINMWQRIGQAMYAPPIQGVIRCVSNITGLILTRDGGSTNSTTETFRPGGGDMRDNWRSELYKYK
BG505_SOSIP.664_5MUT      ISNTSVQGSNSTGSNDSITLPCRIKQIINMWQRIGQAMYAPPIQGVIRCVSNITGLILTRDGGSTNSTTETFRPGGGDMRDNWRSELYKYK
BG505_SOSIP.664_6MUT      ISNTSVQGSNSTGSNDSITLPCRIKQIINMWQRIGQAMYAPPIQGVIRCVSNITGLILTRDGGSTNSTTETFRPGGGDMRDNWRSELYKYK
BG505_SOSIP.664_7MUT      ISNTSVQGSNSTGSNDSITLPCRIKQIINMWQRIGQAMYAPPIQGVIRCVSNITGLILTRDGGSTNSTTETFRPGGGDMRDNWRSELYKYK
BG505_SOSIP.664_9MUTA     ISNTSVQGSNSTGSNDSITLPCRIKQIINMWQRIGQAMYAPPIQGVIRCVSNITGLILTRDGGSTNSTTETFRPGGGDMRDNWRSELYKYK
BG505_gp120_9MUTB         ISNTSVQGSNSTGSNDSITLPCRIKQIINMWQRIGQAMYAPPIQGVIRCVSNITGLILTRDGGSTNSTTETFRPGGGDMRDNWRSELYKYK
BG505_gp120_10MUT_KO      ISNTSVQGSNSTGSNDSITLPCRIKQIINMWQRIGQAMYAPPIQGVIRCVSNITGLILTRDGGSTNSTTETFRPGGGDMRDNWRSELYKYK
BG505_gp120_10MUT         ISNTSVQGSNSTGSNDSITLPCRIKQIINMWQRIGQAMYAPPIQGVIRCVSNITGLILTRDGGSTNSTTETFRPGGGDMRDNWRSELYKYK
BG505_SOSIP.664_10MUT     ISNTSVQGSNSTGSNDSITLPCRIKQIINMWQRIGQAMYAPPIQGVIRCVSNITGLILTRDGGSTNSTTETFRPGGGDMRDNWRSELYKYK
BG505_SOSIP_MD39_10MUTA   ISNTSVQGSNSTGSNDSITLPCRIKQIINMWQRIGQAMYAPPIQGVIRCVSNITGLILTRDGGSTNSTTETFRPGGGDMRDNWRSELYKYK
BG505_SOSIP.664_11MUTA    ISNTSVQGSNSTGSNDSITLPCRIKQIINMWQRIGQAMYAPPIQGVIRCVSNITGLILTRDGGSTNSTTETFRPGGGDMRDNWRSELYKYK
BG505_SOSIP_MD39_11MUTB   ISNTSVQGSNSTGSNDSIVLPCRIKQIINMWQRIGQAMYAPPIQGVIRCVSNITGLILTRDGGSTNSTTETFRPGGGDMRDNWRSELYKYK

BG505_SOSIP.664           VVKIEPLGVAPTRCKRRVVGRRRRRRAVGIGAVFLGFLGAAGSTMGAASMTLTVQARNLLSGIVQQQSNLLRAPEAQQHLLKLTVWGIKQL
BG505_SOSIP.664_2MUT      VVKIEPLGVAPTRCKRRVVGRRRRRRAVGIGAVFLGFLGAAGSTMGAASMTLTVQARNLLSGIVQQQSNLLRAPEAQQHLLKLTVWGIKQL
BG505_SOSIP.664_3MUT      VVKIEPLGVAPTRCKRRVVGRRRRRRAVGIGAVFLGFLGAAGSTMGAASMTLTVQARNLLSGIVQQQSNLLRAPEAQQHLLKLTVWGIKQL
BG505_SOSIP.664_5MUT      VVKIEPLGVAPTRCKRRVVGRRRRRRAVGIGAVFLGFLGAAGSTMGAASMTLTVQARNLLSGIVQQQSNLLRAPEAQQHLLKLTVWGIKQL
BG505_SOSIP.664_6MUT      VVKIEPLGVAPTRCKRRVVGRRRRRRAVGIGAVFLGFLGAAGSTMGAASMTLTVQARNLLSGIVQQQSNLLRAPEAQQHLLKLTVWGIKQL
BG505_SOSIP.664_7MUT      VVKIEPLGVAPTRCKRRVVGRRRRRRAVGIGAVFLGFLGAAGSTMGAASMTLTVQARNLLSGIVQQQSNLLRAPEAQQHLLKLTVWGIKQL
BG505_SOSIP.664_9MUTA     VVKIEPLGVAPTRCKRRVVGRRRRRRAVGIGAVFLGFLGAAGSTMGAASMTLTVQARNLLSGIVQQQSNLLRAPEAQQHLLKLTVWGIKQL
BG505_gp120_9MUTB         VVKIEP-------------------------------------------------------------------------------------
BG505_gp120_10MUT_KO      VVKIEP-------------------------------------------------------------------------------------
BG505_gp120_10MUT         VVKIEP-------------------------------------------------------------------------------------
BG505_SOSIP.664_10MUT     VVKIEPLGVAPTRCKRRVVGRRRRRRAVGIGAVFLGFLGAAGSTMGAASMTLTVQARNLLSGIVQQQSNLLRAPEAQQHLLKLTVWGIKQL
BG505_SOSIP_MD39_10MUTA   VVKIEPLGVAPTRCKRRVVGRRRRRRAVGIGAVSLGFLGAAGSTMGAASMTLTVQARNLLSGIVQQQSNLLRAPEPQQHLLKDTHWGIKQL
BG505_SOSIP.664_11MUTA    VVKIEPLGVAPTRCKRRVVGRRRRRRAVGIGAVFLGFLGAAGSTMGAASMTLTVQARNLLSGIVQQQSNLLRAPEAQQHLLKLTVWGIKQL
BG505_SOSIP_MD39_11MUTB   VVKIEPLGVAPTRCKRRVVGRRRRRRAVGIGAVSLGFLGAAGSTMGAASMTLTVQARNLLSGIVQQQSNLLRAPEPQQHLLKDTHWGIKQL

BG505_SOSIP.664           QARVLAVERYLRDQQLLGIWGCSGKLICCTNVPWNSSWSNRNLSEIWDNMTWLQWDKEISNYTQIIYGLLEESQNQQEKNEQDLLALD
BG505_SOSIP.664_2MUT      QARVLAVERYLRDQQLLGIWGCSGKLICCTNVPWNSSWSNRNLSEIWDNMTWLQWDKEISNYTQIIYGLLEESQNQQEKNEQDLLALD
BG505_SOSIP.664_3MUT      QARVLAVERYLRDQQLLGIWGCSGKLICCTNVPWNSSWSNRNLSEIWDNMTWLQWDKEISNYTQIIYGLLEESQNQQEKNEQDLLALD
BG505_SOSIP.664_5MUT      QARVLAVERYLRDQQLLGIWGCSGKLICCTNVPWNSSWSNRNLSEIWDNMTWLQWDKEISNYTQIIYGLLEESQNQQEKNEQDLLALD
BG505_SOSIP.664_6MUT      QARVLAVERYLRDQQLLGIWGCSGKLICCTNVPWNSSWSNRNLSEIWDNMTWLQWDKEISNYTQIIYGLLEESQNQQEKNEQDLLALD
BG505_SOSIP.664_7MUT      QARVLAVERYLRDQQLLGIWGCSGKLICCTNVPWNSSWSNRNLSEIWDNMTWLQWDKEISNYTQIIYGLLEESQNQQEKNEQDLLALD
BG505_SOSIP.664_9MUTA     QARVLAVERYLRDQQLLGIWGCSGKLICCTNVPWNSSWSNRNLSEIWDNMTWLQWDKEISNYTQIIYGLLEESQNQQEKNEQDLLALD
BG505_gp120_9MUTB         ----------------------------------------------------------------------------------------
BG505_gp120_10MUT_KO      ----------------------------------------------------------------------------------------
BG505_gp120_10MUT         ----------------------------------------------------------------------------------------
BG505_SOSIP.664_10MUT     QARVLAVERYLRDQQLLGIWGCSGKLICCTNVPWNSSWSNRNLSEIWDNMTWLQWDKEISNYTQIIYGLLEESQNQQEKNEQDLLALD
BG505_SOSIP_MD39_10MUTA   QARVLAVEHYLRDQQLLGIWGCSGKLICCTNVPWNSSWSNRNLSEIWDNMTWLQWDKEISNYTQIIYGLLEESQNQQEKNEQDLLALD
BG505_SOSIP.664_11MUTA    QARVLAVERYLRDQQLLGIWGCSGKLICCTNVPWNSSWSNRNLSEIWDNMTWLQWDKEISNYTQIIYGLLEESQNQQEKNEQDLLALD
BG505_SOSIP_MD39_11MUTB   QARVLAVEHYLRDQQLLGIWGCSGKLICCTNVPWNSSWSNRNLSEIWDNMTWLQWDKEISNYTQIIYGLLEESQNQQEKNEQDLLALD
```

Figure S4

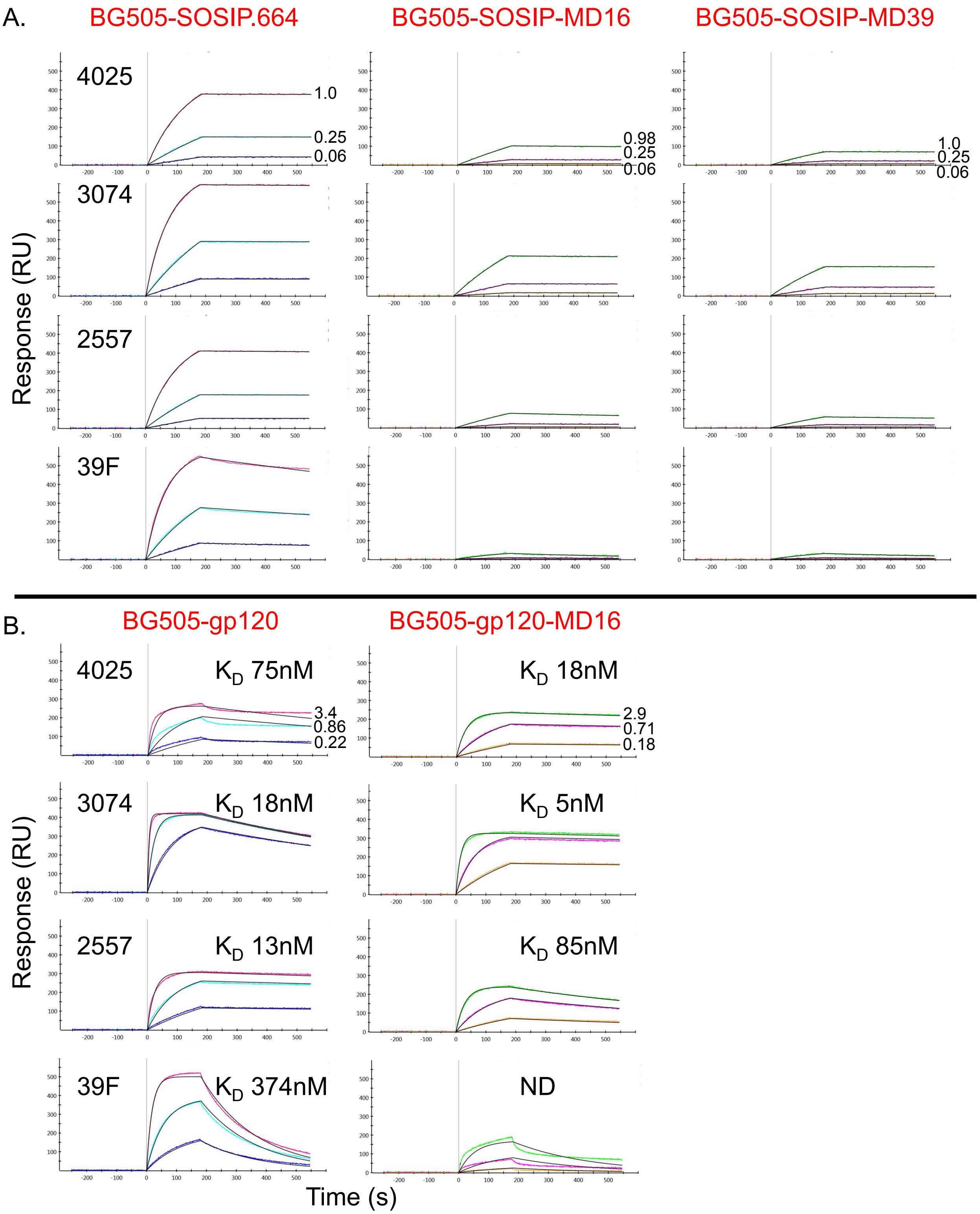

A.
BG505-SOSIP.664
BG505-SOSIP-MD16
BG505-SOSIP-MD39
4025
3074
2557
39F
1.0
0.25
0.06
0.98
0.25
0.06
1.0
0.25
0.06
Response (RU)
B.
BG505-gp120
BG505-gp120-MD16
4025
K_D 75nM
K_D 18nM
3.4
0.86
0.22
2.9
0.71
0.18
3074
K_D 18nM
K_D 5nM
2557
K_D 13nM
K_D 85nM
39F
K_D 374nM
ND
Response (RU)
Time (s)

Figure S5

A.

```
BG505_SOSIP.664    AENLWVTVYYGVPVWKDAETTLFCASDAKAYETEKHNVWATHACVPTDPNPQEIHLENVTEEFNMWKNNMVEQMHTDIIS
BG505_SOSIP_rare3  AENLWVTVYYGVPVWKDAETTLFCASDAKAYETEKHNVWATHACVPTDPNPQEIHLENVTEEFNMWKNNMVEQMHEDIIS
BG505_SOSIP_MD16   AENLWVTVYYGVPVWKDAETTLFCASDAKAYETEKHNVWATHACVPTDPNPQEIHLENVTEEFNMWKNNMVEQMHTDIIS
BG505_SOSIP_MD2    AENLWVTVYYGVPVWKDAETTLFCASDAKAYETEKHNVWATHACVPTDPNPQEIHLENVTEEFNMWKNNMVEQMHTDIIS
BG505_SOSIP_MD33   AENLWVTVYYGVPVWKDAETTLFCASDAKAYETEKHNVWATHACVPTDPNPQEIHLENVTEEFNMWKNNMVEQMHTDIIS
BG505_SOSIP_MD39   AENLWVTVYYGVPVWKDAETTLFCASDAKAYETEKHNVWATHACVPTDPNPQEIHLENVTEEFNMWKNNMVEQMHEDIIS

BG505_SOSIP.664    LWDQSLKPCVKLTPLCVTLQCTNVTNNITDDMRGELKNCSFNMTTELRDKKQKVYSLFYRLDVVQINENQGNRSNNSNKE
BG505_SOSIP_rare3  LWDQSLKPCVKLTPLCVTLQCTNVTNNITDDMRGELKNCSFNMTTELRDKKQKVYSLFYRLDVVQINENQGNRSNNSNKE
BG505_SOSIP_MD16   LWDQSLKPCVKLTPLCVTLQCTNVTNNITDDMRGELKNCSFNMTTELRDKKQKVYSLFYRLDVVQINENQGNRSNNSNKE
BG505_SOSIP_MD2    LWDQSLKPCVKLTPLCVTLQCTNVTNNITDDMRGELKNCSFNMTTELRDKKQKVYSLFYRLDVVQINENQGNRSNNSNKE
BG505_SOSIP_MD33   LWDQSLKPCVKLTPLCVTLQCTNVTNNITDDMRGELKNCSFNMTTELRDKKQKVYSLFYRLDVVQINENQGNRSNNSNKE
BG505_SOSIP_MD39   LWDQSLKPCVKLTPLCVTLQCTNVTNNITDDMRGELKNCSFNMTTELRDKKQKVYSLFYRLDVVQINENQGNRSNNSNKE

BG505_SOSIP.664    YRLINCNTSAITQACPKVSFEPIPIHYCAPAGFAILKCKDKKFNGTGPCPSVSTVQCTHGIKPVVSTQLLLNGSLAEEEV
BG505_SOSIP_rare3  YRLINCNTSAITQACPKVSFEPIPIHYCAPAGFAILKCKDKKFNGTGPCPSVSTVQCTHGIKPVVSTQLLLNGSLAEEEV
BG505_SOSIP_MD16   YRLINCNTSAITQACPKVSFEPIPIHYCAPAGWAILKCKDKKFNGTGPCPSVSTVQCTHGIKPVVSTQLLLNGSLAEEEV
BG505_SOSIP_MD2    YRLINCNTSAITQACPKVSFEPIPIHYCAPAGFAILKCKDKKFNGTGPCPSVSTVQCTHGIKPVVSTQLLLNGSLAEEEV
BG505_SOSIP_MD33   YRLINCNTSAITQACPKVSFEPIPIHYCAPAGFAILKCKDKKFNGTGPCPSVSTVQCTHGIKPVVSTQLLLNGSLAEEEV
BG505_SOSIP_MD39   YRLINCNTSAITQACPKVSFEPIPIHYCAPAGFAILKCKDKKFNGTGPCPSVSTVQCTHGIKPVVSTQLLLNGSLAEEEV

BG505_SOSIP.664    MIRSENITNNAKNILVQFNTPVQINCTRPNNNTRKSIRIGPGQAFYATGDIIGDIRQAHCNVSKATWNETLGKVVKQLRK
BG505_SOSIP_rare3  IIRSENITNNAKNILVQLNAPVQINCTRPNNNTRKSIRIGPGQAFYATGDIIGDIRQAHCNVSKATWNETLGKVVKQLRK
BG505_SOSIP_MD16   MIRSENITNNAKNILVQFNTPVQINCTRPNNNTVKSIRIGPGQAFYYTGDIIGDIRQAHCNVSKATWNETLGKVVKQLRK
BG505_SOSIP_MD2    MIRSENITNNAKNILVQFNTPVQINCTRPNNNTRKSIRIGPGQAFYATGDIIGDIRQAHCNVSKATWNETLGKVVKQLRK
BG505_SOSIP_MD33   MIRSENITNNAKNILVQFNTPVQINCTRPNNNTRKSIRIGPGQAFYATGDIIGDIRQAHCNVSKATWNETLGKVVKQLRK
BG505_SOSIP_MD39   IIRSENITNNAKNILVQLNTPVQINCTRPNNNTVKSIRIGPGQAFYYTGDIIGDIRQAHCNVSKATWNETLGKVVKQLRK

BG505_SOSIP.664    HFGNNTIIRFANSSGGDLEVTTHSFNCGGEFFYCNTSGLFNSTWISNTSVQGSNSTGSNDSITLPCRIKQIINMWQRIGQ
BG505_SOSIP_rare3  HFGNNTIIRFAQSSGGDLEVTTHSFNCGGEFFYCNTSGLFNSTWISNTSVQGSNSTGSNDSITLPCRIKQIINMWQRIGQ
BG505_SOSIP_MD16   HFGNNTIIRFANSSGGDLEVTTHSFNCGGEFFYCNTSGLFNSTWISNTSVQGSNSTGSNDSITLPCRIKQIINMWQRIGQ
BG505_SOSIP_MD2    HFGNNTIIRFANSSGGDLEVTTHSFNCGGEFFYCNTSGLFNSTWISNTSVQGSNSTGSNDSITLPCRIKQIINMWQRIGQ
BG505_SOSIP_MD33   HFGNNTIIRFANSSGGDLEVTTHSFNCGGEFFYCNTSGLFNSTWISNTSVQGSNSTGSNDSITLPCRIKQIINMWQRIGQ
BG505_SOSIP_MD39   HFGNNTIIRFAQSSGGDLEVTTHSFNCGGEFFYCNTSGLFNSTWISNTSVQGSNSTGSNDSITLPCRIKQIINMWQRIGQ

BG505_SOSIP.664    AMYAPPIQGVIRCVSNITGLILTRDGGSTNSTTETFRPGGGDMRDNWRSELYKYKVVKIEPLGVAPTRCKRRVVGRRRRR
BG505_SOSIP_rare3  AMYAPPIQGVIRCVSNITGLILTRDGGSTNSTTETFRPGGGDMRDNWRSELYKYKVVKIEPLGVAPTRCKRRVVGRRRRR
BG505_SOSIP_MD16   AMYAPPIQGVIRCVSNITGLILTRDGGSTNSTTETFRPGGGDMRDNWRSELYKYKVVKIEPLGVAPTRCKRRVVGRRRRR
BG505_SOSIP_MD2    AMYAPPIQGVIRCVSNITGLILTRDGGSTNSTTETFRPGGGDMRDNWRSELYKYKVVKIEPLGVAPTRCKRRVVGRRRRR
BG505_SOSIP_MD33   AMYAPPIQGVIRCVSNITGLILTRDGGSTNSTTETFRPGGGDMRDNWRSELYKYKVVKIEPLGVAPTRCKRRVVGRRRRR
BG505_SOSIP_MD39   AMYAPPIQGVIRCVSNITGLILTRDGGSTNSTTETFRPGGGDMRDNWRSELYKYKVVKIEPLGVAPTRCKRRVVGRRRRR

BG505_SOSIP.664    RAVGIGAVFLGFLGAAGSTMGAASMTLTVQARNLLSGIVQQQSNLLRAPEAQQHLLKLTVWGIKQLQARVLAVERYLRDQ
BG505_SOSIP_rare3  RAVGIGAVFLGFLGAAGSTMGAASMTLTVQARNLLSGIVQQQSNLLRAPEAQQHLLKLTVWGIKQLQARVLAVERYLRDQ
BG505_SOSIP_MD16   RAVGIGAVFLGFLGAAGSTMGAASMTLTVQARNLLSGIVQQQSNLLRAPEAQQHLLKDTVWGIKQLQARVLAVERYLRDQ
BG505_SOSIP_MD2    RAVGIGAVFLGFLGAAGSTMGAASMTLTVQARNLLSGIVQQQSNLLRAPEAQQHLLKDTVWGIKQLQARVLAVERYLRDQ
BG505_SOSIP_MD33   RAVGIGAVSLGFLGAAGSTMGAASMTLTVQARNLLSGIVQQQSNLLRAPEPQQHLLKDTHWGIKQLQARVLAVEHYLRDQ
BG505_SOSIP_MD39   RAVGIGAVSLGFLGAAGSTMGAASMTLTVQARNLLSGIVQQQSNLLRAPEPQQHLLKDTHWGIKQLQARVLAVEHYLRDQ

BG505_SOSIP.664    QLLGIWGCSGKLICCTNVPWNSSWSNRNLSEIWDNMTWLQWDKEISNYTQIIYGLLEESQNQQEKNEQDLLALD
BG505_SOSIP_rare3  QLLGIWGCSGKLICCTNVPWNSSWSNRNLSEIWDNMTWLQWDKEISNYTQIIYGLLEESQNQQEKNEQDLLALD
BG505_SOSIP_MD16   QLLGIWGCSGKLICCTNVPWNSSWSNRNLSEIWDNMTWLQWDKEISNYTQIIYGLLEESQNQQEKNEQDLLALD
BG505_SOSIP_MD2    QLLGIWGCSGKLICCTNVPWNSSWSNRNLSEIWDNMTWLQWDKEISNYTQIIYGLLEESQNQQEKNEQDLLALD
BG505_SOSIP_MD33   QLLGIWGCSGKLICCTNVPWNSSWSNRNLSEIWDNMTWLQWDKEISNYTQIIYGLLEESQNQQEKNEQDLLALD
BG505_SOSIP_MD39   QLLGIWGCSGKLICCTNVPWNSSWSNRNLSEIWDNMTWLQWDKEISNYTQIIYGLLEESQNQQEKNEQDLLALD
```

B.

Trimer	Tm (°C)
BG505 SOSIP	66.65
rare3	68.08
MD16	66.5
MD2	66.99
MD33	70.65
MD39	77.17

C.

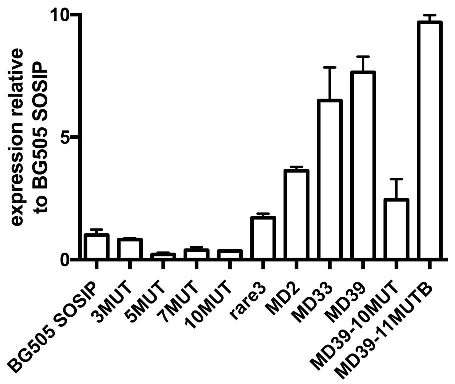

Figure S6

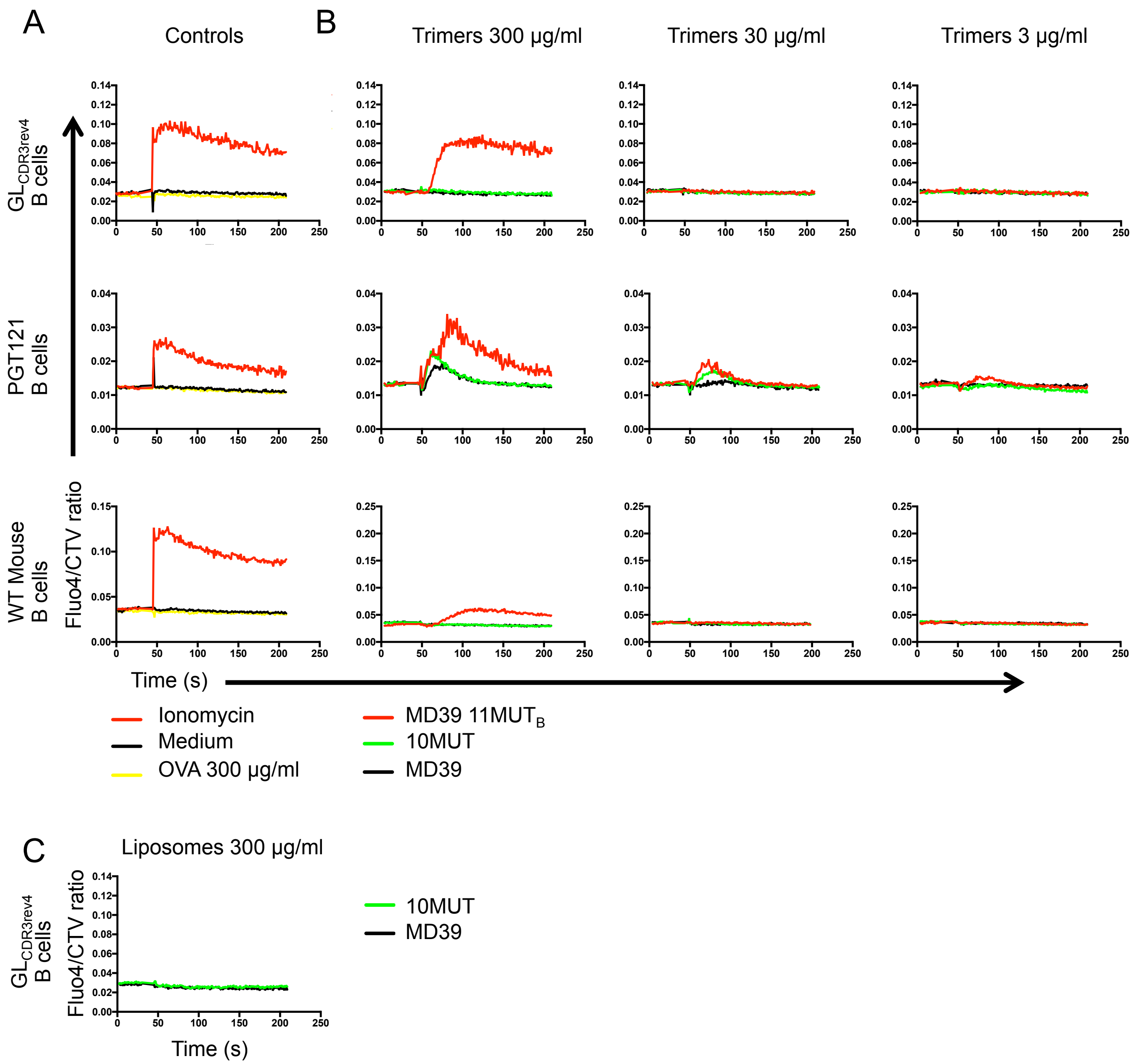
A
Controls
B
Trimers 300 µg/ml
Trimers 30 µg/ml
Trimers 3 µg/ml
GL CDR3rev4 B cells
PGT121 B cells
WT Mouse B cells
Fluo4/CTV ratio
Time (s)
Ionomycin
Medium
OVA 300 µg/ml
MD39 11MUT B
10MUT
MD39
C
Liposomes 300 µg/ml
GL CDR3rev4 B cells
Fluo4/CTV ratio
10MUT
MD39
Time (s)

Figure S7

A

	Dissociation constant K_d (nM)							
	VRC01	121	128	151	145	1400	Tm (°C)	Yield PEI
BG505	131	22	8	10	6	2	66	1
MD39-VLC1	416	42	5	10	31	-	73	13.7
MD39-VLC2	124	21	12	11	20	12	77	6.2
MD39-VLC3	449	15	7	11	33	-	77	7.0
MD39-VLC4	167	13	2	11	27	-	76	6.3
MD39-VLC5	121	50	7	10	11	-	74	10.5

B

```
BG505_SOSIP_D664_MD39     AENLWVTVYYGVPVWKDAETTLFCASDAKAYETEKHNVWATHACVPTDPNPQEIHLENVTEEFNMWKNNMVE
BG505_SOSIP_MD39_VLC1-03 AENLWVTVYYGVPVWKDAETTLFCASDAKAYETEKHNVWATHACVPTDPNPQEIHLENVTEEFNMWKNNMVE
BG505_SOSIP_MD39_VLC2-04 AENLWVTVYYGVPVWKDAETTLFCASDAKAYETEKHNVWATHACVPTDPNPQEIHLENVTEEFNMWKNNMVE
BG505_SOSIP_MD39_VLC2-08 AENLWVTVYYGVPVWKDAETTLFCASDAKAYETEKHNVWATHACVPTDPNPQEIHLENVTEEFNMWKNNMVE
BG505_SOSIP_MD39_VLC3-13 AENLWVTVYYGVPVWKDAETTLFCASDAKAYETEKHNVWATHACVPTDPNPQEIHLENVTEEFNMWKNNMVE

BG505_SOSIP_D664_MD39     QMHEDIISLWDQSLKPCVKLTPLCVTLQC------TNVTNNITDD----MRGELKNCSFNMTTELRDKKQKV
BG505_SOSIP_MD39_VLC1-03 QMHEDIISLWDQSLKPCVKLTPLCVTLQC------TDWDNATLAN----MTGEIKNCSFNMTTELRDKKQKV
BG505_SOSIP_MD39_VLC2-04 QMHEDIISLWDQSLKPCVKLTPLCVTLQCSDYEGNTTRQNITMKE----EKGEIKNCSFNMTTELRDKKQKV
BG505_SOSIP_MD39_VLC2-08 QMHEDIISLWDQSLKPCVKLTPLCVTLQCVTLKNCSNSNCSISRNISIEMDGEIKNCSFNMTTELRDKKQKV
BG505_SOSIP_MD39_VLC3-13 QMHEDIISLWDQSLKPCVKLTPLCVTLQCTNAAL-TNVTITNGPN----ITEEIRNCSFNMTTELRDKKQKV

BG505_SOSIP_D664_MD39     YSLFYRLDVVQINEN-----------QGNRSNNSNKEYRLINCNTSAITQACPKVSFEPIPIHYCAPAGFAI
BG505_SOSIP_MD39_VLC1-03 YSLFYELDIIPIENE-----------YISNNNTSNTSYRLINCNTSAITQACPKVSFEPIPIHYCAPAGFAI
BG505_SOSIP_MD39_VLC2-04 YSLFYKLDITPIEEDNNS--NNSSSANSSNSNANYTNYRLINCNTSAITQACPKVSFEPIPIHYCAPAGFAI
BG505_SOSIP_MD39_VLC2-08 YSLFYRLDIVPIESSNNSQLSNNSQVSNNSQSSNYSQYRLINCNTSAITQACPKVSFEPIPIHYCAPAGFAI
BG505_SOSIP_MD39_VLC3-13 YSLFYKLDLVQINGS-------------------GGEYRLINCNTSAITQACPKVSFEPIPIHYCAPAGFAI

BG505_SOSIP_D664_MD39     LKCKDKKFNGTGPCPSVSTVQCTHGIKPVVSTQLLLNGSLAEEEVIIRSENITNNAKNILVQLNTPVQINCT
BG505_SOSIP_MD39_VLC1-03 LKCKDKKFNGTGPCPSVSTVQCTHGIKPVVSTQLLLNGSLAEEEVIIRSENITNNAKNILVQLNTPVQINCT
BG505_SOSIP_MD39_VLC2-04 LKCKDKKFNGTGPCPSVSTVQCTHGIKPVVSTQLLLNGSLAEEEVIIRSENITNNAKNILVQLNTPVQINCT
BG505_SOSIP_MD39_VLC2-08 LKCKDKKFNGTGPCPSVSTVQCTHGIKPVVSTQLLLNGSLAEEEVIIRSENITNNAKNILVQLNTPVQINCT
BG505_SOSIP_MD39_VLC3-13 LKCKDKKFNGTGPCPSVSTVQCTHGIKPVVSTQLLLNGSLAEEEVIIRSENITNNAKNILVQLNTPVQINCT

BG505_SOSIP_D664_MD39     RPNNNTVKSIRIGPGQAFYYTGDIIGDIRQAHCNVSKATWNETLGKVVKQLRKHFGNNTIIRFAQSSGGDLE
BG505_SOSIP_MD39_VLC1-03 RPNNNTVKSIRIGPGQAFYYTGDIIGDIRQAHCNVSATQWEQTLKGIAAKLLEHFGNNTIIRFAQSSGGDLE
BG505_SOSIP_MD39_VLC2-04 RPNNNTVKSIRIGPGQAFYYTGDIIGDIRQAHCNVSGTKWKNTLKQIVKKLGDHFGNNTIIRFAQSSGGDLE
BG505_SOSIP_MD39_VLC2-08 RPNNNTVKSIRIGPGQAFYYTGDIIGDIRQAHCNVSKKDWEKTLQQVATKLGQHFGNNTIIRFAQSSGGDLE
BG505_SOSIP_MD39_VLC3-13 RPNNNTVKSIRIGPGQAFYYTGDIIGDIRQAHCNVSGTKWNETLKQVAGKLRKHFGNNTIIRFAQSSGGDLE

BG505_SOSIP_D664_MD39     VTTHSFNCGGEFFYCNTSGLFNSTWISNT-----SVQGSNSTGSNDSITLPCRIKQIINMWQRIGQAMYAPP
BG505_SOSIP_MD39_VLC1-03 VTTHSFNCGGEFFYCNTSGLFNGSSWNLN-----KTKENTTNLENGTITLPCRIKQIINMWQRIGQAMYAPP
BG505_SOSIP_MD39_VLC2-04 VTTHSFNCGGEFFYCNTSGLFNSTWNRTNGTWN-DVEGLNYTNGNDTITLPCRIKQIINMWQRIGQAMYAPP
BG505_SOSIP_MD39_VLC2-08 VTTHSFNCGGEFFYCNTSGLFNSTWIRNSSNSTWNSSASNSTELNSNITLPCRIKQIINMWQRIGQAMYAPP
BG505_SOSIP_MD39_VLC3-13 VTTHSFNCGGEFFYCNTSGLFNSTWPENG-----TMEGS-----NGTITLPCRIKQIINMWQRIGQAMYAPP

BG505_SOSIP_D664_MD39     IQGVIRCVSNITGLILTRDGGSTN-STTETFRPGGGDMRDNWRSELYKYKVVKIEPLGVAPTRCKRRVVGRR
BG505_SOSIP_MD39_VLC1-03 IQGVIRCVSNITGLILTRDGGNKS-AGIETFRPGGGDMRDNWRSELYKYKVVKIEPLGVAPTRCKRRVVGRR
BG505_SOSIP_MD39_VLC2-04 IQGVIRCVSNITGLILTRDGGNDT-DKNETFRPGGGDMRDNWRSELYKYKVVKIEPLGVAPTRCKRRVVGRR
BG505_SOSIP_MD39_VLC2-08 IQGVIRCVSNITGLILTRDGGHETENKTETFRPGGGDMRDNWRSELYKYKVVKIEPLGVAPTRCKRRVVGRR
BG505_SOSIP_MD39_VLC3-13 IQGVIRCVSNITGLILTRDGGNST-TDTETFRPGGGDMRDNWRSELYKYKVVKIEPLGVAPTRCKRRVVGRR

BG505_SOSIP_D664_MD39     RRRRAVGIGAVSLGFLGAAGSTMGAASMTLTVQARNLLSGIVQQQSNLLRAPEPQQHLLKDTHWGIKQLQAR
BG505_SOSIP_MD39_VLC1-03 RRRRAVGIGAVSLGFLGAAGSTMGAASMTLTVQARNLLSGIVQQQSNLLRAPEPQQHLLKDTHWGIKQLQAR
BG505_SOSIP_MD39_VLC2-04 RRRRAVGIGAVSLGFLGAAGSTMGAASMTLTVQARNLLSGIVQQQSNLLRAPEPQQHLLKDTHWGIKQLQAR
BG505_SOSIP_MD39_VLC2-08 RRRRAVGIGAVSLGFLGAAGSTMGAASMTLTVQARNLLSGIVQQQSNLLRAPEPQQHLLKDTHWGIKQLQAR
BG505_SOSIP_MD39_VLC3-13 RRRRAVGIGAVSLGFLGAAGSTMGAASMTLTVQARNLLSGIVQQQSNLLRAPEPQQHLLKDTHWGIKQLQAR

BG505_SOSIP_D664_MD39     VLAVEHYLRDQQLLGIWGCSGKLICCTNVPWNSSWSNRNLSEIWDNMTWLQWDKEISNYTQIIYGLLEESQN
BG505_SOSIP_MD39_VLC1-03 VLAVEHYLRDQQLLGIWGCSGKLICCTNVPWNSSWSNRNLSEIWDNMTWLQWDKEISNYTQIIYGLLEESQN
BG505_SOSIP_MD39_VLC2-04 VLAVEHYLRDQQLLGIWGCSGKLICCTNVPWNSSWSNRNLSEIWDNMTWLQWDKEISNYTQIIYGLLEESQN
BG505_SOSIP_MD39_VLC2-08 VLAVEHYLRDQQLLGIWGCSGKLICCTNVPWNSSWSNRNLSEIWDNMTWLQWDKEISNYTQIIYGLLEESQN
BG505_SOSIP_MD39_VLC3-13 VLAVEHYLRDQQLLGIWGCSGKLICCTNVPWNSSWSNRNLSEIWDNMTWLQWDKEISNYTQIIYGLLEESQN

BG505_SOSIP_D664_MD39     QQEKNEQDLLALD
BG505_SOSIP_MD39_VLC1-03 QQEKNEQDLLALD
BG505_SOSIP_MD39_VLC2-04 QQEKNEQDLLALD
BG505_SOSIP_MD39_VLC2-08 QQEKNEQDLLALD
BG505_SOSIP_MD39_VLC3-13 QQEKNEQDLLALD
```

C

Position	N332-epitope Diversity
156	N(97%)
158	S(89%), T(10%)
295	N(66%), V(13%)
297	T(81%), I(12%)
299	P(98%)
301	N(95%)
303	T(94%)
320	T(90%)
321	G(79%)
322	D(49%), E(22%)
323	I(85%)
324	G(99%)
325	D(80%), N(16%)
326	I(96%)
327	R(98%)
328	Q(65%), K(24%)
330	H(66%), Y(31%)
332	N(72%), E(12%)
334	S(67%), N(29%)
386	N(90%)
388	T(48%), S(48%)
389	Q(26%), K(25%), G(19%)
392	N(92%)
394	T(79%)
415	T(71%), I(22%)
417	P(79%), Q(16%)
419	R(81%), K(18%)
442	N(27%), Q(22%)
444	T(29%), R(29%), N(20%)

Supplemental Figure Legends

Figure S1. Mammalian display overview related to Figure 1.
(A) Schematic of mammalian display procedure.
(B) Example FACS plots for unsorted mammalian display library (top) and the same library sorted 3 times (bottom), see Extended Experimental Procedures.
(C) Sequences of BG505-SOSIP and BG505-gp120 used for mammalian display. Leader peptides are shown in red, the cMyc epitope is shown in green and the PDGFR TM is shown in blue.

Figure S2. Germline-reverted PGT121 Abs, related to Figure 1. Sequences of PGT121 and germline-reverted variants with mutations highlighted relative to germline V4-59/D3-3/J6 (heavy chain) and V3-21/J3 (light chain) genes.

Figure S3. PGT121 germline targeting gp120s and gp140s, related to Figure 1. Designed germline targeting trimer and gp120 sequences are shown with mutations from BG505-SOSIP highlighted. BG505-SOSIP.D664 contains the mutation T332N, not highlighted.

Figure S4. SPR binding data for V3 Abs binding to gp140 SOSIPs and their matching gp120s, related to Figure 2.
(A) Comparison of V3 binding between WT BG505-SOSIP and BG505-SOSIP variants containing R304V and A319Y V3 mutations. For the 4025 SPR plots, the gp140 concentrations tested are shown in μM next to each relevant sensogram and are equivalent for all V3 Abs tested.
(B) Comparison of V3 binding to WT gp120 and gp120-MD16 containing the R304V and A319Y mutations. For the 4025 SPR plots, the gp120 concentrations tested are shown in μM next to each relevant sensogram and are equivalent for all V3 Abs tested.

Figure S5. Trimers with improved thermostability, expression, or antigenic profile, related to Figure 2.
(A) Sequences of designed stabilized native-like trimers with mutations from BG505 SOSIP.D664 highlighted.
(B) Melting temperature of stabilized trimers as assessed by DSC.
(C) Native-like trimers with and without stabilizing mutations were transiently transfected in 293F cells and expression levels were determined by capture ELISA using PGT145 Fab for immobilization and PGT151 IgG for detection. Values are the mean ± SD of 3 replicate transfections.

Figure S6. Ex vivo B cell activation assay, related to Figure 5.
Ca^{2+} flux transients detected as increases in Fluo-4 fluorescence after addition of (A) control activators (Ionomycin and IgM positive controls, Ovalbumin negative control), (B) trimer (MD39, 10MUT, MD39-11MUT$_{B}$) at the indicated concentrations, or (C) trimer-liposomes for 10MUT or MD39 at 300 μg/mL. In (A) and (B), data are shown for germline-reverted PGT121 (GL$_{CDR3rev4}$) B cells (top), mature PGT121 B cells (middle), and WT mouse B cells (bottom).

Figure S7. Sequences and biophysical properties of the MD39-based VLC native-like trimer cocktail, and N332-epitope sequence diversity, Related to Figure 7.
A) Biophysical characterization of the MD39-based VLC cocktail.
(B) Sequences of MD39-based VLC cocktail members, with changes relative to MD39 highlighted in green.
(C) List of interface positions on the BG505 SOSIP trimer near N332-supersite bnAb epitopes (PGT122, PGT128, PGT135), showing the frequencies of the amino acids found at those positions in 10% or more of 3,897 unique HIV Env sequences isolated from infected individuals obtained from www.hiv.lanl.gov.

Table S1. The binding affinities of germline targeting gp120s, related to Figure 1.

BG505-gp120	PGT121	3H3L	GL+9	GL+3	$GL_{CDR3\text{-}mat}$	$GL_{CDR3\text{-}rev5}$	$GL_{CDR3\text{-}rev4}$	$GL_{CDR3\text{-}rev3}$	$GL_{CDR3\text{-}rev2}$	$GL_{CDR3\text{-}rev1}$	$GL_{H\text{-}rev4}$ 121_L	121_H $GL_{L\text{-}rev4}$
WT (T332N)	7.5	250	28000	>128000	>128000	>8000	>84000	>128000	-	>8000	600	>38000
2MUT	2.7	-	4900	>40000	-	-	>40000	-	-	-	63	>40000
3MUT	4.6	19	1600	>28000	>21000	-	>28000	>11000	-	-	22	>28000
5MUT	5.7	2.5	18	WB	WB	-	>34000	-	-	-	6	13000
6MUT	1.4	-	19	>24000	-	-	>24000	-	-	-	5	>24000
7MUT	1.2	0.25	3	12200	44000	-	>36000	-	-	-	1.3	>36000
$9MUT_A$	0.57	-	-	2700	2900	-	>70000	-	-	-	-	57000
$9MUT_B$	1.5	28	-	29000	WB	-	>107000	-	-	-	220	39000
10MUT	0.59	0.04	1.2	1200	790	WB	>150000	WB	>150000	WB	-	47000
10MUT-KO	435	-	-	-	>21000	-	>21000	-	-	-	20000	>21000
$11MUT_A$	-	-	-	-	1200	-	WB	-	-	-	-	51000
$11MUT_B$	0.15	0.075	0.6	600	840	7700	WB	3000	-	5200	-	-

Values are K_Ds (nM) measured by SPR.
WB, weak binding, not quantified.
-, not measured.

Table S2. Data collection and refinement statistics, related to Figure 3.

Beamline	APS 23-ID-D
Wavelength (Å)	1.03322
Resolution (Å)[a]	49.6 – 4.5 (4.58 – 4.5)
Space group	$P6_3$
Unit cell (Å, °)	127.92, 127.92, 313.89 90, 90, 120
Total reflections	73,804 (3970)
Unique reflections	17,067 (887)
Multiplicity	4.3 (4.4)
Completeness (%)	98.4 (98.9)
Mean (I/σ_I)	4.75 (1.1)
R_{merge}[b]	0.213 (1.00)
R_{meas}[c]	0.207 (1.00)
R_{pim}[d]	0.175 (1.00)
$CC_{1/2}$[e]	0.76 (0.63)
R_{work}	0.272 (0.382)
R_{free}	0.309 (0.372)
# reflections used in refinement (work/free)	16142/844
# Protein atoms	11183
# Carbohydrate atoms	653
# Waters	0
# Protein residues	1452
RMS (bonds)	0.012
RMS (angles)	1.55
Ramachandran favored, outliers (%)	96.2, 0.3
Clashscore[f]	7.8
Wilson B ($Å^2$)	105.6
Average B ($Å^2$)	186.0

[a]Numbers in parentheses are for highest resolution shell

[b]$R_{merge} = \Sigma_{hkl} \Sigma_{i=1,n}|I_i(hkl) - \langle I(hkl)\rangle| / \Sigma_{hkl} \Sigma_{i=1,n} I_i(hkl)$

[c]$R_{meas} = \Sigma_{hkl} \sqrt{(n/n-1)} \Sigma_{i=1,n}|I_i(hkl) - \langle I(hkl)\rangle| / \Sigma_{hkl} \Sigma_{i=1,n} I_i(hkl)$

[d]$R_{pim} = \Sigma_{hkl} \sqrt{(1/n-1)} \Sigma_{i=1,n}|I_i(hkl) - \langle I(hkl)\rangle| / \Sigma_{hkl} \Sigma_{i=1,n} I_i(hkl)$

[e]$CC_{1/2}$ = Pearson Correlation Coefficient between two random half datasets

[f]Number of unfavorable all-atom steric overlaps ≥ 0.4Å per 1000 atoms

Table S3. Sequential boosting pairs that were eliminated based on violation of directionality, related to Figure 7.

Sequential boosting pair	Directionality violation
11B ➔ 10/9A	11B contains the native residue N137 which is mutated to F in 10/9A
6 ➔ 3	6 contains the native glycosylation site at N133 which is mutated in 3
5 ➔ 3	5 contains native glycosylation sites at N133 and N137 and both are mutated in 3
5 ➔ 2	5 contains native glycosylation site at N137 which is mutated in 2

Any boosting pair in which the first immunogen contains a native residue that is mutated in the second immunogen is a violation of directionality. The immunogen names have "MUT" removed for simplicity.

Table S4. Characteristics of sequential boosting pairs that obey directionality, related to Figure 7.

Sequential boosting pair	Affinity drop#	# of AA changes	# of AA closer to WT	comment
11B/10 ➔ 7	Small (5/3)	6/3	4/3	Shown in Figure 7.
11B/10 ➔ 6	Medium (32/16)	7/4	5/4	Shown in Figure 7.
11B/10 ➔ 5	Medium (30/15)	7/5	6/5	Shown in Figure 7.
11B/10 ➔ 3	Large (2700/1300)	9/7	8/7	Shown in Figure 7.
11B/10 ➔ WT	Large (47000/23000)	10/9	10/9	Shown in Figure 7.
7 ➔ 5	Small (6)	2	2	Shown in Figure 7.
7 ➔ 3	Medium (530)	4	4	Shown in Figure 7.
7 ➔ WT	Large (9300)	6	6	Shown in Figure 7.
6 ➔ WT	Large (1500)	5	5	Shown in Figure 7.
5 ➔ WT	Large (1600)	4	4	Shown in Figure 7.
3 ➔ WT	Medium (18)	2	2	Shown in Figure 7.
11B/10 ➔ 2	Large (8200/4100)	10/8	9/8	Would be followed by: 2 ➔ WT
10 ➔ 9A	Small (4)*	1	1	Small affinity drop and only 1 mutation, thus inefficient
7 ➔ 6	Small (6)	1	1	Small affinity drop and only 1 mutation, thus inefficient
6 ➔ 5	Small (1)	1	1	Small affinity drop and only 1 mutation, thus inefficient
6 ➔ 2	Medium (260)	4	4	Would be followed by: 2 ➔ WT
3 ➔ 2	Small (3)	1	1	Small affinity drop and only 1 mutation, thus inefficient
2 ➔ WT	Small (6)	1	1	Small affinity drop and only 1 mutation, thus inefficient

#, Affinity drops were calculated based on binding to the GL+9 antibody, as described in the text, except where noted otherwise. Affinity drops were defined as small (<10), medium (10-1000), or large (>1000).
*, Affinity drops were calculated based on binding to the $GL_{CDR3\text{-}mat}$ antibody.
The immunogen names have "MUT" removed for simplicity.
WT, BG505-T332N.

Supplemental Experimental Procedures

DNA gene synthesis. Genes were synthesized at Genscript, Inc. Gp120 and gp140 variants in pHLsec contained a C-terminal GTKHHHHHH tag. Genes in pENTR contained a C-terminal cMyc epitope followed by a PDGFR transmembrane domain. IgGs were cloned into pFUSEss and Fabs were in a modified version of pFUSEss (pFABss). DNA was maxi-prepped using a BenchPro 2100.

Protein production. BG505-gp120 and variants based on BG505 contained the L111A mutation for more efficient production of monomer compared to other species (Hoffenberg et al., 2013) and the T332N mutation and were expressed in 293F cells grown in 293 Freestyle media (Life Technologies) by transient transfection with 293Fectin (Invitrogen). Protein was harvested from the supernatant 96 h post transfection and purified by nickel affinity chromatography on a HIS-TRAP column (GE) followed by HiLoad 16/600 Superdex 200 size exclusion chromatography (GE Healthcare). Gp140 SOSIPs were expressed in 293F cells grown in 293 Freestyle media by transient transfection with either 293Fectin or PEI. The protein was purified from the supernatant using a HIS-TRAP column, starting with a wash buffer (20 mM Imidazole, 500 mM NaCl, 20 mM Na_2HPO_4) and mixing with elution buffer (500 mM Imidazole, 500 mM NaCl, 20 mM Na_2HPO_4) using a linear gradient. The trimer fraction was collected and further purified on an S200Increase 10-300 column (GE) in HBS (10 mM HEPES, 150 mM NaCl). The oligomeric state of the SOSIP trimers were then confirmed by size exclusion chromatography-multi-angle light scattering (SECMALS) using the DAWN HELEOS II multi-angle light scattering system with Optilab T-rEX refractometer (Wyatt Technology). The trimers were frozen in thin-walled PCR tubes at 1 mg/ml using liquid nitrogen and stored at -80°C (Jardine et al., 2015). Fabs and mAbs were produced in 293F cells as described previously (Jardine et al., 2013). For crystallography, SOSIP_MD39_10MUTA was expressed in 293S cells.

ELISA quanitification of SOSIP expression. BG505 SOSIP variants were expressed using the Freestyle 293F expression system (Thermo Scientific) according to manufacturer's instructions. After 4 days, supernatants were harvested by centrifugation and stored at 4˚C until analysis. Capture ELISAs were performed essentially as described previously (Schiffner et al., 2016). Briefly, ELISA plates were coated overnight with trimer specific PGT145 Fab at 4 μg/mL in PBS at 4˚C followed by blocking with 2% w/v bovine serum albumin (BSA) in washing buffer (PBS + 0.05% v/v tween20). SOSIP expression supernatants were diluted 100x in sample buffer (washing buffer + 1% w/v BSA) and for each variant, a standard curve with known concentration of matching purified protein was prepared in sample buffer. Supernatants and standard curves were added to ELISA plates and detected with trimer preferring IgG PGT151 at 10 μg/mL in sample buffer. Samples were labeled with horseradish peroxidase coupled Fcg-specific anti-human IgG (Jackson Immunoresearch), developed and stopped with 1-Step Ultra TMB-ELISA substrate (Thermo Scientific) as per manufacturer's instructions, and optical densities were read at 450 nm and 570 nm. After background subtraction, data were fit to a "one-site specific binding with hill slope" curve in graphpad prism, and supernatant concentrations were extrapolated from standard curves.

Surface plasmon resonance (SPR). Kinetics and affinities of antibody-antigen interactions were measured on a ProteOn XPR36 (Bio-Rad) using GLC Sensor Chip (Bio-Rad) and 1x HBS-EP+ pH 7.4 running buffer (20x stock from Teknova, Cat. No H8022) supplemented with BSA at 1mg/ml. Human Antibody Capture Kit was used according to manufacturer's instructions (Cat. No BR-1008-39 from GE) to immobilize about 6000 RUs of capture mAb onto each flow cell. In a typical experiment, approximately 300-400 RUs of mAbs were captured onto each flow cell and analytes were passed over the flow cell at 50 μL/min for 3 min followed by a 5 min dissociation time. Regeneration was accomplished using 3M Magnesium Chloride with 180 seconds contact time and injected four times per cycle. Raw sensograms were analyzed using ProteOn Manager software (Bio-Rad), including interspot and column double referencing, and either Equilibrium fits or Kinetic fits with Langmuir model, or both, were employed when applicable. Analyte concentrations were measured on a NanoDrop 2000c Spectrophotometer using Absorption signal at 280 nm (Jardine et al., 2015). We measured kinetics and affinity of antibody-Fab-fragment antigen interactions on ProteOn XPR36 (Bio-Rad) using HTE Sensor Chip (Bio-Rad) and running buffer with 20 mM Sodium Phosphate Dibasic, pH 7.4, 500 mM Sodium Chloride, 50 mM Imidazole, supplemented with BSA at 1mg/ml and Tween 20 detergent at 0.05% v/v. We used 0.1 M Nickel sulfate as activation solution. 0.5 M EDTA was our regeneration solution with 300 seconds contact time and injected two times per cycle (one

time each for vertical and horizontal orientation). Raw sensograms were analyzed using ProteOn Manager software (Bio-Rad), interspot and column double referencing, Equilibrium or Kinetic with Langmuir model or both where applicable. Analyte concentrations were measured on NanoDrop 2000c Spectrophotometer using Absorption signal at 280 nm.

Design of PGT121 germline-targeting immunogens.
BG505-gp120 T332N fused to the PDGFR transmembrane domain (TM) was subjected to random mutagenesis using error prone PCR (gene morph II Agilent), and the resulting PCR product was gel purified and ligated into a modified version of the gateway cloning entry vector pENTR/D-TOPO (Ota et al., 2012) using the circular polymerase extension cloning (CPEC) method (Quan and Tian, 2014). The ligated vector containing the error prone library was purified using the PCR purification kit (Qiagen) and concentrated. The concentrated library was then transformed into electroMAX DH5a-E competent cells (Invitrogen) and grown overnight at 37°C in a 125 mL culture. The plasmid was purified using the BenchPro® 2100 (Invitrogen) and the gp120 insert was transferred to the lentiviral vector pLenti CMVTRE3G puro Dest (Ota et al., 2012) using the LR Clonase II enzyme mix (Invitrogen). The LR clonase reaction was scaled up ~10-fold to increase library size. The LR clonase product was again purified, concentrated and transformed into electroMAX stbl4 competent cells (Invitrogen) and grown overnight at 30°C in a 125 mL culture. This plasmid DNA was purified and ready for use in transfection. 293T cells cultured in Advanced DMEM (Gibco) supplemented with 5% FCS, GlutaMAX (Gibco), 2-mercaptoethanol (Gibco) and Antibiotic-Antimycotic (Gibco) were co-transfected with the BG505-gp120 error prone PCR library in pLenti CMVTRE3G puro Dest (10.8 μg), psPAX2 (7.0 μg) and pMD2.G (3.8 μg) with fugeneHD in a T75 flask (Salmon and Trono, 2007). The cells were kept at 37°C for two days and then the media containing the virus was collected and spun down at 500g for 5min. 293T cells stably expressing rtTA3G from the pLenti CMV rtTA3G Blast vector (obtained from Dave Nemazee; (Ota et al., 2012)) were transduced at low moi (<0.1) in a T75 or T225 flask in the presence of 10 μg/mL blasticidin. The next day cells were selected with 2 μg/mL puromycin. 293T cells containing the stable library were induced with doxycycline (1μg/mL) and the following day were harvested in FACS buffer (HBSS, 1 mM EDTA, 0.5% BSA). Cells were stained with either the GL+9 or GL+3 Ab for ~30 min, washed with FACS buffer, and then stained with fluorescein isothiocyanate (FITC)-labeled α-cMyc (Immunology Consultants Laboratory) and phycoerythrin (PE)-conjugated α-human IgG (Sigma). Cells were sorted on a BD Influx (BD Biosciences) FACS sorter. Appoximately $2x10^5$ GL+9 positive cells were collected and expanded for ~one week in the presence of puromycin and blasticidin before the next round of enrichment was carried out. There was no enrichment for GL+3 positive cells after several rounds of sorting so only the GL+9 positive cells were sequenced. Once the desired population had been obtained the chromosomal DNA was extracted from the cell culture using the GenElute Mammalian Genomic DNA Miniprep Kit (Sigma). The BG505-gp120 gene was PCR amplified from the genomic DNA and ligated back into the Gateway entry vector using CPEC cloning and transformed into top10 competent cells. Later in the design process Gibson assembly was substituted for CPEC cloning. Colonies were sequenced at Genewiz. The sequences were highly enriched for two clones, one containing the N137 glycan knockout by the mutation T139I and the other containing the N133 glycan knockout by the mutation T135A in addition to the T139I mutation. These constructs were called 2MUT (T332N, T139I) and 3MUT (T332N, T135A, T139I). Measuring the affinities of gp120-2MUT and gp120-3MUT against a panel of partially mutated PGT121 Abs (table S1) indicated that knocking out both glycans gave a larger boost in affinity compared to only the N137 glycan-KO so 3MUT was used for further designs.

In parallel to screening the error prone PCR library, a combinatorial library was created based on the structure of PGT122 in complex with BG505 SOSIP (PDB IDs 4NCO and 3J5M). Because the initial SOSIP structures were low resolution and structures of germline PGT121 showed light chain conformational changes we elected to do a saturation mutagenesis combinatorial library that would roughly cover the length of the V1 loop that could potentially interact with germline PGT121 Abs. The library was generated by PCR amplifying the BG505 SOSIP construct in two partially overlapping fragments. The C-terminal fragment was amplified with a primer containing the degenerate codon NNK at four positions in the V1 loop (V134, N136, I138, and D140) as well as a degenerate base encoding N or D at position 137. The two PCR products were ligated together using a second round of PCR, and this second PCR product was inserted into the pENTR vector as described above. The resulting construct was transferred to the pLenti CMVTRE3G puro Dest vector, and lentivirus was produced. Stable cells were stained with the

GL+3 Ab and α-cMyc, and double positive cells were sorted. This resulted in a binding population that was sequenced and found to be a single unique clone containing the mutations V134Y, N136P, I138L, D140N. This clone was called 5MUT (T332N, V134Y, N136P, I138L, D140N). These mutations were combined with the T139I mutation (6MUT) or the T135A/T139I mutations (7MUT).

Next, a saturation mutagenesis scanning library was created on the gp120-7MUT construct using site directed mutagenesis with the QuikChange kit (Agilent Technologies) with a unique NNK/MNN primer pair for each position that was scanned. 11 positions in the V1 loop (T132 to M142) and 10 positions in the V3 loop (T320 to Q328) were scanned and the resulting 21 reactions were pooled, purified, concentrated, and transformed into electroMAX DH5a-E competent cells and transferred to pLenti CMVTRE3G puro Dest as described above. This library was then stained separately with $GL_{CDR3mat}$, $GL_{CDR3rev4}$, or a Chimeric Ab containing the mature PGT121 heavy chain paired with the $GL_{CDR3rev4}$ light chain ($121_H/GL_{L\text{-}rev4}$), as well as α-cMyc for expression. Double positive cells were sorted and 3 mutations were enriched in the $GL_{CDR3mat}$ sort (N137F, T320F, Q328M) and two mutations were enriched in the $121_H/GL_{L\text{-}rev4}$ sort (N135R, Q328M) whereas a binding population was not obtained in the $GL_{CDR3rev4}$ sort. Combining these mutations with 7MUT resulted in $9MUT_A$ (7MUT + N137F/Q328M), $9MUT_B$ (7MUT + N135R/Q328M), and 10MUT (7MUT + N137F/T320F/Q328M). The $9MUT_B$ protein showed improved binding to $121_H/GL_{L\text{-}rev4}$ but worse binding to all other PGT121-class antibodies tested compared to 7MUT (from which $9MUT_B$ was derived) and so $9MUT_B$ was not selected for further use except as a control for the chimeric antibody (data not shown). Gp120-10MUT showed better binding to $GL_{CDR3mat}$ compared to gp120-$9MUT_A$ and T320F was used in subsequent designs with the exception of our SOSIP-$10MUT_A$ crystal structure, which lacks the T320F mutation.

Having established ~1 μM binding to the $GL_{CDR3mat}$ Ab with 10MUT our goal was to improve the immunogen to tolerate more variation within the H-CDR3. For this we created three more V1 loop combinatorial libraries each containing four NNK codons. The three libraries contained NNK codons at positions (A135/P136/F137/L138), (F137/L138/I139/N140), and (I139/N140/D141/M150). Each library was assembled from two partially overlapping ultramers (Integrated DNA Technologies) and ligated into the gp120-10MUT gene using gibson assembly (New England Biolabs). The three libraries were pooled and screened against $GL_{CDR3rev2}$ and $GL_{CDR3rev4}$ Abs. Sorting against the $GL_{CDR3rev4}$ Ab resulted in enrichment for the D141N mutation ($11MUT_A$) and sorting against the $GL_{CDR3rev2}$ resulted in enrichment for L139 and S140 with the most frequent clone containing the sequence N137/L138/L139/S140. When these mutations were combined with the D141N mutation as well as a T415V mutation, which we had identified as being beneficial for binding to PGT121 on an engineered outer domain construct (data not shown), it resulted in $11MUT_B$.

Development of BG505-SOSIP_MD39.

BG505 SOSIP "rare amino acid" library. The BG505 SOSIP "rare amino acid" library was synthesized at GenScript. It was first sorted against PG16 followed by a sort for a high PGT145/B6 binding ratio. The cells were expanded for 1 week and then sorted for either high PGT145/B6 or high PGT151/4025. After six rounds of sorting the library was sequenced (Genewiz). PGT145, PGT151, and PG16 Fabs contained HA epitope tags and were labeled with α-HA-PE (Miltenyi Biotec). B6 and 4025 Fabs contained V5 epitope tags and were labeled with α-V5-FITC (GeneTex).

BG505 SOSIP whole gene saturation mutagenesis. The whole gene saturation mutagenesis library was synthesized at Integrated DNA Technologies in four segments that each contained ~150 NNK codons that were cloned into the BG505-SOSIP gene using either CPEC or Gibson assembly which resulted in four libraries. NNK codons were barcoded with a silent mutation on each side. The libraries created from the second and third segments were combined into one. The first, second and third libraries had NNK codons covering residues Y39-N185, N186-R500 and K502-Q658, respectively. The library that covered gp41 (502-658) was sorted for high PGT145/cMyc, high PGT145/B6, and high PGT151/cMyc. The first gp120 library (39-185) was sorted for high PGT145/B6, and high PGT151/4025. The second gp120 library (186-500) was sorted for high PGT145/cMyc, high PGT145/B6, high PGT151/4025, and high PGT151/cMyc. The sorted libraries were sequenced and analyzed essentially as described previously (Jardine et al., 2016). Positions that enriched for the same amino acid against multiple different mAb sorts (E.g. PGT145(+)/B6(-) and PGT151(+)/4025(-)) were favored for testing in follow up combinatorial libraries or directly testing in recombinantly purified protein. Combinatorial libraries based on the next generation sequencing analysis were assembled from overlapping ultramers and sorted against the same antibodies described above.

Trimer-conjugated liposome synthesis and characterization.

Materials. Lipids 1,2-distearoyl-*sn*-glycero-3-phosphocholine (DSPC) and 1,2-dioleoyl-*sn*-glycero-3-[(N-(5-amino-1-carboxypentyl)iminodiacetic acid)succinyl] (nickel salt) (DGS-NTA(Ni)) were purchased from Avanti Polar Lipids (Alabaster, AL). Cholesterol was purchased from Sigma-Aldrich (St. Louis, MO).

Liposome synthesis. Lipids in chloroform at a 66.5:28.5:5 molar ratio of DSPC:cholesterol:DGS-NTA(Ni) were dried under nitrogen followed by incubation under vacuum for 18 hr at 25 °C. Lipid films were rehydrated with pH 7.4 PBS to a final concentration of 6.5 mM lipid and vortexed 30 s every 10 min for 1 hr at 50 °C. The resulting vesicles were passed through six freeze–thaw cycles between liquid nitrogen and a 50 °C water bath followed by extrusion 21 times through 0.1 µm pore polycarbonate membranes (Whatman Inc, Sanford, ME). Post-liposome formation, 6xHis tagged gp140 trimer was mixed with liposomes at a molar ratio of 42:1 exposed Ni-NTA:trimer (50% of total lipids were assumed to be exposed on the bilayer) and incubated for 2-4 hr at 4 °C. Unconjugated gp140 trimer was then purified away from conjugated liposomes via size exclusion chromatography using a Sepharose CL-2B resin (Sigma) or airfuge (Beckman-Coulter).

Liposome characterization. Total conjugated trimer was quantified by ELISA. Liposomes were treated with 1% triton-X and 100 mM imidazole in PBS containing 1% BSA to destabilize liposomes and Ni-6xHis tag interactions, respectively. Trimer from destabilized liposomes was captured on Nunc MaxiSorp plates with VRCO1 and detected by a mouse anti-6xHis IgG-HRP conjugate (R&D Systems. Minneapolis, MN). Trimer standards were run in parallel and used to calculate final trimer concentrations in each liposome preparation. For the calculation of the number of trimers per liposome, the total lipid concentration in the final liposome preparation was determined using a phospholipid quantification assay (Sigma). This was used to further calculate a theoretical number of monodisperse, unilamellar liposomes using the following equation for the number of lipids (N_{Total}) per liposome, where a = surface area of a single phospholipid head group (0.71nm), h = bilayer width (5nm), and d = liposome diameter:

$$N_{\mathrm{Total}} = \frac{4\pi\left[(d/2)^2\right] + 4\pi\left[(d/2) - h\right]^2}{a}$$

Trimer-conjugated liposomes were also characterized by cryoelectron microscopy (Jeol 2100F TEM) and dynamic light scattering (Wyatt Dyna Pro Plate Reader II) in the Swanson Biotechnology Center at the Koch Institute, MIT. To evaluate trimer antigenicity profiles post-liposome conjugation, intact liposomes were captured on Nunc MaxiSorp plates with VRCO1 (mouse Fc) in PBS containing 1% BSA and detected with various bNAbs or non-NAbs, followed by secondary detection with a goat anti-human IgG-HRP conjugate (Abcam, Cambridge, MA).

Ca^{2+}-flux measurements. Single cell suspensions of spleen and lymph nodes were prepared from mice expressing the predicted germline ($GL_{CDR3rev4}$) or mature heavy and light chain sequences of PGT121. B cells were enriched following the manufacturers instructions by negative selection using anti-CD43 microbeads (Miltenyi Biotec) and magnetized LS columns (Miltenyi Biotec). Enriched cells were washed once in PBS and then diluted in PBS to a concentration of 20 x 10^6 cells/ml. Fluo-4, AM, permeant (Thermo Fisher) and CellTrace Violet (Thermo Fisher) were added to cells to a final concentration of 0.5 µM. Cells were incubated in the dark at 37 °C. After 20 min, labeling was inactivated by the addition of complete medium (RPMI 1640 medium containing 10 mM HEPES and 6% serum) and incubated for 5 min at RT in the dark. Cells were centrifuged and resuspended in complete medium and incubated for an additional 20 min at 37 °C. Cells were centrifuged and resuspended in complete medium without phenol at a concentration of 20 x 10^6 cells/ml and 100 µl aliquots (2 x 10^6 cells) were prepared in FACS tubes. Ca^{2+} flux was detected by flow cytometry (BD LSRFortessa) as increases in fluorescence by Fluo-4 upon binding Ca^{2+} after the addition of stimuli, which was added in a volume of 100 µl to the cells. Ionomycin (final concentration of 1 µg/ml, Sigma) and biotinylated anti-mouse IgM (final concentration of 20 µg/ml, Jackson ImmunoResearch) with the subsequent addition of streptavidine (final concentration of 40 µg/ml, Jackson ImmunoResearch) was added as positive controls. Complete media and the irrelevant antigen

Ovalbumin (final concentration of 300 μg/ml, Sigma) was added as negative controls. Liposomes were added to cells to a final concentration of 300-, 30- and 3 μg/ml and trimers were added to cells to a final concentration of 200-, 20- and 2 μg/ml. Stimuli was added after 30 sec of acquiring un-stimulated cells on the flow cytometer. Ca^{2+}-flux data is presented as the ratio of MFI for Fluo4 and CTV.

Negative-stain electron microscopy. Purified SOSIP trimers, at concentrations between 1.0-1.5 mg/mL as determined by UV A_{280} and an extinction coefficient of $A_{280}^{0.1\%}$ 1.55, were thawed, diluted 1:100 in Tris-buffered saline, and stored on ice until negative-stain EM analysis using a protocol adapted from (de Taeye et al., 2015). Briefly, samples were applied to carbon-coated grids for 10 s, blotted with filter paper, and stained with 2% (w/v) uranyl for 45-60 s prior to blotting with clean filter paper. Data collection was performed using the FEI Tecnai T12 electron microscope and Tietz TemCam-F416 CMOS camera settings described in de Taeye et al. Between 5,000-15,000 single particles were analyzed using reference-free two-dimensional classification (Iterative MSA/MRA method; (Ogura et al., 2003)) and those particles resembling trimers were further classified as having a closed/compact appearance similar to BG505 SOSIP.664, open/breathing phenotype similar to B41 SOSIP.664, or non-native features characteristic of malformed or uncleaved trimers with weakly associated protomers (Pugach et al., 2015; Ringe et al., 2013). Reported native-like percentage is the sum of closed and breathing trimers as a fraction of all trimer particles.

Differential scanning calorimetry (DSC). DSC experiments were performed on a MicroCal VP-Capillary differential scanning calorimeter (Malvern Instruments). The HEPES buffered saline (HBS) buffer was used for baseline scans and the protein samples were diluted into HBS buffer to adjust to 0.25 mg/ml. The system was allowed to equilibrate at 20 °C for 15 min and then heat up till 90°C at a scan rate of 90°C/h. Buffer correction, normalization, and baseline subtraction were applied during data analysis using Origin 7.0 software. The non-two-state model was used for data fitting.

Protein complex formation. Fabs PGT124 and 35022 were produced by transient transfection of 293 FreeStyle™ cells (Invitrogen), and purified by affinity chromatography on a CaptureSelect LC-lambda column (ThermoFisher Scientific), followed by size exclusion chromatography with a S200, 16/60 column (GE Healthcare). SOSIP_MD39_10MUTA was mixed with a 20% molar excess of Fabs PGT124 and 35022, incubated on ice for 15 minutes and then deglycosylated with EndoH (NEB) at 37° in 200mM sodium chloride, 50mM sodium citrate, pH 5.5, for 35 minutes. The ternary complex was then purified by size exclusion chromatography as for the Fabs. The final sample was concentrated to 10.3 mg/mL.

Crystallization and data collection. The crystal used for data collection was obtained at 4° C in a sitting drop tray with precipitant of 5% Peg6000, 0.1M citric acid, pH 4.1. The crystal was briefly immersed in the well solution augmented with 30% Peg200 and flash-cooled in liquid nitrogen. Data were collected at the Advanced Photon Source, beamline 23-ID-D, and processed with HKL-2000 (Otwinowski and Minor, 1997) resulting in data to 4.5Å resolution with 98.4% completeness.

Structure solution and refinement. The structure was determined by molecular replacement with Phaser (McCoy et al., 2007) using model PDB 5CEZ (Garces et al., 2015) with model building carried out using Coot (Emsley et al., 2010). Initial rounds of refinement were carried out with Phenix (Adams et al., 2010), using the 5CEZ coordinates as reference model restraints, with group B factors and TLS refinement, while final rounds of refinement were carried out with Refmac5 (Murshudov et al., 2011), with reference model restraints, jelly body restraints, and TLS refinement. Statistics for data collection and final refinement are listed in Table S1.

ELISA to characterize antigenic profile of native-like trimers. 96-well plates were coated overnight at 4°C with 6x-His Epitope Tag Antibody (Thermofisher) at 2 mg/ml in PBS. Plates were washed 3 times with PBS, 0.05% Tween (PBS-T), and blocked with 10% milk PBS for 1h. Subsequently, 2 mg/ml of the purified His-tagged SOSIP protein was added for 2 h in 1% milk PBS-T, after which the plates were washed three times with PBS-T. Serial dilutions of mAbs in 1% milk PBS-T were added to the plates for 1 h, after which the plates were washed again three times with PBS-T before the addition of anti-human Fc region -conjugated alkaline phosphatase (Jackson Immunoresearch) at 1:1000 for 1 h. After four final

washes, binding was detected by the addition of alkaline phosphatase substrate and measured by absorbance at 405 nm.

Development of variable loop cocktail (VLC) trimers. Using BG505 SOSIP MD39 trimer as a base, a series of new trimers were engineered by replacing the immunodominant variable loops of the BG505 strain with loops from alternative strains. Given the vast number of HIV strains available, we created three separate criteria to guide our loop selection. For the first set of variable loop transplants, we cataloged the number of glycans within each variable loop and the length of each variable loop (Figure 7C). Certain combinations of variable loop lengths and glycans were observed more frequently than others across HIV strains (e.g. 20.48 % of HIV strains have a 14 amino acid variable loop 2 with one glycan, Figure 7C). We searched for strains that contain the most common loop length/glycan combination for each of the variable loops (V1,V2,V4,V5). For the second set of variable loop transplants, we searched for strains with variable loops of the same length and number of glycans as BG505, but with very different amino acid sequence and glycan positioning within the loops as compared to BG505. No single strain had all the same variable loop lengths and number of glycans as BG505, so we relaxed our criteria and matched each variable loop independently for this set of variable loop transplants only. For the third set of variable loop transplants, we searched for strains with exceptionally long variable loops (V1,V2,V4 must be ≥ 4 amino acids longer than BG505). Under each of these criteria, we were able to obtain one or two trimers that had a reasonable level of expression and formed well-behaved native-like trimers (Figure S7A). The loops of the VLCs are defined as: VLC-1 (V1: BES10.EF363127, V2: BL8157. DQ886035, V4: BF1P51.JQ250880, V5: CZM197.DQ388515), VLC-2(BG505. DQ208458), VLC-3 (PRLS08.FJ469757) VLC-4(GHJ193.AB231897), VLC-5(OUR2478P.EF165541). A region defined as 335-351 (HxB2) underneath V4 was included when transplanting V4, due to high variability and close contact with V4. Including BG505, we report a set of 5 trimers with diverse variable loops. One version of the VLC trimers that did not have the MD39 mutations, and instead contained an extra stabilizing disulfide (DS21: V120C-Q315C) in order to staple down the tip of the V3, this version of the VLC trimers was used in an accompanying manuscript (Escolano et al., 2016).

Structural alignment of trimers. The alignment of native-like trimers was done using the alignMolecules program from MSL (Kulp et al., 2012) and verified using PyMOL. The following residues from all three subunits were used to align 5CEZ, 4TVP and the MD39 structure reported here: 32-60+65-132+153-184+189-396+411-505+518-546+572-664.

Supplemental References

Adams, P.D., Afonine, P.V., Bunkoczi, G., Chen, V.B., Davis, I.W., Echols, N., Headd, J.J., Hung, L.W., Kapral, G.J., Grosse-Kunstleve, R.W., *et al.* (2010). PHENIX: a comprehensive Python-based system for macromolecular structure solution. Acta Crystallogr D *66*, 213-221.

Hoffenberg, S., Powell, R., Carpov, A., Wagner, D., Wilson, A., Kosakovsky Pond, S., Lindsay, R., Arendt, H., Destefano, J., Phogat, S., *et al.* (2013). Identification of an HIV-1 clade A envelope that exhibits broad antigenicity and neutralization sensitivity and elicits antibodies targeting three distinct epitopes. J Virol *87*, 5372-5383.

Kulp, D.W., Subramaniam, S., Donald, J.E., Hannigan, B.T., Mueller, B.K., Grigoryan, G., and Senes, A. (2012). Structural informatics, modeling, and design with an open-source Molecular Software Library (MSL). Journal of computational chemistry *33*, 1645-1661.

McCoy, A.J., Grosse-Kunstleve, R.W., Adams, P.D., Winn, M.D., Storoni, L.C., and Read, R.J. (2007). Phaser crystallographic software. J Appl Crystallogr *40*, 658-674.

Murshudov, G.N., Skubak, P., Lebedev, A.A., Pannu, N.S., Steiner, R.A., Nicholls, R.A., Winn, M.D., Long, F., and Vagin, A.A. (2011). REFMAC5 for the refinement of macromolecular crystal structures. Acta Crystallogr D *67*, 355-367.

Ogura, T., Iwasaki, K., and Sato, C. (2003). Topology representing network enables highly accurate classification of protein images taken by cryo electron-microscope without masking. Journal of structural biology *143*, 185-200.

Otwinowski, Z., and Minor, W. (1997). Processing of X-ray diffraction data collected in oscillation mode. Method Enzymol *276*, 307-326.

Quan, J., and Tian, J. (2014). Circular polymerase extension cloning. Methods in molecular biology *1116*, 103-117.

Ringe, R.P., Sanders, R.W., Yasmeen, A., Kim, H.J., Lee, J.H., Cupo, A., Korzun, J., Derking, R., van Montfort, T., Julien, J.P.*, et al.* (2013). Cleavage strongly influences whether soluble HIV-1 envelope glycoprotein trimers adopt a native-like conformation. Proceedings of the National Academy of Sciences of the United States of America *110*, 18256-18261.

Salmon, P., and Trono, D. (2007). Production and titration of lentiviral vectors. Current protocols in human genetics / editorial board, Jonathan L Haines [et al] *Chapter 12*, Unit 12 10.

Schiffner, T., de Val, N., Russell, R.A., de Taeye, S.W., de la Pena, A.T., Ozorowski, G., Kim, H.J., Nieusma, T., Brod, F., Cupo, A.*, et al.* (2016). Chemical Cross-Linking Stabilizes Native-Like HIV-1 Envelope Glycoprotein Trimer Antigens. Journal of virology *90*, 813-828.
