# Supplementary material for: HIV Vaccine Design to Target Germline Precursors of Glycan-Dependent Broadly Neutralizing Antibodies
Source: Immunity. 2016 Sep 20;45(3):483–96. doi: 10.1016/j.immuni.2016.08.016 (PMC5040827; doi:10.1016/j.immuni.2016.08.016)
Supplement: Document S2. Article plus Supplemental Information [file mmc2.pdf]

Immunity

Article

HIV Vaccine Design to Target Germline Precursors of Glycan-Dependent Broadly Neutralizing Antibodies

Highlights

- Developed mammalian cell display to design germline-targeting native-like trimers
- Germline-targeting trimers retain native-like antigenicity and structure
- Germline-targeting trimers prime glycan-dependent HIV bnAb responses in knockin mice
- Designed boosting schemes intended to induce bnAbs

Authors

Jon M. Steichen, Daniel W. Kulp, Talar Tokatlian, ..., Darrell J. Irvine, Michel C. Nussenzweig, William R. Schief

Correspondence

nussen@mail.rockefeller.edu (M.C.N.), schief@scripps.edu (W.R.S.)

In Brief

Elicitation of broadly neutralizing antibodies (bnAbs) is a critical HIV vaccine goal. Steichen et al. have developed immunogens that prime germline-precursor B cells for the bnAb PGT121 and can therefore initiate bnAb induction. The authors have also designed boosting immunogens to shepherd the antibody maturation to develop bnAbs.

Accession Numbers

5T3S

Steichen et al., 2016, Immunity *45*, 483–496
September 20, 2016 © 2016 The Authors. Published by Elsevier Inc.
http://dx.doi.org/10.1016/j.immuni.2016.08.016

CellPress

HIV Vaccine Design to Target Germline Precursors of Glycan-Dependent Broadly Neutralizing Antibodies

Jon M. Steichen,[1,2,3,11] Daniel W. Kulp,[1,2,3,11] Talar Tokatlian,[4,11] Amelia Escolano,[5,11] Pia Dosenovic,[5] Robyn L. Stanfield,[2,3,6] Laura E. McCoy,[1,2,3] Gabriel Ozorowski,[2,3,6] Xiaozhen Hu,[1,2,3] Oleksandr Kalyuzhniy,[1,2,3] Bryan Briney,[1,2,3] Torben Schiffner,[1,2,3] Fernando Garces,[2,3,6] Natalia T. Freund,[5] Alexander D. Gitlin,[5] Sergey Menis,[1,2,3] Erik Georgeson,[1,2,3] Michael Kubitz,[1,2,3] Yumiko Adachi,[1,2,3] Meaghan Jones,[1,2,3] Andrew A. Mutafyan,[4] Dong Soo Yun,[4] Christian T. Mayer,[5] Andrew B. Ward,[2,3,6] Dennis R. Burton,[1,2,3,7] Ian A. Wilson,[2,3,6,8] Darrell J. Irvine,[4,7,9,10] Michel C. Nussenzweig,[5,9,*] and William R. Schief[1,2,3,7,12,*]

[1]Department of Immunology and Microbial Science, The Scripps Research Institute, La Jolla, CA 92037, USA
[2]IAVI Neutralizing Antibody Center, The Scripps Research Institute, La Jolla, CA 92037, USA
[3]Center for HIV/AIDS Vaccine Immunology and Immunogen Discovery, The Scripps Research Institute, La Jolla, CA 92037, USA
[4]Koch Institute for Integrative Cancer Research, MIT, Cambridge, MA 02139, USA
[5]Laboratory of Molecular Immunology, The Rockefeller University, New York, NY 10065, USA
[6]Department of Integrative Structural and Computational Biology, The Scripps Research Institute, La Jolla, California, USA
[7]The Ragon Institute of Massachusetts General Hospital, Massachusetts Institute of Technology and Harvard University, Cambridge, MA 02139, USA
[8]Skaggs Institute for Chemical Biology, The Scripps Research Institute, La Jolla, CA 92037, USA
[9]Howard Hughes Medical Institute, Chevy Chase, MD 20815, USA
[10]Departments of Biological Engineering and Materials Science & Engineering, MIT, Cambridge, MA 02139, USA
[11]Co-first author
[12]Lead Contact
*Correspondence: nussen@mail.rockefeller.edu (M.C.N.), schief@scripps.edu (W.R.S.)
http://dx.doi.org/10.1016/j.immuni.2016.08.016

SUMMARY

Broadly neutralizing antibodies (bnAbs) against the N332 supersite of the HIV envelope (Env) trimer are the most common bnAbs induced during infection, making them promising leads for vaccine design. Wild-type Env glycoproteins lack detectable affinity for supersite-bnAb germline precursors and are therefore unsuitable immunogens to prime supersite-bnAb responses. We employed mammalian cell surface display to design stabilized Env trimers with affinity for germline-reverted precursors of PGT121-class supersite bnAbs. The trimers maintained native-like antigenicity and structure, activated PGT121 inferred-germline B cells ex vivo when multimerized on liposomes, and primed PGT121-like responses in PGT121 inferred-germline knockin mice. Design intermediates have levels of epitope modification between wild-type and germline-targeting trimers; their mutation gradient suggests sequential immunization to induce bnAbs, in which the germline-targeting prime is followed by progressively less-mutated design intermediates and, lastly, with native trimers. The vaccine design strategies described could be utilized to target other epitopes on HIV or other pathogens.

INTRODUCTION

A vaccine is needed for global HIV prevention. Broadly neutralizing antibodies (bnAbs) directed against relatively conserved epitopes in the otherwise highly antigenically variable HIV envelope (Env) glycoprotein trimer offer important guides for vaccine design. BnAbs have been isolated from a small minority of HIV-infected individuals and have been shown to protect against challenge in various animal models, but have not been induced by vaccination in humans or standard animal models (Burton and Hangartner, 2016; Mascola and Haynes, 2013; West et al., 2014). BnAbs recovered from natural infection are typically highly mutated (Klein et al., 2013a; Mouquet et al., 2010; Pancera et al., 2010; Scheid et al., 2009; Walker et al., 2011; Xiao et al., 2009; Zhou et al., 2010) and many also contain insertions and/or deletions (Kepler et al., 2014), owing to chronic stimulation of B cells by mutating Env. Many bnAbs also possess unusually long or short heavy-chain complementarity determining region 3 (CDR3) loops (Scheid et al., 2011; Walker et al., 2009, 2011; Wu et al., 2011; Zhou et al., 2010) and some are polyreactive (Haynes et al., 2005). Less mutated bnAbs with fewer unusual features have been engineered, offering more tractable goals for consistent vaccine elicitation (Georgiev et al., 2014; Jardine et al., 2016b; Sok et al., 2013). Overall, bnAb elicitation by vaccination presents a major challenge.

Recombinant native-like trimers are promising HIV vaccine components because they contain the conformational epitopes of most known bnAbs and lack many non-neutralizing epitopes present on less native constructs (Julien et al., 2013; Kong

This is an open access article under the CC BY license (http://creativecommons.org/licenses/by/4.0/).

et al., 2016; Kwon et al., 2015; Lyumkis et al., 2013; Pancera et al., 2014; Sanders et al., 2013; Scharf et al., 2015). However, native-like trimers have features that might impede bnAb induction; they are highly glycosylated and expose both strain-specific neutralizing epitopes and non-neutralizing epitopes. Immunization with native-like trimers in standard mouse, rabbit, and macaque models has thus far elicited either non-neutralizing antibodies (Hu et al., 2015) or neutralizing antibodies only against the immunogen strain (de Taeye et al., 2016; Sanders et al., 2015) analogous to the strain-specific responses to the seasonal flu vaccine in humans. Induction of HIV bnAbs will likely require development of vaccination strategies that focus responses to relatively conserved, sub-dominant epitopes and avoid or suppress responses to non-neutralizing and strain-specific epitopes.

Germline targeting, a vaccine priming strategy to initiate the affinity maturation of specific germline-precursor B cells, could help solve this immunofocusing problem by preferentially activating bnAb precursors (Dimitrov, 2010; Xiao et al., 2009). The strategy aims to activate bnAb-precursor B cells, select productive (bnAb-like) somatic mutations, and produce memory B cells that can be boosted subsequently to select additional productive mutations (Dosenovic et al., 2015; Jardine et al., 2015). For some bnAbs, inferred precursors have affinity for Env from particular HIV isolates (Andrabi et al., 2015; Doria-Rose et al., 2014; Gorman et al., 2016; Liao et al., 2013), facilitating design of priming immunogens based on Env from those isolates (Haynes et al., 2012). For other bnAbs, efforts to identify wild-type (WT) Env that bind inferred precursors have failed (Hoot et al., 2013; Jardine et al., 2013; McGuire et al., 2013; Scheid et al., 2011; Xiao et al., 2009; Zhou et al., 2010). These latter cases require design of modified Env to serve as a priming immunogen (Dimitrov, 2010; Pancera et al., 2010; Xiao et al., 2009; Zhou et al., 2010). Proof of principle that designed germline-targeting immunogens can activate their intended precursors and generate a potentially boostable memory response was recently demonstrated in knockin mice with B cell precursors for VRC01-class bnAbs directed to the CD4-binding site (Dosenovic et al., 2015; Jardine et al., 2015; McGuire et al., 2016). After a germline-targeting prime, induction of bnAbs is expected to require a succession of boosts, driving a succession of germinal-center reactions, in order to select sufficient mutations (Dimitrov, 2010; Dosenovic et al., 2015; Haynes et al., 2012; Jardine et al., 2013; 2015; 2016b; Klein et al., 2013b; Liao et al., 2013; McGuire et al., 2013; 2016; Pancera et al., 2010; Wu et al., 2011; Xiao et al., 2009; Zhou et al., 2010). Supporting the concept that sequential immunization with different immunogens will be required to develop a bnAb response, native-like trimers but not germline-targeting immunogens were found to boost near-bnAb B cells (bearing a mature VRC01-class bnAb heavy chain) to induce cross-neutralizing Abs (Dosenovic et al., 2015).

Glycan-dependent bnAbs in general, and N332-supersite bnAbs in particular, are important targets for germline-targeting vaccine design. In a recent longitudinal study of HIV infection in Africa, more than half of the HIV-infected individuals who produced bnAb responses produced them against glycan-directed epitopes, the majority of which were within the N332 supersite (Landais et al., 2016). The prevalence of N332-supersite bnAb responses is probably due in part to the high accessibility of their epitopes on top of the trimer.

Among N332-supersite bnAbs, PGT121-class bnAbs have been particularly well characterized, providing strong rationale for germline-targeting efforts. PGT121-class bnAbs are among the most potent bnAbs (Mouquet et al., 2012; Walker et al., 2011), and PGT121 delivered passively to macaques protects against SHIV (simian-human immunodeficiency virus) infection (Moldt et al., 2012; Shingai et al., 2014) and can suppress viremia when delivered after infection (Barouch et al., 2013; Shingai et al., 2013). However, PGT121-class inferred precursors show no measureable affinity for WT Env proteins that have been evaluated (Mouquet et al., 2012; Sok et al., 2013). Thus, development of a priming immunogen for PGT121-class precursors requires either design of a modified Env or identification of a natural Env with PGT121-class germline-binding capacity. Crystal structures have been determined for several PGT121-class bnAbs in complex with either BG505 SOSIP native-like trimers or gp120 (Garces et al., 2015; Garces et al., 2014; Julien et al., 2013; Kong et al., 2016; Pancera et al., 2014) and for unliganded structures of two germline-reverted PGT121 variants (Mouquet et al., 2012; Sok et al., 2013), providing critical information to guide design of modified Env for PGT121-class germline targeting.

PGT121-class bnAbs interact with conformationally flexible structures on HIV Env, including several glycans and the V1 variable loop, making computational design of germline-targeting Env challenging. Here, we developed a structure-guided directed evolution approach, by using mammalian cell surface display, to design PGT121-class germline-targeting stabilized-trimer immunogens. We multimerized these trimers on liposomes and evaluated trimer and liposome immunogens via biophysical, structural, and ex vivo B cell activation analyses. We further evaluated germline-targeting trimers by vaccination in PGT121 inferred-germline knockin mice. Our design process produced design intermediates with increasing levels of epitope modification between WT and germline-targeting trimers. These results led to our hypothesizing prime-boosting strategies in which a germline-targeting prime is followed by boosts with progressively less modified design intermediates and then with WT Env, followed ultimately by a cocktail of Env variants to expand breadth. Evaluation of several of these prime-boosting strategies in PGT121 germline and chimeric knockin mice is described in a related study (Escolano et al., 2016).

RESULTS

Design of Germline-Targeting gp120s

We identified mammalian cell surface display as a desirable platform for engineering modified HIV Env constructs with affinity for inferred-germline PGT121 Abs because it should allow for optimization of monomeric or multimeric antigens bearing mammalian glycans (Chen et al., 2008). Therefore, we developed a lentivirus-based mammalian-cell-surface-display method to carry out directed evolution of HIV gp120 monomers and gp140 trimers (Figure S1). Structural analysis of the PGT121 interaction with gp120 (Julien et al., 2013; Pancera et al., 2014) led us to hypothesize that the V1 and V3 loops were the key sites for germline-targeting mutations. For selection agents, we assembled a collection of six germline-reverted Abs, all using heavy-chain genes VH4-59, D3-3, and J6 and light-chain genes V3-21 and J3, with varying degrees of mutation in the D gene and L-CDR3

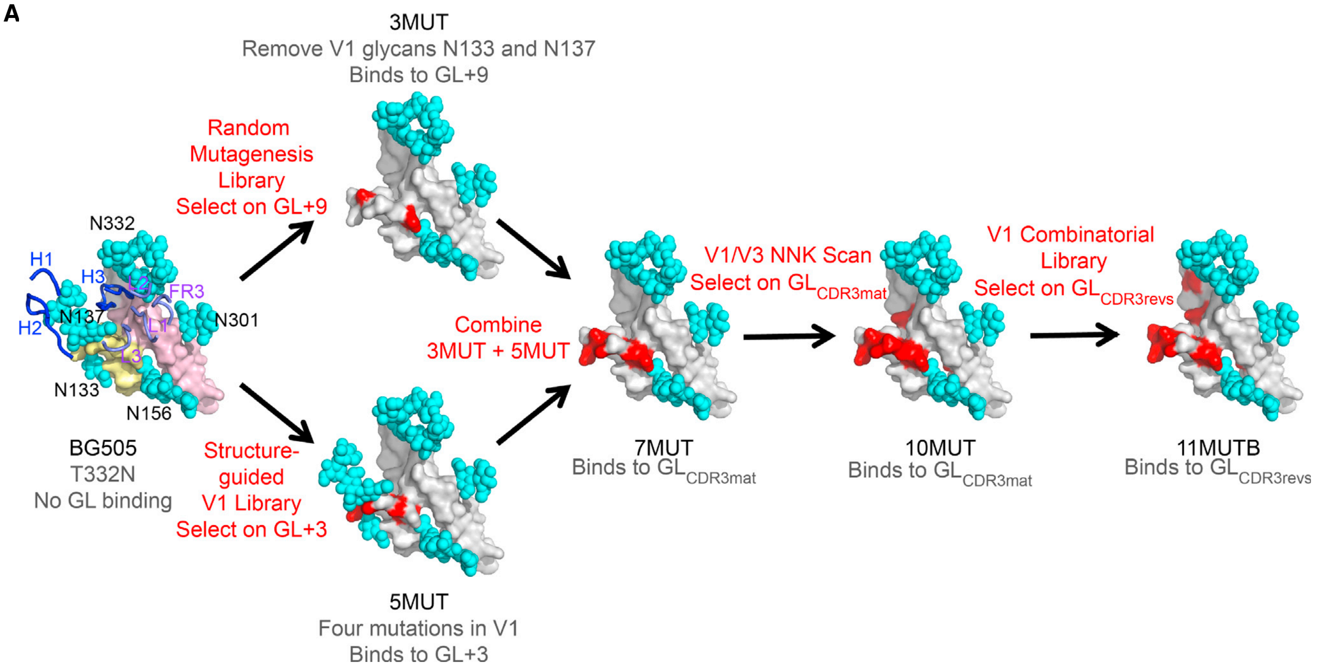

B

Mature → Germline

BG505-gp120	PGT121	3H3L	GL+9	GL+3	$GL_{CDR3\text{-}mat}$	$GL_{CDR3\text{-}rev5}$	$GL_{CDR3\text{-}rev4}$	$GL_{CDR3\text{-}rev3}$	$GL_{CDR3\text{-}rev2}$	$GL_{CDR3\text{-}rev1}$
WT (T332N)	7.5	250	28,000	>128,000	>128,000	>8,000	>84,000	>128,000	-	>8,000
3MUT	4.6	19	1,600	>28,000	>21,000	-	>28,000	>11,000	-	-
5MUT	5.7	2.5	18	WB	WB	-	>34,000	-	-	-
7MUT	1.2	0.25	3	12,200	44,000*	-	>36,000	-	-	-
10MUT	0.59	0.04	1.2	1,200	790	WB	>150,000	WB	>150,000	WB
11MUTB	0.15	0.075	0.6	600	840	7,700*	WB	3,000	-	5,200*

Figure 1. Mammalian-Display Directed-Evolution Design Pathway for PGT121 Germline-Targeting Env-Based Immunogens

(A) Models of the PGT121 epitope are shown for each immunogen, with positions of germline-targeting mutations colored red and glycans depicted with cyan spheres. The epitope of BG505 is colored yellow (variable loop 1) and pink (variable loop 3). The paratope of PGT122 is mapped onto the epitope of BG505 and shown in tube representation (heavy chain, blue; light chain, purple).

(B) Binding K_Ds of mature, intermediately mutated, and germline-reverted variants of PGT121 for BG505 gp120 and germline-targeting gp120s, determined by SPR. SPR K_Ds are the average of two or three experiments. Asterisk, complex binding kinetics; WB, weak binding; -, not done.

and with differences in the non-templated regions at the V-D and D-J boundaries (Figure S2). Ranked by similarity to the germline D and L-CDR3 sequences, these Abs are $GL_{CDR3rev1}$ (most similar to germline D and L-CDR3), $GL_{CDR3rev2}$, $GL_{CDR3rev3}$, $GL_{CDR3rev4}$, $GL_{CDR3rev5}$, and $GL_{CDR3mat}$ (germline V and J genes but with mature CDR3 loops). We began by screening libraries based on the template genes BG505 T332N gp120 and BG505 SOSIP T332N gp140 (Sanders et al., 2013), in which the conserved glycosylation site at position 332 absent in BG505 was introduced (Figure 1A). These molecules had no detectable affinity for germline-reverted PGT121 Abs (Figure 1B and Table S1). Therefore, we employed a "bootstrapping" approach: for initial screening we utilized two variants of $GL_{CDR3mat}$, one with nine PGT121 light-chain mutations (GL+9, with 28 μM affinity for BG505 T332N gp120) and another with three light-chain mutations (GL+3, no detectable affinity for BG505 T332N gp120) (Figure S2). Screening a gp120 random mutagenesis library for binding to GL+9 led to the molecule 3MUT, with mutations T135A and T139I, which eliminate the V1 loop glycosylation sites at positions 133 and 137 (Figure 1A). Screening a gp140 structure-guided V1 loop library for binding to GL+3 led to the isolation of 5MUT, with four different mutations (V134Y, N136P, I138L, and D140N) in the V1 loop. Combining the mutations in 3MUT and 5MUT produced 7MUT gp120, our first construct with quantifiable affinity for $GL_{CDR3mat}$ (K_D = 44 μM, Figures 1A and 1B). To improve this affinity further, we screened a gp120 V1- and V3-loop saturation mutagenesis library for binding to $GL_{CDR3mat}$; combining the most enriched mutations (N137F, T320F, and Q328M) with 7MUT

produced 10MUT, with $K_D \approx 1$ μM for $GL_{CDR3mat}$ (Figures 1A and 1B). Finally, to increase affinity and breadth, we screened a gp120 V1 loop directed mutagenesis library for binding to $GL_{CDR3rev2}$ and $GL_{CDR3rev4}$ (Figure S2). This approach culminated in 11MUT$_B$, with K_Ds of ~5 μM, ~3 μM, and ~8 μM for $GL_{CDR3rev1}$, $GL_{CDR3rev3}$, and $GL_{CDR3rev5}$, respectively, and detectable but not quantifiable binding to $GL_{CDR3rev4}$ (Figure 1A, Figure S3, and Table S1). Thus, mammalian display-directed evolution enabled the design of germline-targeting gp120 molecules with appreciable affinity for PGT121 germline-reverted antibodies.

Design of Stabilized and Germline-Targeting Trimers

For initial design of germline-targeting and boosting trimers, we transferred the germline-targeting mutations from the gp120 versions of 3MUT, 5MUT, 7MUT and 10MUT onto the BG505 SOSIP trimer platform. These molecules displayed characteristics of native-like trimers, such as high affinity for the trimer-specific bnAb PGT151 (Falkowska et al., 2014) and a melting temperature (T_m) similar to that of BG505 SOSIP (Figure 2A). Furthermore, all had similar monovalent affinities for PGT121 and $GL_{CDR3mat}$ as their gp120 counterparts (Figure 2A), indicating that the germline-targeting mutations were transferable to a native-like trimer.

In addition to binding bnAb putative precursors, germline-targeting trimers should have an otherwise native-like antigenic profile, with high affinity for bnAbs and no significant affinity for non-neutralizing antibodies directed to epitopes exposed on monomeric gp120 but buried or conformationally absent on the trimer. BG505 SOSIP gp140, the trimer on which our PGT121-class germline-targeting designs were based, displays undesirable binding to V3 non-neutralizing antibodies (Sanders et al., 2013) (Figure 2B and Figure S4) and induces non-neutralizing V3 responses in mice, rabbits, and macaques (de Taeye et al., 2016; Hu et al., 2015; Sanders et al., 2015), indicating that this trimer samples conformational states that expose non-neutralizing epitopes. Furthermore, BG505 SOSIP gp140 displayed on mammalian cells via a PDGFR linker showed strong binding to trimer-structure-dependent bnAbs (PGT151 and PGT145) (Falkowska et al., 2014; Walker et al., 2011) but also to non-neutralizing antibodies directed to the V3 loop (4025) (Gorny et al., 2011) and the CD4-binding site (b6) (Barbas et al., 1992) (not shown), suggesting the coexistence on the cell surface of native-like trimers along with non-native trimers, dimers, and/or monomers. We also found that adding germline-targeting mutations to BG505 SOSIP reduced the already modest expression by 50% (Figure 2A). Therefore, we sought to use mammalian-display directed evolution to improve the antigenic profile, thermal stability, and expression of the BG505 SOSIP trimer and germline-targeting trimers.

Our trimer improvement effort focused on two types of libraries: (1) whole-gene saturation mutagenesis libraries and (2) a combinatorial library sampling the one or two most common HIV residues at Env positions where BG505 uses rare (frequency < 10%) HIV residues (Figure 2C). The rare library, which allowed variation at eleven positions in gp120 and two in gp41, was screened for binding to trimer-structure-preferring bnAbs PGT145, PGT151, and PG16 and for lack of binding to non-neutralizing antibodies b6 and 4025. This produced the Rare3 clone with five mutations in gp120 (T106E, M271I, F288L, T290A, N363Q) and with the expression yield improved by a factor of ~2 and the T_m increased by 1.4°C (Figure S5). The saturation mutagenesis library was constructed in three segments, two covering gp120 and one for gp41 (Figure 2C). Next-generation sequencing and bioinformatics were employed to analyze the results of the first two sorts (Jardine et al., 2016a), and Sanger sequencing was used to identify enriched clones that survived four or five sorts. Enriched mutations from both sequencing methods were combined and tested in soluble trimers and were also assembled into combinatorial libraries and re-screened with the same antibodies as before. The gp41 library produced MD2, with an L568D point mutation that increased expression levels by a factor of ~4, and MD33, with four additional mutations (F519S, A561P, V570H, and R585H) that increased the T_m by 4°C and improved expression by a factor of ~7, relative to BG505 SOSIP (Figure S5). The gp120 library produced MD16, with three mutations (F223W, R304V, and A319Y) and reduced binding to V3 non-neutralizing antibodies (Figure S4). Finally, mutations from Rare3, MD16, and MD33 were combined to produce MD39 with 11 mutations (F223W and T290A did not improve the biophysical properties of the trimer and were excluded; data not shown). Compared to BG505 SOSIP.D664, the MD39 yield improved by a factor of ~7, T_m increased by 10°C, and antigenic profile improved, with reduced V3 Ab reactivity and similar bnAb binding except for slightly reduced affinities for V2 apex bnAbs (Figures 2A and 2B and Figure S5)

Combining the MD39 mutations with germline-targeting mutations produced germline-targeting trimers with improved properties. MD39-10MUT had a 6-fold-improved yield and 6°C higher T_m as compared to 10MUT (Figure 2A and Figure S5). Our most advanced germline-targeting trimer, MD39-11MUT$_B$, the only trimer with detectable affinity for five of six PGT121 germline reverted variants tested (Figure 2B), had excellent yield, thermal stability, and antigenic profile (Figures 2A and 2B). Directed evolution therefore produced native-like trimers with improved potential functionality via both stabilization and germline-targeting mutations.

Structural Analysis

To ascertain whether stabilized, PGT121 germline-targeting trimers maintain native-like structure, we conducted crystallography and electron microscopy (EM) studies. Negative-stain EM two-dimensional (2D) classification revealed that all four trimers tested (MD39, 10MUT, MD39-10MUT, and MD39-11MUT$_B$) were characterized by a high fraction (≥95%) of native-like structural features and were similar in appearance to BG505 SOSIP (Figure 3A). The MD39 mutations improved the structural uniformity of the 10MUT trimer; the amount of flexible, native open conformations dropped from 35% to 5% between 10MUT and MD39-10MUT (see the Experimental Procedures for a description of the 2D classification system). Our best germline-targeting trimer, MD39-11MUT$_B$, exhibited 100% native closed conformations and was indistinguishable from BG505 SOSIP by EM. For higher resolution analysis, we solved a 4.5 Å resolution crystal structure of MD39-10MUT$_A$, a variant of MD39-10MUT with one mutation added and another removed (see Supplemental Experimental Procedures), complexed with 35O22 and PGT124 (Garces et al., 2014; Sok et al., 2013). Although this resolution precluded analyses of

A

	Dissociation constant K_D (nM)									
	VRC01	121	GL CDR3mat	128	151	145	1400	Tm (°C)	Yield PEI	Yield fectin
BG505	131	22	-	8	10	6	2	67	1	1
3MUT	101	5	>13,000	3	9	3	1	67	-	1.8
5MUT	195	3	>13,000	5	16	3	1	65	-	0.62
7MUT	151	1.5	>13,000	4	14	3	1	66	-	0.72
10MUT	115	0.7	630	3	20	3	1	67	-	0.54
MD39	124	21	>13,000	12	11	20	12	77	7.5	6.5
MD39-10MUT	142	0.8	1,210	3	35	15	6	73	-	3.4
MD39-11MUTB	146	<0.016	563	1	22	20	7	71	7.8	5.3

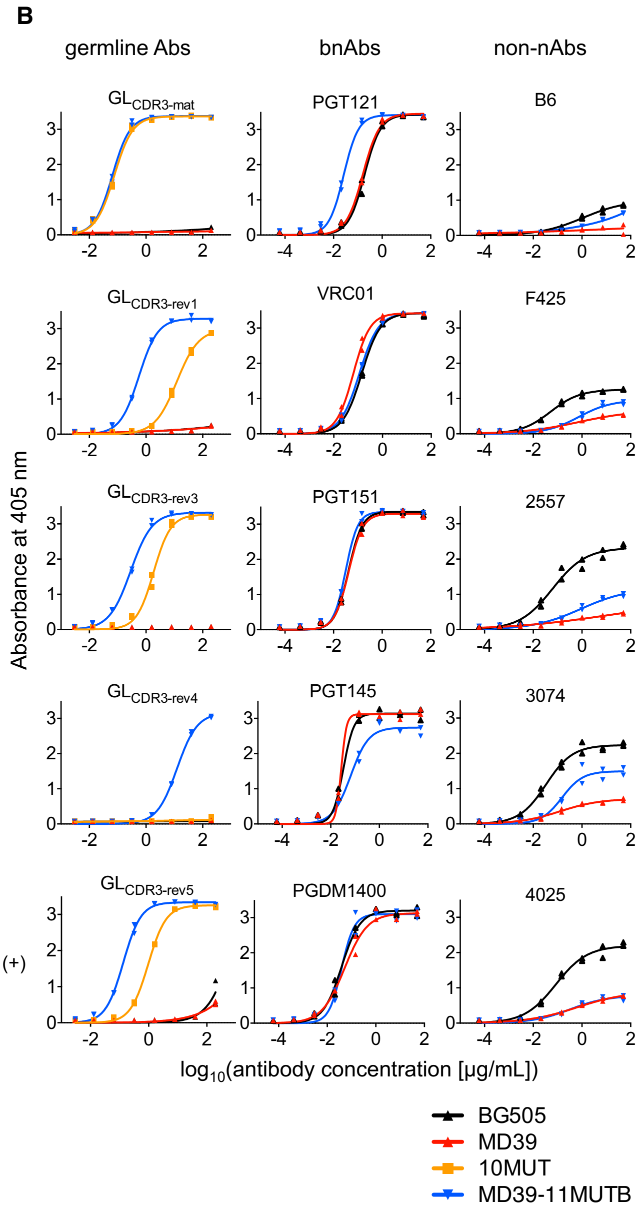

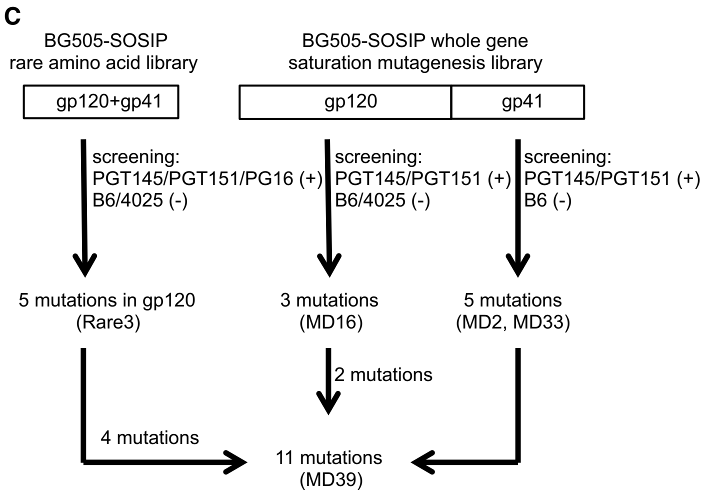

Figure 2. Design of Mutations to Stabilize BG505-SOSIP and Germline-Targeting Native-Like Trimers

(A) Biophysical properties of stabilized BG505-SOSIP and germline-targeting trimers. Antigenic profile was assessed by SPR, thermostability measurements were made by DSC, and expression was determined as yield of purified protein relative to BG505-SOSIP made with PEI or 293Fectin transfection reagents in 293F cells. Monovalent K_Ds were measured by SPR with trimer ligand and Fab analyte except for PGT145 and PGDM1400, for which monovalent K_Ds were determined with IgG ligand and trimer analyte. For PGT151, a one-to-one binding model gave a relatively poor kinetic fit.

(B) Antigenic profile of stabilized BG505-SOSIP and germline-targeting trimers by ELISA. Data are representative of two independent experiments, each done in duplicate.

(C) Mammalian display-directed evolution design pathways for engineering stabilized native-like trimers.

side-chain conformations, and the interface between trimer and PGT124 could not be analyzed due to missing V1 loop density, the structure accurately determined the backbone positions for most (1,659 of 1,692) residues of gp140. Superposition of the gp140 backbones in this structure and in the 3.0 Å structure of BG505 SOSIP N137A complexed with 3H109L and 35O22 (PDB: 5CEZ) or the 3.1 Å structure of BG505 SOSIP bound to PGT122 and 35O22 (PDB: 4TVP) gives backbone root-mean-square deviation values of 0.7 and 1.1 Å, respectively (Figure 3B). We conclude that MD39-10MUT$_A$, with 20 mutations relative to BG505 SOSIP T332N (Figure 3C), retains an overall native-like conformation.

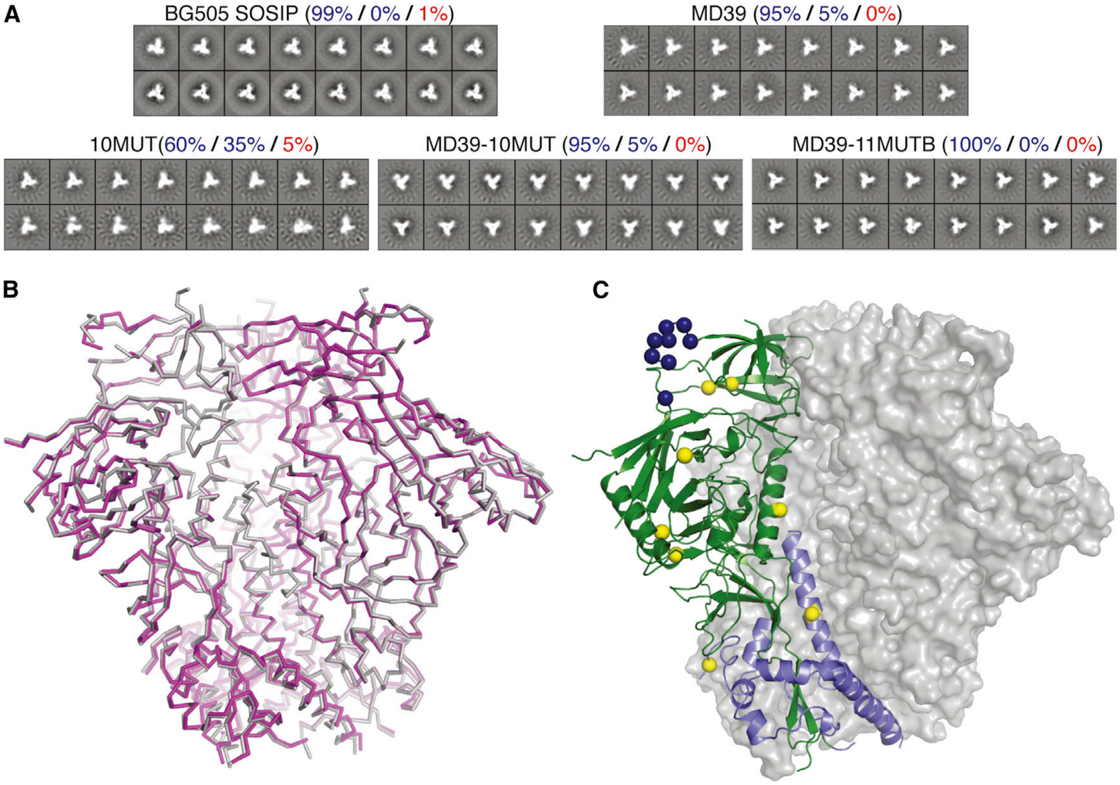

Figure 3. Structural Analysis of Stabilized Germline-Targeting Trimers

(A) Negative-stain EM analysis of the indicated trimers, in which the 2D class averages are shown and classified as percent closed native-like (blue), partially open native-like (blue), or non-native (red) (Pugach et al., 2015). We observed ± 5% variation between experiments.

(B) The crystal structure of a stabilized germline-targeting trimer (MD39-10MUT$_A$, in purple) is shown aligned to BG505-SOSIP (PDB: 5CEZ), in gray.

(C) Crystal structure of MD39-10MUT$_A$ (one subunit of gp41 is shown as a purple cartoon and one subunit of gp120 is shown as a green cartoon) highlighting the MD39 stabilizing mutations in yellow spheres and germline-targeting mutations in blue spheres.

Liposome Platform

We have previously found that highly multimeric particulate immunogens are superior to trimeric immunogens for B cell activation ex vivo (Jardine et al., 2013) and for generation of antigen-specific memory B cells after immunization in vivo (Jardine et al., 2015). Therefore, we developed trimer-conjugated liposomes to improve the immunogenic potential of our germline-targeting trimers. Trimers with a C-terminal His-tag were attached to 145-nm mean diameter uni-lamellar liposomes (DSPC [1,2-distearoyl-sn-glycero-3-phosphocholine], 66.5%; cholesterol, 28.5%; DGS-NTA(Ni), 5%) via the histidine-Ni-NTA interaction. On average, 522 ± 92 trimers were attached to each liposome. Cryo-EM examination of trimer-decorated liposomes confirmed the dense particulate array of trimers (Figure 4A). ELISA analysis on intact vesicles indicated that trimer-decorated liposomes maintained the native-like antigenic profile and germline-binding properties of the soluble trimers (Figure 4B).

Ex Vivo B Cell Activation

To determine whether germline-targeting trimers or trimer liposomes can specifically activate germline or mature PGT121 B cells, we conducted ex vivo experiments with B cells harvested from PGT121 GL$_{CDR3rev4}$ knockin mice, PGT121 bnAb knockin mice, and wild-type (WT) mice (Escolano et al., 2016) (PGT121 GL$_{CDR3rev4}$ is referred to as GL$_{HL}$121 in Escolano et al.). B cell activation was measured by a Ca^{2+}-flux assay (Ota et al., 2012) for MD39, 10MUT, and MD39-11MUT$_B$ (as trimers and trimer liposomes) and was compared to positive control activators (ionomycin and IgM) and negative control activators (ovalbumin) (Figure 5). Soluble trimers did not specifically activate germline PGT121 B cells (Figure S6) but did activate PGT121 bnAb B cells in a dose-dependent manner (Figure S6), with the strongest activation by MD39-11MUT$_B$, in accordance with its higher affinity for PGT121 as compared to MD39 and 10MUT (Figure 2C). MD39-11MUT$_B$ liposomes activated PGT121 germline B cells at concentrations as low as 0.3 μg/mL, whereas MD39 or 10MUT liposomes failed to activate at concentrations up to 300 μg/mL (Figure 5 and Figure S6). We conclude that MD39-11MUT$_B$ liposomes have promise as a PGT121-class germline-targeting prime.

Priming Germline PGT121 Responses in Knockin Mice

To determine whether germline-targeting trimers can activate germline PGT121 B cells in vivo, we conducted priming immunizations in PGT121-GL$_{CDR3rev4}$ knockin mice (Escolano et al., 2016). A combined five mice were immunized with 10MUT SOSIP, in two separate experiments, and four mice were immunized with MD39-11MUT$_B$ SOSIP. In a control experiment, six

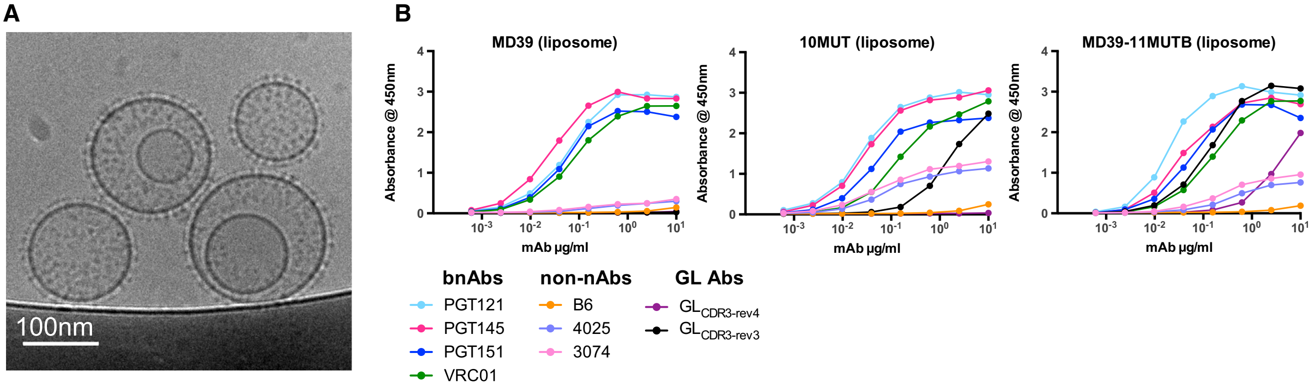

Figure 4. Structure and Antigenicity of Trimer Liposomes

(A) Cryo-EM image of MD39-11MUT_B trimer liposomes.

(B) ELISA analysis of trimer liposomes for MD39, 10MUT, and MD39-11MUT_B. Data are representative of two experiments.

mice were immunized with BG505 SOSIP. Two weeks after a single immunization, the sera was analyzed for immunogen- and epitope-specific responses. Sera of BG505-SOSIP-immunized mice showed no detectable binding to the BG505 SOSIP immunogen (Figure 6). In contrast, 4/5 10MUT-immunized mice and 4/4 11MUT_B-immunized mice showed epitope-specific serum responses to either 10MUT-gp120 or MD39-11MUT_B SOSIP (Figure 6). We conclude that germline-targeting mutations, such as those in 10MUT and 11MUT_B, are required for activation of inferred PGT121 germline B cells in vivo by BG505-based native-like trimers.

Sequential Boosting Strategies

As noted above, induction of bnAbs after a germline-targeting prime is expected to require sequential boosting with epitope variants to mature the response. With PGT121-class germline-targeting candidates (10MUT and 11MUT_B) in hand, we developed boosting strategies aiming to select PGT121-like mutations and induce bnAbs. We hypothesized that any sequential immunization strategy starting with a germline-targeting trimer should end with a native-like trimer, such as BG505 MD39 SOSIP, so as to select mutations productive for high-affinity interaction with native trimers present on circulating HIV strains. However, in order for PGT121-class antibodies to engage their epitope including the N137 glycan on the V1 loop, such antibodies must accommodate V1 loops diverse not only in sequence but also in length and number of glycosylation sites (Figures 7A–7C), implying that boosting with a single native-like trimer bearing a single V1 loop might not be sufficient. Indeed, boosting only with a BG505 native-like trimer would present a V1 loop that is significantly shorter than most (Figure 7B). Furthermore, modeling of variable loops and glycan conformations (not shown) suggested that diversity in the V2 and V4 loops might potentially impact the PGT121 epitope by altering conformational sampling of the V1 loop or N332 glycan, respectively (Figures 7A–7C), and immunodominant responses involving V2 or V4 could potentially sterically interfere with PGT121-class boosting. On the basis of these considerations, we hypothesized that a cocktail of native-like trimers displaying diverse variants of the V1, V2, and V4 loops, especially variants within hotspots of more frequently occurring combinations of length and number of glycosylation sites (Figure 7B), might be needed to select PGT121-class mutations favoring neutralization breadth. We therefore designed and produced four native-like trimers based on BG505 MD39 SOSIP and containing diverse loops for V1, V2, V4, and V5 (Figure 7C, Figures S7A and S7B, and Supplemental Experimental Procedures). These trimers, together with BG505 MD39 SOSIP, form a five-member variable loop cocktail (VLC) that might broaden PGT121-like responses initiated by a germline-targeting trimer (Figure 7D).

We then considered the question of what intermediate boosts, if any, might be employed between a germline-targeting prime and a native-like trimer. Our germline-targeting design intermediates become increasingly more native-like in the PGT121 epitope (e.g., 7MUT, 5MUT, and 3MUT have six, four, and two epitope mutations, respectively), but the 5MUT and 3MUT mutations are mutually exclusive (3MUT lacks two V1 glycans while 5MUT has those glycans but has four other V1 loop mutations) (Figure 1A). These considerations impose directionality on any boosting scheme (e.g., 7MUT should not be used after 5MUT or 3MUT or WT and 3MUT should not be used after 5MUT), thus limiting the number of possible schemes (Table S3). Considering only the most efficient directional schemes, those employing boosting pairs that differ by more than one mutation or involve substantial affinity changes (Table S4), we identified a total of seven potential boosting schemes (Figures 7E).

We sought to rank these schemes to allow prioritization for experimental testing. We reasoned that the least mutated antibody that shows measurable affinity for all of the potential boosting immunogens, GL+9, could serve as a proxy for intermediate PGT121-class antibodies developing after a germline-targeting prime and before a native-like boost. We further reasoned that the affinity drop, the ratio of GL+9 affinities for two immunogens, could be used to estimate the likelihood of successfully boosting memory B cells when the two immunogens are used in sequence (e.g., the GL+9 K_Ds for 7MUT and 3MUT are 3 nM and 1,600 nM respectively, so when immunizing with 7MUT followed by 3MUT, the affinity drop would be 1,600/3 = 530). One expects that a boost immunogen with very different epitope structure from the previous immunogen might result in too large an affinity

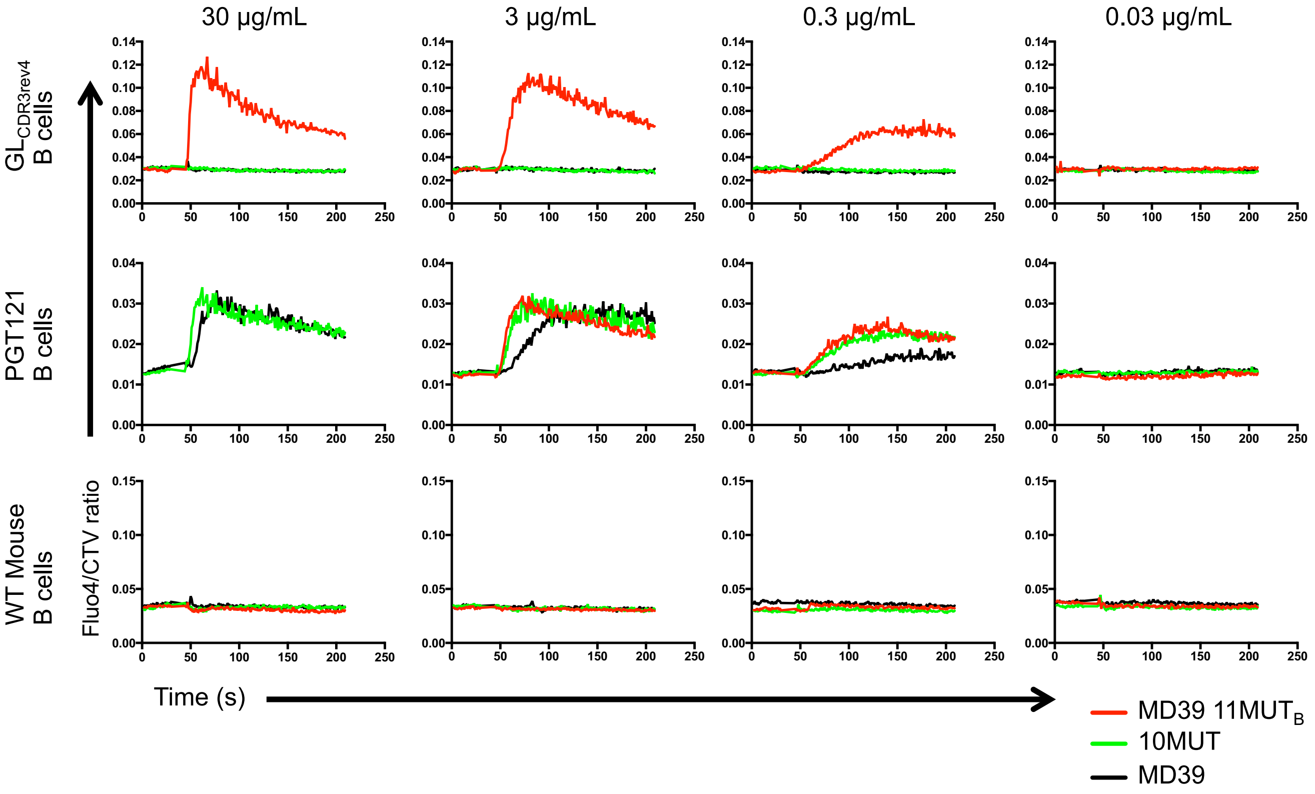

Figure 5. Ex Vivo B Cell Activation Assay

Ca^{2+} flux transients detected as increases in Fluo-4 fluorescence after addition of trimer liposomes (MD39, 10MUT, MD39-11MUT$_B$) at the indicated gp140 concentrations. Data are shown for germline-reverted PGT121 (GL$_{CDR3rev4}$) B cells (top), mature PGT121 B cells (middle), and WT mouse B cells (bottom). Data are representative of two experiments.

drop to activate memory B cells generated by the prior immunogen. We estimated the affinity drops for all seven boosting schemes (Figure 7E), ranked them according to the largest affinity drop in that boosting scheme, and listed the three most likely to succeed (Figure 7F).

In collaborating work, Escolano et al. (2016) evaluated boosting schemes following the 10MUT trimer prime in PGT121 germline (GL$_{CDR3-rev4}$) knockin mice and PGT121 mature-heavy-and-germline-light-chain knockin mice. Relying on the directionality of the boosting immunogens developed here, Escolano et al. used serum ELISA against boost candidates after each immunization to select the most native-like directional boost for which at least weak serum reactivity could be detected; that process resulted in the testing of the second scheme in Figure 7F and the finding that this scheme induces PGT121-like bnAbs with substantial breadth and potency. Although the first scheme in Figure 7F remains to be tested, the data in Escolano et al. support the validity of the logic underlying these boosting schemes.

We note that the affinity drop analysis also provides clues as to how to improve boosting schemes: to minimize the probability of a boost failure at a high affinity drop, one could redesign immunogens to equalize the affinity drops in any given scheme. Thus the germline-targeting design process is capable of defining potential boost immunogens and directional boosting schemes, and it can guide prioritization and improvement of such schemes.

DISCUSSION

Germline-targeting vaccine design offers the potential to initiate the induction of specific classes of protective antibodies against HIV or other pathogens that have eluded vaccine development. Many protective bnAbs against HIV are directed toward glycan-dependent epitopes on the trimeric glycoprotein spike (Burton and Hangartner, 2016; Mascola and Haynes, 2013; West et al., 2014). Therefore, methods are needed to develop trimer immunogens for germline targeting and boosting of glycan-dependent bnAbs. Trimer immunogens should be stabilized, to maximize the probability of retaining native-like conformational epitopes in vivo and to minimize the probability of eliciting non-neutralizing Abs that could potentially detract from priming or boosting the targeted bnAb responses.

Here, we (1) developed a mammalian-cell-surface-display directed-evolution method for optimization of multimeric antigens bearing human glycans; (2) engineered stabilized HIV Env trimers with affinity for both germline and mature PGT121-class glycan-dependent bnAbs; (3) showed by crystallography and EM that these trimers maintain native-like conformations; (4) demonstrated that germline-targeting trimers multimerized on liposomes potently activate PGT121 germline and mature B cells ex vivo; and (5) showed that soluble germline-targeting trimers can prime PGT121-class responses in vivo, in a PGT121 inferred-germline knockin mouse. Our data indicate that 11MUT$_B$

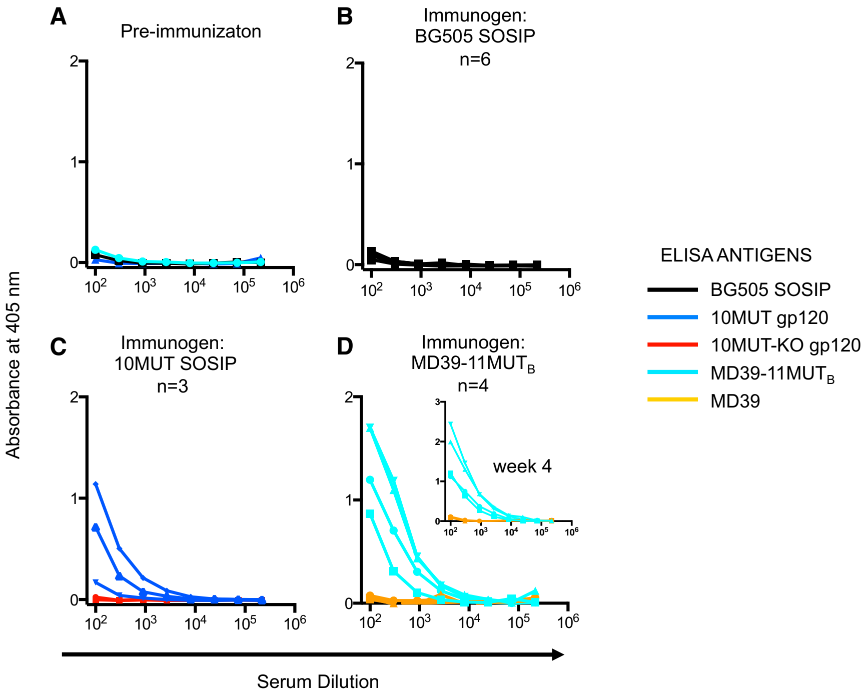

Figure 6. Serum ELISA Binding Specificity of PGT121 Germline-Reverted ($GL_{CDR3rev4}$) Knockin Mice Before and After a Single Immunization with BG505 SOSIP, 10MUT SOSIP, or MD39-11MUT$_B$ SOSIP

(A) Pre-immunization sera showed no reactivity to the four antigens tested (10MUT gp120, 10MUT-KO gp120, MD39, and MD39-11MUT$_B$).

(B) Mouse sera from 2 weeks after immunization with BG505 SOSIP showed no reactivity to BG505 SOSIP.

(C) Mouse sera from 2 weeks after immunization with 10MUT SOSIP showed reactivity to 10MUT gp120 and not to 10MUT-KO gp120.

(D) Mouse sera from 2 weeks after immunization with MD39-11MUT$_B$ SOSIP showed reactivity to the immunogen and not to MD39 SOSIP; mouse sera from 4 weeks after immunization showed similar results (inset).

The number of mice used for each experiment is indicated. A duplicate experiment for (C) with two additional mice gave similar results.

trimers and trimer-liposomes are promising candidates for priming PGT121-class glycan-dependent bnAb responses in immune systems with diverse antibody repertoires, although the frequency of PGT121-class precursors in humans and the germline-targeting affinities and/or avidities necessary to prime those precursors remain to be determined.

This work could provide a more general template for HIV bnAb germline-targeting than previous work on germline-targeting for VRC01-class bnAbs directed to the CD4-binding site. VRC01-class bnAbs generally do not depend on glycans for their activity, evidenced by the fact that elimination of glycans surrounding the VRC01 epitope generally increases neutralization potency (Jardine et al., 2016b); this has led to removal of all epitope-proximal native glycans from germline-targeting candidates (Jardine et al., 2013, 2015, 2016a; McGuire et al., 2013, 2014, 2016). However, the activity of many HIV bnAbs requires engagement of one or more glycans within their epitope, and germline-targeting primes should probably retain such key glycans, as was the case here with the N332, N301, and N156 glycans. Furthermore, owing to the relative inaccessibility of the VRC01 epitope on native-like trimers, efforts to design VRC01-class germline-targeting primes have converged on strategies to increase epitope exposure by presentation on minimal domains rather than on trimers (Jardine et al., 2013, 2015, 2016a; McGuire et al., 2013, 2016), although boosting with native-like trimers is anticipated to be required to mature the response (Jardine et al., 2016b). In contrast, many bnAb proteoglycan epitopes are well exposed on native-like trimers, and some are formed only on intact trimers, making native-like trimers like those designed here the preferred platform for germline targeting. Indeed, multiple different bnAbs could potentially be primed with a single trimer harboring multiple germline-targeting epitopes.

Because germline-targeting vaccine design requires developing not only the vaccine prime but also boost immunogens to mature the response in order to elicit bnAbs, we developed both a stabilized native-like trimer (MD39) and a cocktail of native-like trimers (VLC) that could be employed as boosts to potentially refine and expand the breadth of responses initiated by a germline-targeting prime. However, considering that memory B cells induced by the germline-targeting prime might not be sufficiently mutated to be boosted by a native-like trimer, intermediate boosts might be needed to mature the response prior to native-like boosts. In the process of developing PGT121-class germline-targeting immunogens, we created design intermediates with increasing levels of epitope modification between wild-type and germline-targeting trimers. These molecules are candidate boost immunogens that, if used in sequence, offer directional and gradual epitope changes to guide maturation of the B cell response. We proposed seven potential sequential immunization schemes, and our analysis of affinity drops provided a ranking of those schemes. In a related paper (Escolano et al., 2016), a subset of these prime-boosting schemes were evaluated in PGT121 germline knockin mice and PGT121 mature-heavy-and-germline-light-chain knockin mice, and one such scheme was found to be effective for inducing bnAbs in both mouse models, supporting the germline-targeting vaccine design process described here and encouraging its expanded use and further improvement.

Although here we have described strategies for designing trimer immunogens with changes in the structure of an epitope in order to prime and mature an epitope-specific response, the ultimate success of these strategies might also require modification of antigenic surfaces outside the epitope of interest, to minimize boosting of off-target responses that might hinder or interfere with the desired epitope-specific response.

The approaches employed here could be adapted for immunogen design to other bnAb targets on HIV and other pathogens. The "bootstrapping" strategy of using partially mutated

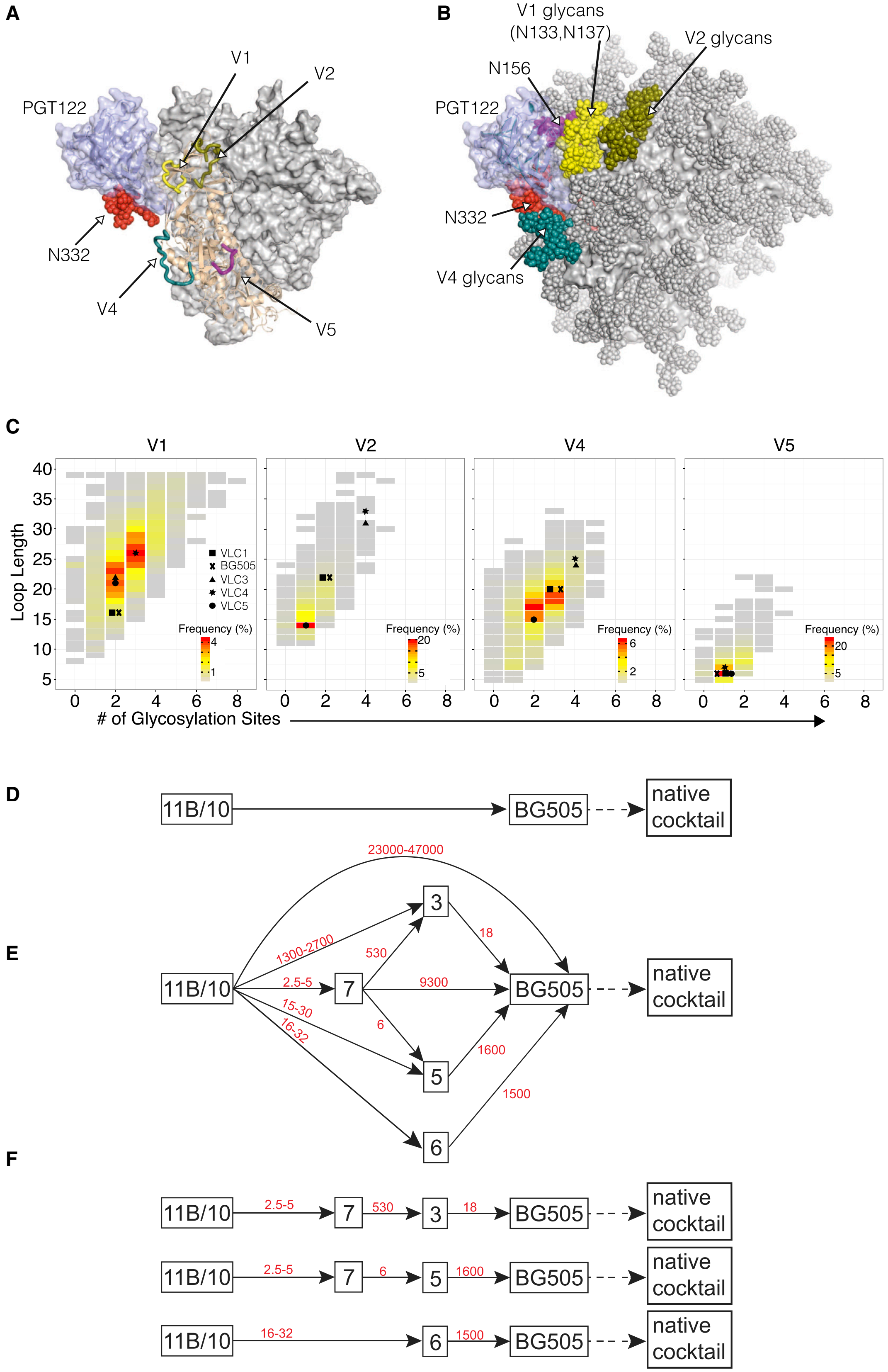

(legend on next page)

antibodies (such as GL+3 and GL+9) as initial selection agents and then using antibodies closer to germline in successive iterations could be useful for design of germline-targeting and boosting immunogens for other bnAbs, such as HIV V2 Apex glycan-dependent bnAbs (Andrabi et al., 2015; Gorman et al., 2016) or influenza virus hemagglutinin stem-directed bnAbs. Our mammalian display methods allowing directed evolution on native-like trimers should be useful in those endeavors and could also be used to stabilize monomeric or multimeric glycoprotein immunogens for diverse viral vaccines.

In summary, we have developed stabilized native-like trimer immunogens for germline-targeting and boosting of glycan-dependent PGT121-class bnAb responses against HIV. The immunogens and boosting schemes we created are candidates for human vaccine testing and further optimization, and the methods developed here are applicable to immunogen design for other epitopes and pathogens and thus are of relevance for future vaccine design.

EXPERIMENTAL PROCEDURES

DNA Gene Synthesis and Protein Production

Genes were synthesized by GenScript. gp120s, gp140s, antigen binding fragments (Fabs), and IgGs were expressed in 293 cells and purified as described in the Supplemental Experimental Procedures.

Library Assembly

The BG505 SOSIP whole-gene saturation mutagenesis and “rare” amino acid libraries were synthesized by Integrated DNA Technologies and GenScript, respectively. Libraries for germline targeting were created by error-prone PCR (GeneMorph II, Agilent), site-directed mutagenesis (QuikChange, Agilent) or two-step assembly PCR of degenerate primers with the Q5 High-Fidelity DNA Polymerase (New England Biolabs) and cloned into a modified version of the gateway cloning entry vector pENTR/D-TOPO (Ota et al., 2012) with the circular polymerase extension cloning (CPEC) method (Quan and Tian, 2014) or Gibson Assembly (New England Biolabs), according to the manufacturer’s instructions. All libraries were then transferred to the lentiviral vector pLenti CMVTRE3G puro Dest (Ota et al., 2012) with the LR Clonase II enzyme mix (Thermo Scientific).

Lentivirus Production and Stable Cell Generation

293T cells cultured in Advanced DMEM (GIBCO) supplemented with 5% fetal calf serum, GlutaMAX (GIBCO), 2-mercaptoethanol (GIBCO), and Antibiotic-Antimycotic (GIBCO) were co-transfected with 10.8 μg pLenti CMVTRE3G puro Dest gene library, 7.0 μg psPAX2, and 3.8 μg pMD2.G, as previously described (Salmon and Trono, 2007). 293T cells stably expressing rtTA3G from the pLenti CMV rtTA3G Blast vector (Ota et al., 2012) were transduced at low MOI (< 0.1) in a T75 or T225 flask in the presence of 10 μg/mL blasticidin and, after 24 hr, were transferred to medium supplemented with 2 μg/mL puromycin.

Cell Surface Expression and FACS

293T cells containing the stable library were induced with doxycycline (1 μg/mL) and harvested the next day in fluorescence-activated cell sorting (FACS) buffer (HBSS, 1 mM EDTA, 0.5% BSA). Cells containing BG505-SOSIP libraries were transfected with furin 24 hr prior to induction. Cells were stained with IgGs or Fabs for ~30 min, washed with FACS buffer, and then stained with fluorescein isothiocyanate (FITC)-labeled α-cMyc (Immunology Consultants Laboratory). IgGs were labeled with phycoerythrin (PE)-conjugated α-human IgG (Sigma), Fabs containing HA epitope tags (PGT145, PGT151, and PG16) were labeled with α-HA-PE (Miltenyi Biotec), and Fabs containing V5 epitope tags (B6 and 4025) were labeled with α-V5-FITC (GeneTex). Cells were sorted on a BD Influx (BD Biosciences) FACS sorter. Approximately 2×10^5 double positive cells were collected and expanded for approximately one week in the presence of puromycin and blasticidin before the next round of enrichment. Once the desired population had been obtained, chromosomal DNA was extracted from the cell culture with the GenElute Mammalian Genomic DNA Miniprep Kit (Sigma). The gp120 or gp140 gene was PCR amplified from the genomic DNA and inserted back into the pENTR vector via CPEC cloning or Gibson Assembly and transformed into TOP10 competent cells (Invitrogen); colonies were sequenced at Genewiz.

Next-Generation Sequencing

Sequencing and bioinformatic analysis of the BG505-SOSIP whole-gene saturation mutagenesis libraries were done essentially as described previously (Jardine et al., 2016a).

Trimer-Conjugated Liposome Synthesis and Characterization

Unilamellar liposomes comprised of DSPC:cholesterol:DGS-NTA(Ni) lipids in a 66.5:28.5:5 mole ratio were synthesized by lipid film rehydration and membrane extrusion, followed by post-synthesis binding of 6xHis-tagged trimer for 2 hr at 4°C. Unconjugated trimer was removed by size exclusion chromatography. Total conjugated trimer was quantified by ELISA in the presence of 1% triton-X and 100 mM imidazole to fully disrupt liposomes and Ni-6xHis interactions, respectively, for uninhibited detection via an α-6xHis antibody. Antigenic profiles were determined by ELISA on intact liposomes.

Ca^{2+}-Flux Measurements and Immunizations

Details about Ca^{2+}-flux assays and mouse immunizations can be found in the Supplemental Experimental Procedures and in Escolano et al., 2016.

Negative-Stain EM

Purified SOSIP trimers were analyzed by negative-stain EM with a protocol adapted from de Taeye et al., 2016.

Figure 7. Sequential Boosting Schemes Employing a Native-Like Trimer Cocktail and Germline-Targeting Design Intermediates

(A) Side view of a single PGT122 Fab (light blue cartoon and semi-transparent surface) bound to the BG505 SOSIP native-like gp140 trimer, based on PDB: 4NCO. The PGT122-bound gp140 subunit is shown in wheat-colored cartoon; the V1, V2, V4, and V5 variable loops on that subunit, modeled wherever missing in the crystal structure, are shown in yellow (V1), olive (V2), teal (V4), and magenta (V5); the N332 glycan is shown as red spheres; and the two other gp140 subunits are shown as gray surfaces.

(B) Same model as in (A), except that glycosylation sites on the trimer have been decorated with $Man_8GlcNAc_2$ glycans shown as spheres (V1 glycans, red; V2 glycans, olive; V4 glycans, teal; N156 glycan, magenta; N332 glycan, red; all other glycans, gray), and all trimer subunits are shown as gray surfaces.

(C) 2D histogram of variable loop (V1, V2, V4, and V5) length and number of glycosylation sites among 3,897 unique HIV Env sequences isolated from infected individuals and obtained from http://www.hiv.lanl.govcontent/index. Frequency is indicated by the color scale shown for each loop. The length and number of glycosylation sites for each loop of the native-like VLC trimers are indicated. Further elaboration of this cocktail could include accounting for sequence variation at non-variable-loop positions within the N332-epitope region (Figure S7C).

(D) Basic scheme in which a germline-targeting prime (10MUT or $11MUT_B$) is boosted by a native-like trimer (BG505) and then by a cocktail of native-like trimers.

(E) Diagram illustrating seven boosting schemes employing germline-targeting design intermediates (7MUT, 6MUT, 5MUT, and 3MUT) as boosts after a germline-targeting prime and before a native-like trimer; the scheme in (A) is included for reference. Relative affinity drops (in fold affinity decrease) for each boost, computed from Figure 1B as described in the text, are indicated as red numbers.

(F) Linear diagrams of three of the best boosting schemes as ranked by favoring those with the smallest maximum affinity drop.

DSC and SPR Methods

Differential scanning calorimetry (DSC) and surface plasmon resonance (SPR) methods are described in the Supplemental Experimental Procedures.

Crystallization and Data Collection

Description of crystallization, data collection, and refinement can be found in the Supplemental Experimental Procedures. Statistics for data collection and final refinement are listed in Table S2.

ACCESSION NUMBERS

The accession number for the coordinates and structure factors for MD39-10MUT$_A$ in complex with 35O22 and PGT124 is PDB: 5T3S.

SUPPLEMENTAL INFORMATION

Supplemental Information includes Supplemental Experimental Procedures, seven figures, and three tables and can be found with this article online at http://dx.doi.org/10.1016/j.immuni.2016.08.016.

AUTHOR CONTRIBUTIONS

J.M.S., D.W.K., and W.R.S. conceived the immunogen design strategies, developed mammalian display, and designed immunogens and boosting schemes. L.E.M., B.B., and D.R.B. assisted with mammalian display directed evolution and provided intermediate mutated Abs for selection agents. S.M. and D.W.K. carried out loop and glycan modeling. A.E. and M.C.N. provided the plasmid for GL$_{CDR3\text{-}rev4}$. Y.A., M.K., E.G., M.J., and D.W.K. produced immunogens and Abs. O.K., X.H., T.S., L.E.M., J.M.S., and D.W.K. characterized immunogens and Abs with biophysical analysis methods. R.L.S., F.G., I.A.W., G.O., and A.B.W. characterized immunogens and Abs with structural analysis methods. T.T., D.J.I., D.W.K., and W.R.S. conceived trimer-liposome immunogens. T.T., A.M., D.S.Y., and D.J.I. developed and characterized trimer liposomes by using biophysical and structural analysis. N.T.F., A.D.G., A.E., and M.C.N. created and characterized PGT121 GL knockin mice, encouraging germline-targeting efforts. P.D., M.C.N., J.M.S., T.T., D.J.I., D.W.K., W.R.S planned ex vivo B cell activation studies. P.D., C.T.M., and M.C.N. performed and analyzed ex vivo B cell activation studies. A.E. and M.C.N. planned, performed, and analyzed immunization studies. J.M.S., D.W.K., and W.R.S. wrote the paper. M.C.N., D.J.I., D.R.B., I.A.W., A.B.W., T.T., R.L.S., L.E.M., G.O., X.H., O.K., and T.S. helped write the paper.

ACKNOWLEDGMENTS

We thank Takayuki Ota and David Nemazee for providing lentivirus protocols and reagents and Leo Stamatatos and Jean-Philippe Julien for comments on the manuscript. This work was supported by National Institute of Allergy and Infectious Diseases grants CHAVI-ID 1UM1AI100663 (to W.R.S., D.J.I., M.C.N., I.A.W., D.R.B., and A.B.W.), P01 AI110657 (to I.A.W.), and R01 AI084817 (to I.A.W.) and by the Ragon Institute of MGH, MIT, and Harvard (to D.R.B., D.J.I., and W.R.S.). This work was also partially funded by the International AIDS Vaccine Initiative (IAVI) with the generous support of the United States Agency for International Development, the Ministry of Foreign Affairs of the Netherlands, and the Bill & Melinda Gates Foundation; a full list of IAVI donors is available at www.iavi.org (W.R.S., D.R.B, A.B.W., and I.A.W.). Portions of this research were carried out at the Stanford Synchrotron Radiation Lightsource (SSRL), a directorate of the SLAC National Accelerator Laboratory, and an Office of Science user facility operated for the U.S. Department of Energy (DOE) Office of Science by Stanford University. The SSRL Structural Molecular Biology Program is supported by the DOE Office of Biological and Environmental Research, the NIH, and the National Institute of General Medical Sciences (including P41GM103393). The Scripps Research Institute and IAVI have filed for a patent related to immunogens in this manuscript, with inventors J.M.S., D.W.K., X.H., S.M., and W.R.S. Materials and information will be provided under a material transfer agreement. W.R.S. is a co-founder and stockholder in Compuvax, which has programs in non-HIV vaccine design that might benefit indirectly from this research.

Received: May 26, 2016
Revised: July 28, 2016
Accepted: August 25, 2016
Published: September 8, 2016

REFERENCES

Andrabi, R., Voss, J.E., Liang, C.H., Briney, B., McCoy, L.E., Wu, C.Y., Wong, C.H., Poignard, P., and Burton, D.R. (2015). Identification of Common Features in Prototype Broadly Neutralizing Antibodies to HIV Envelope V2 Apex to Facilitate Vaccine Design. Immunity *43*, 959–973.

Barbas, C.F., 3rd, Björling, E., Chiodi, F., Dunlop, N., Cababa, D., Jones, T.M., Zebedee, S.L., Persson, M.A., Nara, P.L., Norrby, E., et al. (1992). Recombinant human Fab fragments neutralize human type 1 immunodeficiency virus in vitro. Proc. Natl. Acad. Sci. USA *89*, 9339–9343.

Barouch, D.H., Whitney, J.B., Moldt, B., Klein, F., Oliveira, T.Y., Liu, J., Stephenson, K.E., Chang, H.W., Shekhar, K., Gupta, S., et al. (2013). Therapeutic efficacy of potent neutralizing HIV-1-specific monoclonal antibodies in SHIV-infected rhesus monkeys. Nature *503*, 224–228.

Burton, D.R., and Hangartner, L. (2016). Broadly Neutralizing Antibodies to HIV and Their Role in Vaccine Design. Annu. Rev. Immunol. *34*, 635–659.

Chen, K.C., Wu, C.H., Chang, C.Y., Lu, W.C., Tseng, Q., Prijovich, Z.M., Schechinger, W., Liaw, Y.C., Leu, Y.L., and Roffler, S.R. (2008). Directed evolution of a lysosomal enzyme with enhanced activity at neutral pH by mammalian cell-surface display. Chem. Biol. *15*, 1277–1286.

de Taeye, S.W., Moore, J.P., and Sanders, R.W. (2016). HIV-1 Envelope Trimer Design and Immunization Strategies To Induce Broadly Neutralizing Antibodies. Trends Immunol. *37*, 221–232.

Dimitrov, D.S. (2010). Therapeutic antibodies, vaccines and antibodyomes. MAbs *2*, 347–356.

Doria-Rose, N.A., Schramm, C.A., Gorman, J., Moore, P.L., Bhiman, J.N., DeKosky, B.J., Ernandes, M.J., Georgiev, I.S., Kim, H.J., Pancera, M., et al.; NISC Comparative Sequencing Program (2014). Developmental pathway for potent V1V2-directed HIV-neutralizing antibodies. Nature *509*, 55–62.

Dosenovic, P., von Boehmer, L., Escolano, A., Jardine, J., Freund, N.T., Gitlin, A.D., McGuire, A.T., Kulp, D.W., Oliveira, T., Scharf, L., et al. (2015). Immunization for HIV-1 Broadly Neutralizing Antibodies in Human Ig Knockin Mice. Cell *161*, 1505–1515.

Escolano, A., Steichen, J.M., Dosenovic, P., Kulp, D.W., Golijanin, J., Sok, D., Freund, N.T., Gitlin, A.D., Oliveira, T., Araki, T., et al. (2016). Sequential immunization elicits broadly neutralizing anti-HIV-1 antibodies in Ig knockin mice. Cell *166*.

Falkowska, E., Le, K.M., Ramos, A., Doores, K.J., Lee, J.H., Blattner, C., Ramirez, A., Derking, R., van Gils, M.J., Liang, C.H., et al. (2014). Broadly neutralizing HIV antibodies define a glycan-dependent epitope on the prefusion conformation of gp41 on cleaved envelope trimers. Immunity *40*, 657–668.

Garces, F., Sok, D., Kong, L., McBride, R., Kim, H.J., Saye-Francisco, K.F., Julien, J.P., Hua, Y., Cupo, A., Moore, J.P., et al. (2014). Structural evolution of glycan recognition by a family of potent HIV antibodies. Cell *159*, 69–79.

Garces, F., Lee, J.H., de Val, N., de la Pena, A.T., Kong, L., Puchades, C., Hua, Y., Stanfield, R.L., Burton, D.R., Moore, J.P., et al. (2015). Affinity Maturation of a Potent Family of HIV Antibodies Is Primarily Focused on Accommodating or Avoiding Glycans. Immunity *43*, 1053–1063.

Georgiev, I.S., Rudicell, R.S., Saunders, K.O., Shi, W., Kirys, T., McKee, K., O'Dell, S., Chuang, G.Y., Yang, Z.Y., Ofek, G., et al. (2014). Antibodies VRC01 and 10E8 neutralize HIV-1 with high breadth and potency even with Ig-framework regions substantially reverted to germline. J. Immunol. *192*, 1100–1106.

Gorman, J., Soto, C., Yang, M.M., Davenport, T.M., Guttman, M., Bailer, R.T., Chambers, M., Chuang, G.Y., DeKosky, B.J., Doria-Rose, N.A., et al.; NISC Comparative Sequencing Program (2016). Structures of HIV-1 Env V1V2 with broadly neutralizing antibodies reveal commonalities that enable vaccine design. Nat. Struct. Mol. Biol. *23*, 81–90.

Gorny, M.K., Sampson, J., Li, H., Jiang, X., Totrov, M., Wang, X.H., Williams, C., O'Neal, T., Volsky, B., Li, L., et al. (2011). Human anti-V3 HIV-1 monoclonal antibodies encoded by the VH5-51/VL lambda genes define a conserved antigenic structure. PLoS ONE *6*, e27780.

Haynes, B.F., Fleming, J., St Clair, E.W., Katinger, H., Stiegler, G., Kunert, R., Robinson, J., Scearce, R.M., Plonk, K., Staats, H.F., et al. (2005). Cardiolipin polyspecific autoreactivity in two broadly neutralizing HIV-1 antibodies. Science *308*, 1906–1908.

Haynes, B.F., Kelsoe, G., Harrison, S.C., and Kepler, T.B. (2012). B-cell-lineage immunogen design in vaccine development with HIV-1 as a case study. Nat. Biotechnol. *30*, 423–433.

Hoot, S., McGuire, A.T., Cohen, K.W., Strong, R.K., Hangartner, L., Klein, F., Diskin, R., Scheid, J.F., Sather, D.N., Burton, D.R., and Stamatatos, L. (2013). Recombinant HIV envelope proteins fail to engage germline versions of anti-CD4bs bNAbs. PLoS Pathog. *9*, e1003106.

Hu, J.K., Crampton, J.C., Cupo, A., Ketas, T., van Gils, M.J., Sliepen, K., de Taeye, S.W., Sok, D., Ozorowski, G., Deresa, I., et al. (2015). Murine Antibody Responses to Cleaved Soluble HIV-1 Envelope Trimers Are Highly Restricted in Specificity. J. Virol. *89*, 10383–10398.

Jardine, J., Julien, J.P., Menis, S., Ota, T., Kalyuzhniy, O., McGuire, A., Sok, D., Huang, P.S., MacPherson, S., Jones, M., et al. (2013). Rational HIV immunogen design to target specific germline B cell receptors. Science *340*, 711–716.

Jardine, J.G., Ota, T., Sok, D., Pauthner, M., Kulp, D.W., Kalyuzhniy, O., Skog, P.D., Thinnes, T.C., Bhullar, D., Briney, B., et al. (2015). HIV-1 VACCINES. Priming a broadly neutralizing antibody response to HIV-1 using a germline-targeting immunogen. Science *349*, 156–161.

Jardine, J.G., Kulp, D.W., Havenar-Daughton, C., Sarkar, A., Briney, B., Sok, D., Sesterhenn, F., Ereño-Orbea, J., Kalyuzhniy, O., Deresa, I., et al. (2016a). HIV-1 broadly neutralizing antibody precursor B cells revealed by germline-targeting immunogen. Science *351*, 1458–1463.

Jardine, J., Sok, D., Julien, J.P., Briney, B., Sarkar, A., Adachi, Y., Dewanji, D., Hsueh, J., Jones, M., Kalyuzhniy, O., et al. (2016b). Minimally mutated HIV-1 broadly neutralizing antibodies to guide reductionist vaccine design. PLoS Pathogens. http://dx.doi.org/10.1371/journal.ppat.1005815.

Julien, J.P., Cupo, A., Sok, D., Stanfield, R.L., Lyumkis, D., Deller, M.C., Klasse, P.J., Burton, D.R., Sanders, R.W., Moore, J.P., et al. (2013). Crystal structure of a soluble cleaved HIV-1 envelope trimer. Science *342*, 1477–1483.

Kepler, T.B., Liao, H.X., Alam, S.M., Bhaskarabhatla, R., Zhang, R., Yandava, C., Stewart, S., Anasti, K., Kelsoe, G., Parks, R., et al. (2014). Immunoglobulin gene insertions and deletions in the affinity maturation of HIV-1 broadly reactive neutralizing antibodies. Cell Host Microbe *16*, 304–313.

Klein, F., Diskin, R., Scheid, J.F., Gaebler, C., Mouquet, H., Georgiev, I.S., Pancera, M., Zhou, T., Incesu, R.B., Fu, B.Z., et al. (2013a). Somatic mutations of the immunoglobulin framework are generally required for broad and potent HIV-1 neutralization. Cell *153*, 126–138.

Klein, F., Mouquet, H., Dosenovic, P., Scheid, J.F., Scharf, L., and Nussenzweig, M.C. (2013b). Antibodies in HIV-1 vaccine development and therapy. Science *341*, 1199–1204.

Kong, R., Xu, K., Zhou, T., Acharya, P., Lemmin, T., Liu, K., Ozorowski, G., Soto, C., Taft, J.D., Bailer, R.T., et al. (2016). Fusion peptide of HIV-1 as a site of vulnerability to neutralizing antibody. Science *352*, 828–833.

Kwon, Y.D., Pancera, M., Acharya, P., Georgiev, I.S., Crooks, E.T., Gorman, J., Joyce, M.G., Guttman, M., Ma, X., Narpala, S., et al. (2015). Crystal structure, conformational fixation and entry-related interactions of mature ligand-free HIV-1 Env. Nat. Struct. Mol. Biol. *22*, 522–531.

Landais, E., Huang, X., Havenar-Daughton, C., Murrell, B., Price, M.A., Wickramasinghe, L., Ramos, A., Bian, C.B., Simek, M., Allen, S., et al. (2016). Broadly Neutralizing Antibody Responses in a Large Longitudinal Sub-Saharan HIV Primary Infection Cohort. PLoS Pathog. *12*, e1005369.

Liao, H.X., Lynch, R., Zhou, T., Gao, F., Alam, S.M., Boyd, S.D., Fire, A.Z., Roskin, K.M., Schramm, C.A., Zhang, Z., et al.; NISC Comparative Sequencing Program (2013). Co-evolution of a broadly neutralizing HIV-1 antibody and founder virus. Nature *496*, 469–476.

Lyumkis, D., Julien, J.P., de Val, N., Cupo, A., Potter, C.S., Klasse, P.J., Burton, D.R., Sanders, R.W., Moore, J.P., Carragher, B., et al. (2013). Cryo-EM structure of a fully glycosylated soluble cleaved HIV-1 envelope trimer. Science *342*, 1484–1490.

Mascola, J.R., and Haynes, B.F. (2013). HIV-1 neutralizing antibodies: understanding nature's pathways. Immunol. Rev. *254*, 225–244.

McGuire, A.T., Hoot, S., Dreyer, A.M., Lippy, A., Stuart, A., Cohen, K.W., Jardine, J., Menis, S., Scheid, J.F., West, A.P., et al. (2013). Engineering HIV envelope protein to activate germline B cell receptors of broadly neutralizing anti-CD4 binding site antibodies. J. Exp. Med. *210*, 655–663.

McGuire, A.T., Dreyer, A.M., Carbonetti, S., Lippy, A., Glenn, J., Scheid, J.F., Mouquet, H., and Stamatatos, L. (2014). HIV antibodies. Antigen modification regulates competition of broad and narrow neutralizing HIV antibodies. Science *346*, 1380–1383.

McGuire, A.T., Gray, M.D., Dosenovic, P., Gitlin, A.D., Freund, N.T., Petersen, J., Correnti, C., Johnsen, W., Kegel, R., Stuart, A.B., et al. (2016). Specifically modified Env immunogens activate B-cell precursors of broadly neutralizing HIV-1 antibodies in transgenic mice. Nat. Commun. *7*, 10618.

Moldt, B., Rakasz, E.G., Schultz, N., Chan-Hui, P.Y., Swiderek, K., Weisgrau, K.L., Piaskowski, S.M., Bergman, Z., Watkins, D.I., Poignard, P., and Burton, D.R. (2012). Highly potent HIV-specific antibody neutralization in vitro translates into effective protection against mucosal SHIV challenge in vivo. Proc. Natl. Acad. Sci. USA *109*, 18921–18925.

Mouquet, H., Scheid, J.F., Zoller, M.J., Krogsgaard, M., Ott, R.G., Shukair, S., Artyomov, M.N., Pietzsch, J., Connors, M., Pereyra, F., et al. (2010). Polyreactivity increases the apparent affinity of anti-HIV antibodies by heteroligation. Nature *467*, 591–595.

Mouquet, H., Scharf, L., Euler, Z., Liu, Y., Eden, C., Scheid, J.F., Halper-Stromberg, A., Gnanapragasam, P.N., Spencer, D.I., Seaman, M.S., et al. (2012). Complex-type N-glycan recognition by potent broadly neutralizing HIV antibodies. Proc. Natl. Acad. Sci. USA *109*, E3268–E3277.

Ota, T., Doyle-Cooper, C., Cooper, A.B., Huber, M., Falkowska, E., Doores, K.J., Hangartner, L., Le, K., Sok, D., Jardine, J., et al. (2012). Anti-HIV B Cell lines as candidate vaccine biosensors. J. Immunol. *189*, 4816–4824.

Pancera, M., McLellan, J.S., Wu, X., Zhu, J., Changela, A., Schmidt, S.D., Yang, Y., Zhou, T., Phogat, S., Mascola, J.R., and Kwong, P.D. (2010). Crystal structure of PG16 and chimeric dissection with somatically related PG9: structure-function analysis of two quaternary-specific antibodies that effectively neutralize HIV-1. J. Virol. *84*, 8098–8110.

Pancera, M., Zhou, T., Druz, A., Georgiev, I.S., Soto, C., Gorman, J., Huang, J., Acharya, P., Chuang, G.Y., Ofek, G., et al. (2014). Structure and immune recognition of trimeric pre-fusion HIV-1 Env. Nature *514*, 455–461.

Pugach, P., Ozorowski, G., Cupo, A., Ringe, R., Yasmeen, A., de Val, N., Derking, R., Kim, H.J., Korzun, J., Golabek, M., et al. (2015). A native-like SOSIP.664 trimer based on an HIV-1 subtype B env gene. J. Virol. *89*, 3380–3395.

Quan, J., and Tian, J. (2014). Circular polymerase extension cloning. Methods Mol. Biol. *1116*, 103–117.

Salmon, P., and Trono, D. (2007). Production and titration of lentiviral vectors. In Current Protocols in Human Genetics (John Wiley & Sons), unit 12.10.

Sanders, R.W., Derking, R., Cupo, A., Julien, J.P., Yasmeen, A., de Val, N., Kim, H.J., Blattner, C., de la Peña, A.T., Korzun, J., et al. (2013). A next-generation cleaved, soluble HIV-1 Env trimer, BG505 SOSIP.664 gp140, expresses multiple epitopes for broadly neutralizing but not non-neutralizing antibodies. PLoS Pathog. *9*, e1003618.

Sanders, R.W., van Gils, M.J., Derking, R., Sok, D., Ketas, T.J., Burger, J.A., Ozorowski, G., Cupo, A., Simonich, C., Goo, L., et al. (2015). HIV-1 VACCINES. HIV-1 neutralizing antibodies induced by native-like envelope trimers. Science *349*, aac4223.

Scharf, L., Wang, H., Gao, H., Chen, S., McDowall, A.W., and Bjorkman, P.J. (2015). Broadly Neutralizing Antibody 8ANC195 Recognizes Closed and Open States of HIV-1 Env. Cell *162*, 1379–1390.

Scheid, J.F., Mouquet, H., Feldhahn, N., Seaman, M.S., Velinzon, K., Pietzsch, J., Ott, R.G., Anthony, R.M., Zebroski, H., Hurley, A., et al. (2009). Broad

diversity of neutralizing antibodies isolated from memory B cells in HIV-infected individuals. Nature *458*, 636–640.

Scheid, J.F., Mouquet, H., Ueberheide, B., Diskin, R., Klein, F., Oliveira, T.Y., Pietzsch, J., Fenyo, D., Abadir, A., Velinzon, K., et al. (2011). Sequence and structural convergence of broad and potent HIV antibodies that mimic CD4 binding. Science *333*, 1633–1637.

Shingai, M., Nishimura, Y., Klein, F., Mouquet, H., Donau, O.K., Plishka, R., Buckler-White, A., Seaman, M., Piatak, M., Jr., Lifson, J.D., et al. (2013). Antibody-mediated immunotherapy of macaques chronically infected with SHIV suppresses viraemia. Nature *503*, 277–280.

Shingai, M., Donau, O.K., Plishka, R.J., Buckler-White, A., Mascola, J.R., Nabel, G.J., Nason, M.C., Montefiori, D., Moldt, B., Poignard, P., et al. (2014). Passive transfer of modest titers of potent and broadly neutralizing anti-HIV monoclonal antibodies block SHIV infection in macaques. J. Exp. Med. *211*, 2061–2074.

Sok, D., Laserson, U., Laserson, J., Liu, Y., Vigneault, F., Julien, J.P., Briney, B., Ramos, A., Saye, K.F., Le, K., et al. (2013). The effects of somatic hypermutation on neutralization and binding in the PGT121 family of broadly neutralizing HIV antibodies. PLoS Pathog. *9*, e1003754.

Walker, L.M., Phogat, S.K., Chan-Hui, P.Y., Wagner, D., Phung, P., Goss, J.L., Wrin, T., Simek, M.D., Fling, S., Mitcham, J.L., et al.; Protocol G Principal Investigators (2009). Broad and potent neutralizing antibodies from an African donor reveal a new HIV-1 vaccine target. Science *326*, 285–289.

Walker, L.M., Huber, M., Doores, K.J., Falkowska, E., Pejchal, R., Julien, J.P., Wang, S.K., Ramos, A., Chan-Hui, P.Y., Moyle, M., et al.; Protocol G Principal Investigators (2011). Broad neutralization coverage of HIV by multiple highly potent antibodies. Nature *477*, 466–470.

West, A.P., Jr., Scharf, L., Scheid, J.F., Klein, F., Bjorkman, P.J., and Nussenzweig, M.C. (2014). Structural insights on the role of antibodies in HIV-1 vaccine and therapy. Cell *156*, 633–648.

Wu, X., Zhou, T., Zhu, J., Zhang, B., Georgiev, I., Wang, C., Chen, X., Longo, N.S., Louder, M., McKee, K., et al.; NISC Comparative Sequencing Program (2011). Focused evolution of HIV-1 neutralizing antibodies revealed by structures and deep sequencing. Science *333*, 1593–1602.

Xiao, X., Chen, W., Feng, Y., Zhu, Z., Prabakaran, P., Wang, Y., Zhang, M.Y., Longo, N.S., and Dimitrov, D.S. (2009). Germline-like predecessors of broadly neutralizing antibodies lack measurable binding to HIV-1 envelope glycoproteins: implications for evasion of immune responses and design of vaccine immunogens. Biochem. Biophys. Res. Commun. *390*, 404–409.

Zhou, T., Georgiev, I., Wu, X., Yang, Z.Y., Dai, K., Finzi, A., Kwon, Y.D., Scheid, J.F., Shi, W., Xu, L., et al. (2010). Structural basis for broad and potent neutralization of HIV-1 by antibody VRC01. Science *329*, 811–817.

Immunity, Volume *45*

Supplemental Information

HIV Vaccine Design to Target Germline Precursors of Glycan-Dependent Broadly Neutralizing Antibodies

Jon M. Steichen, Daniel W. Kulp, Talar Tokatlian, Amelia Escolano, Pia Dosenovic, Robyn L. Stanfield, Laura E. McCoy, Gabriel Ozorowski, Xiaozhen Hu, Oleksandr Kalyuzhniy, Bryan Briney, Torben Schiffner, Fernando Garces, Natalia T. Freund, Alexander D. Gitlin, Sergey Menis, Erik Georgeson, Michael Kubitz, Yumiko Adachi, Meaghan Jones, Andrew A. Mutafyan, Dong Soo Yun, Christian T. Mayer, Andrew B. Ward, Dennis R. Burton, Ian A. Wilson, Darrell J. Irvine, Michel C. Nussenzweig, and William R. Schief

Figure S1

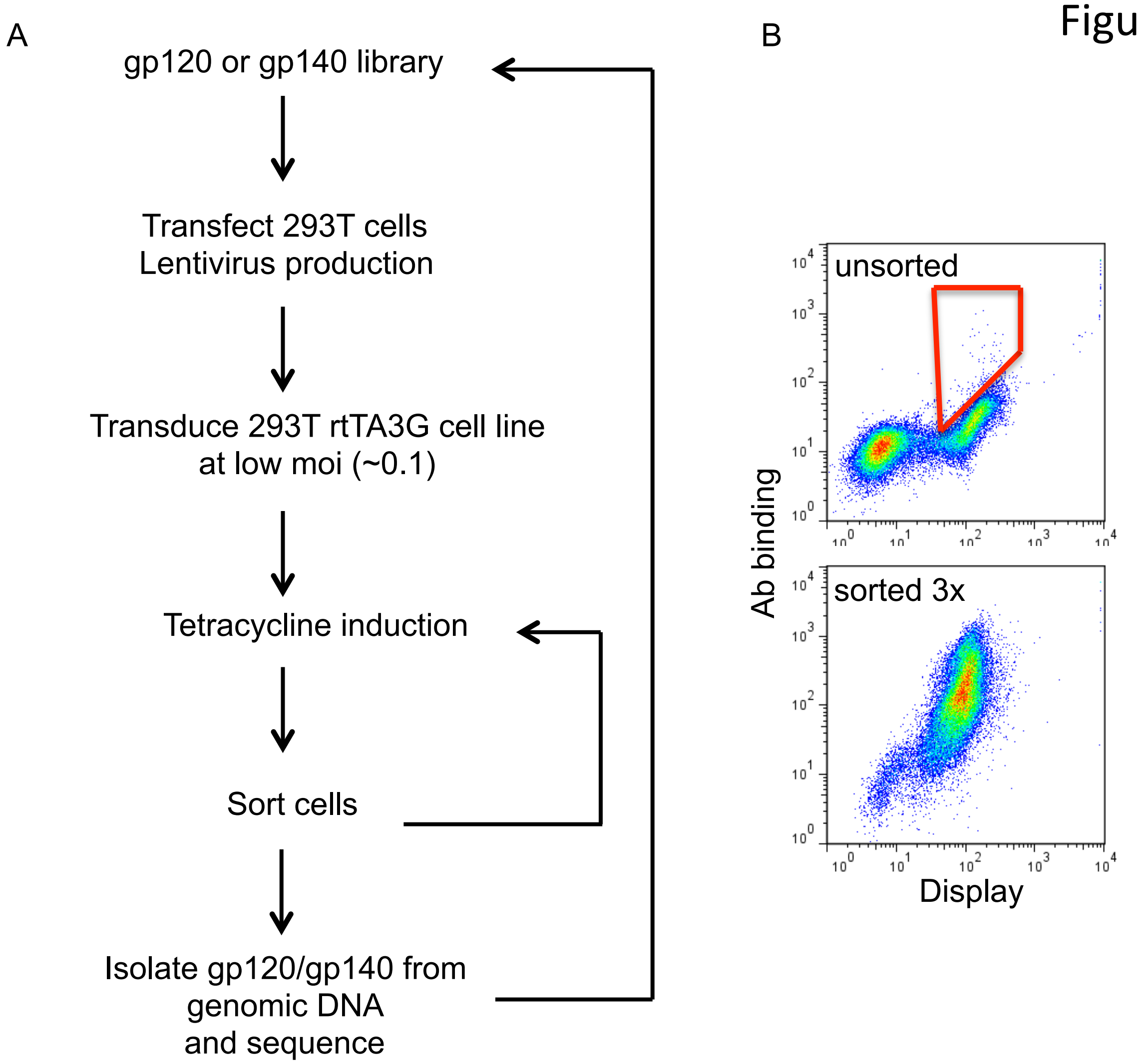

C

BG505-SOSIP.664_PDGFR_TM
MDAMKRGLCCVLLLCGAVFVSPSQEIHARFRRGARAENLWVTVYYGVPVWKDAETTLFCASDAKAYETEKHNVWATHACVPTDPNPQEIHLENVTEEFN
MWKNNMVEQMHTDIISLWDQSLKPCVKLTPLCVTLQCTNVTNNITDDMRGELKNCSFNMTTELRDKKQKVYSLFYRLDVVQINENQGNRSNNSNKEYRL
INCNTSAITQACPKVSFEPIPIHYCAPAGFAILKCKDKKFNGTGPCPSVSTVQCTHGIKPVVSTQLLLNGSLAEEEVMIRSENITNNAKNILVQFNTPV
QINCTRPNNNTRKSIRIGPGQAFYATGDIIGDIRQAHCNVSKATWNETLGKVVKQLRKHFGNNTIIRFANSSGGDLEVTTHSFNCGGEFFYCNTSGLFN
STWISNTSVQGSNSTGSNDSITLPCRIKQIINMWQRIGQAMYAPPIQGVIRCVSNITGLILTRDGGSTNSTTETFRPGGGDMRDNWRSELYKYKVVKIE
PLGVAPTRCKRRVVGRRRRRRAVGIGAVFLGFLGAAGSTMGAASMTLTVQARNLLSGIVQQQSNLLRAPEAQQHLLKLTVWGIKQLQARVLAVERYLRD
QQLLGIWGCSGKLICCTNVPWNSSWSNRNLSEIWDNMTWLQWDKEISNYTQIIYGLLEESQNQQEKNEQDLLALDGGGSGGSGGSEQKLISEEDLGGSG
GSGGSNAVGQDTQEVIVVPHSLPFKVVVISAILALVVLTIISLIILIMLWQKKPR

BG505-gp120_PDGFR_TM
METDTLLLWVLLLWVPGSTGDAENLWVTVYYGVPVWKDAETTLFCASDAKAYETEKHNVWATHACVPTDPNPQEIHLENVTEEFNMWKNNMVEQMHTD
IISLWDQSLKPCVKLTPLCVTLQCTNVTNNITDDMRGELKNCSFNMTTELRDKKQKVYSLFYRLDVVQINENQGNRSNNSNKEYRLINCNTSAITQAC
PKVSFEPIPIHYCAPAGFAILKCKDKKFNGTGPCPSVSTVQCTHGIKPVVSTQLLLNGSLAEEEVMIRSENITNNAKNILVQFNTPVQINCTRPNNNT
RKSIRIGPGQAFYATGDIIGDIRQAHCNVSKATWNETLGKVVKQLRKHFGNNTIIRFANSSGGDLEVTTHSFNCGGEFFYCNTSGLFNSTWISNTSVQ
GSNSTGSNDSITLPCRIKQIINMWQRIGQAMYAPPIQGVIRCVSNITGLILTRDGGSTNSTTETFRPGGGDMRDNWRSELYKYKVVKIEPLGVAPTRA
KRRVVGGGSGGSGGSEQKLISEEDLGGSGGSGGSNAVGQDTQEVIVVPHSLPFKVVVISAILALVVLTIISLIILIMLWQKKPR

Figure S2

Heavy Chain

```
                V
V4-59/D3-3/J6   QVQLQESGPGLVKPSETLSLTCTVSGGSISSYYWSWIRQPPGKGLEWIGYIYYSGSTNYNPSLKSRVTIS
GL-CDR3rev1     QVQLQESGPGLVKPSETLSLTCTVSGGSISSYYWSWIRQPPGKGLEWIGYIYYSGSTNYNPSLKSRVTIS
GL-CDR3rev2     QVQLQESGPGLVKPSETLSLTCTVSGGSISSYYWSWIRQPPGKGLEWIGYIYYSGSTNYNPSLKSRVTIS
GL-CDR3rev3     QVQLQESGPGLVKPSETLSLTCTVSGGSISSYYWSWIRQPPGKGLEWIGYIYYSGSTNYNPSLKSRVTIS
GL-CDR3rev4     QVQLQESGPGLVKPSETLSLTCTVSGGSISSYYWSWIRQPPGKGLEWIGYIYYSGSTNYNPSLKSRVTIS
GL-CDR3rev5     QVQLQESGPGLVKPSETLSLTCTVSGGSISSYYWSWIRQPPGKGLEWIGYIYYSGSTNYNPSLKSRVTIS
GL+3            QVQLQESGPGLVKPSETLSLTCTVSGGSISSYYWSWIRQPPGKGLEWIGYIYYSGSTNYNPSLKSRVTIS
GL-CDR3mat      QVQLQESGPGLVKPSETLSLTCTVSGGSISSYYWSWIRQPPGKGLEWIGYIYYSGSTNYNPSLKSRVTIS
GL+9            QVQLQESGPGLVKPSETLSLTCTVSGGSISSYYWSWIRQPPGKGLEWIGYIYYSGSTNYNPSLKSRVTIS
PGT121          QMQLQESGPGLVKPSETLSLTCSVSGASISDSYWSWIRRSPGKGLEWIGYVHKSGDTNYSPSLKSRVNLS

                                                  D          J
V4-59/D3-3/J6   VDTSKNQFSLKLSSVTAADTAVYYCAR----ITIFGVVII--YYYYYYMDVWGKGTTVTVSS
GL-CDR3rev1     VDTSKNQFSLKLSSVTAADTAVYYCARTLHGITIFGVVAFKEYYYYYYMDVWGKGTTVTVSS
GL-CDR3rev2     VDTSKNQFSLKLSSVTAADTAVYYCARTLHGITIFGVVIIKEYYYYYYMDVWGKGTTVTVSS
GL-CDR3rev3     VDTSKNQFSLKLSSVTAADTAVYYCARTQQGKRIYGVVSFGEYYYYYYMDVWGKGTTVTVSS
GL-CDR3rev4     VDTSKNQFSLKLSSVTAADTAVYYCARTQQGKRIYGVVSFGDYYYYYYMDVWGKGTTVTVSS
GL-CDR3rev5     VDTSKNQFSLKLSSVTAADTAVYYCARTLHGITIFGVVAFKEYYYYYYMDVWGKGTTVTVSS
GL+3            VDTSKNQFSLKLSSVTAADTAVYYCARTLHGRRIYGIVAFKEWFTYYYMDVWGKGTTVTVSS
GL-CDR3mat      VDTSKNQFSLKLSSVTAADTAVYYCARTLHGRRIYGIVAFKEWFTYYYMDVWGKGTTVTVSS
GL+9            VDTSKNQFSLKLSSVTAADTAVYYCARTLHGRRIYGIVAFKEWFTYYYMDVWGKGTTVTVSS
PGT121          LDTSKNQVSLSLVAATAADSGKYYCARTLHGRRIYGIVAFNEWFTYFYMDVWGNGTQVTVSS
```

Light Chain

```
                V
V3-21/J3        SYVLTQPPSVSVAPGQTARITCGGNNIGSKSVHWYQQKPGQAPVLVVYDDSDRPSGIPERFSGSNS---G
GL-CDR3rev1     SYVLTQPPSVSVAPGQTARITCGGNNIGSKSVHWYQQKPGQAPVLVVYDDSDRPSGIPERFSGSNS---G
GL-CDR3rev2     SYVLTQPPSVSVAPGQTARITCGGNNIGSKSVHWYQQKPGQAPVLVVYDDSDRPSGIPERFSGSNS---G
GL-CDR3rev3     SYVLTQPPSVSVAPGQTARITCGGNNIGSKSVHWYQQKPGQAPVLVVYDDSDRPSGIPERFSGSNS---G
GL-CDR3rev4     SYVLTQPPSVSVAPGQTARITCGGNNIGSKSVHWYQQKPGQAPVLVVYDDSDRPSGIPERFSGSNS---G
GL-CDR3rev5     SYVLTQPPSVSVAPGQTARITCGGNNIGSKSVHWYQQKPGQAPVLVVYDDSDRPSGIPERFSGSNS---G
GL+3            SYVLTQPPSVSVAPGKTARITCGGNNIGSKSVHWYQQKPGQAPVLVIYDDSDRPSGIPERFSGSNS---G
GL-CDR3mat      SYVLTQPPSVSVAPGQTARITCGGNNIGSKSVHWYQQKPGQAPVLVVYDDSDRPSGIPERFSGSNS---G
GL+9            SYVLTQPPSVSVAPGETARISCGGNNIGSKSVQWYQQKPGQAPVLVVYNNQDRPSGIPERFSGSPD---G
PGT121          -------SDISVAPGETARISCGEKSLGSRAVQWYQHRAGQAPSLIIYNNQDRPSGIPERFSGSPDSPFG

                                                   J
V3-21/J3        NTATLTISRVEAGDEADYYCQVWDSSSDHPWVFGGGTKLTVL
GL-CDR3rev1     NTATLTISRVEAGDEADYYCQVWDSSSDHPWVFGGGTKLTVL
GL-CDR3rev2     NTATLTISRVEAGDEADYYCQVWDSRGPTNWVFGGGTKLTVL
GL-CDR3rev3     NTATLTISRVEAGDEADYYCQVWDSSSDHPWVFGGGTKLTVL
GL-CDR3rev4     NTATLTISRVEAGDEADYYCQVWDSSSDHPWVFGGGTKLTVL
GL-CDR3rev5     NTATLTISRVEAGDEADYYCQVWDSRGPTNWVFGGGTKLTVL
GL+3            NTATLTISRVEAGDEADYYCQVWDSRSDHPWVFGGGTKLTVL
GL-CDR3mat      NTATLTISRVEAGDEADYYCQVWDSRGPTNWVFGGGTKLTVL
GL+9            NTATLTISRVEAGDEADYYCQVWDSRSDHPWVFGGGTKLTVL
PGT121          TTATLTITSVEAGDEADYYCHIWDSRVPTKWVFGGGTTLTVL
```

Figure S3

```
BG505_SOSIP.664           AENLWVTVYYGVPVWKDAETTLFCASDAKAYETEKHNVWATHACVPTDPNPQEIHLENVTEEFNMWKNNMVEQMHTDIISLWDQSLKPCVK
BG505_SOSIP.664_2MUT      AENLWVTVYYGVPVWKDAETTLFCASDAKAYETEKHNVWATHACVPTDPNPQEIHLENVTEEFNMWKNNMVEQMHTDIISLWDQSLKPCVK
BG505_SOSIP.664_3MUT      AENLWVTVYYGVPVWKDAETTLFCASDAKAYETEKHNVWATHACVPTDPNPQEIHLENVTEEFNMWKNNMVEQMHTDIISLWDQSLKPCVK
BG505_SOSIP.664_5MUT      AENLWVTVYYGVPVWKDAETTLFCASDAKAYETEKHNVWATHACVPTDPNPQEIHLENVTEEFNMWKNNMVEQMHTDIISLWDQSLKPCVK
BG505_SOSIP.664_6MUT      AENLWVTVYYGVPVWKDAETTLFCASDAKAYETEKHNVWATHACVPTDPNPQEIHLENVTEEFNMWKNNMVEQMHTDIISLWDQSLKPCVK
BG505_SOSIP.664_7MUT      AENLWVTVYYGVPVWKDAETTLFCASDAKAYETEKHNVWATHACVPTDPNPQEIHLENVTEEFNMWKNNMVEQMHTDIISLWDQSLKPCVK
BG505_SOSIP.664_9MUTA     AENLWVTVYYGVPVWKDAETTLFCASDAKAYETEKHNVWATHACVPTDPNPQEIHLENVTEEFNMWKNNMVEQMHTDIISLWDQSLKPCVK
BG505_gp120_9MUTB         -------------VWKDAETTLFCASDAKAYETEKHNVWATHACVPTDPNPQEIHLENVTEEFNMWKNNMVEQMHTDIISAWDQSLKPCVK
BG505_gp120_10MUT_KO      -------------VWKDAETTLFCASDAKAYETEKHNVWATHACVPTDPNPQEIHLENVTEEFNMWKNNMVEQMHTDIISAWDQSLKPCVK
BG505_gp120_10MUT         -------------VWKDAETTLFCASDAKAYETEKHNVWATHACVPTDPNPQEIHLENVTEEFNMWKNNMVEQMHTDIISAWDQSLKPCVK
BG505_SOSIP.664_10MUT     AENLWVTVYYGVPVWKDAETTLFCASDAKAYETEKHNVWATHACVPTDPNPQEIHLENVTEEFNMWKNNMVEQMHTDIISLWDQSLKPCVK
BG505_SOSIP_MD39_10MUTA   AENLWVTVYYGVPVWKDAETTLFCASDAKAYETEKHNVWATHACVPTDPNPQEIHLENVTEEFNMWKNNMVEQMHEDIISLWDQSLKPCVK
BG505_SOSIP.664_11MUTA    AENLWVTVYYGVPVWKDAETTLFCASDAKAYETEKHNVWATHACVPTDPNPQEIHLENVTEEFNMWKNNMVEQMHTDIISLWDQSLKPCVK
BG505_SOSIP_MD39_11MUTB   AENLWVTVYYGVPVWKDAETTLFCASDAKAYETEKHNVWATHACVPTDPNPQEIHLENVTEEFNMWKNNMVEQMHEDIISLWDQSLKPCVK

BG505_SOSIP.664           LTPLCVTLQCTNVTNNITDDMRGELKNCSFNMTTELRDKKQKVYSLFYRLDVVQINENQGNRSNNSNKEYRLINCNTSAITQACPKVSFEP
BG505_SOSIP.664_2MUT      LTPLCVTLQCTNVTNNIIDDMRGELKNCSFNMTTELRDKKQKVYSLFYRLDVVQINENQGNRSNNSNKEYRLINCNTSAITQACPKVSFEP
BG505_SOSIP.664_3MUT      LTPLCVTLQCTNVANNIIDDMRGELKNCSFNMTTELRDKKQKVYSLFYRLDVVQINENQGNRSNNSNKEYRLINCNTSAITQACPKVSFEP
BG505_SOSIP.664_5MUT      LTPLCVTLQCTNYTPNLTNDMRGELKNCSFNMTTELRDKKQKVYSLFYRLDVVQINENQGNRSNNSNKEYRLINCNTSAITQACPKVSFEP
BG505_SOSIP.664_6MUT      LTPLCVTLQCTNYTPNLINDMRGELKNCSFNMTTELRDKKQKVYSLFYRLDVVQINENQGNRSNNSNKEYRLINCNTSAITQACPKVSFEP
BG505_SOSIP.664_7MUT      LTPLCVTLQCTNYAPNLINDMRGELKNCSFNMTTELRDKKQKVYSLFYRLDVVQINENQGNRSNNSNKEYRLINCNTSAITQACPKVSFEP
BG505_SOSIP.664_9MUTA     LTPLCVTLQCTNYAPFLINDMRGELKNCSFNMTTELRDKKQKVYSLFYRLDVVQINENQGNRSNNSNKEYRLINCNTSAITQACPKVSFEP
BG505_gp120_9MUTB         LTPLCVTLQCTNYRPNLINDMRGELKNCSFNMTTELRDKKQKVYSLFYRLDVVQINENQGNRSNNSNKEYRLINCNTSAITQACPKVSFEP
BG505_gp120_10MUT_KO      LTPLCVTLQCTNYAPFLINDMRGELKNCSFNMTTELRDKKQKVYSLFYRLDVVQINENQGNRSNNSNKEYRLINCNTSAITQACPKVSFEP
BG505_gp120_10MUT         LTPLCVTLQCTNYAPFLINDMRGELKNCSFNMTTELRDKKQKVYSLFYRLDVVQINENQGNRSNNSNKEYRLINCNTSAITQACPKVSFEP
BG505_SOSIP.664_10MUT     LTPLCVTLQCTNYAPFLINDMRGELKNCSFNMTTELRDKKQKVYSLFYRLDVVQINENQGNRSNNSNKEYRLINCNTSAITQACPKVSFEP
BG505_SOSIP_MD39_10MUTA   LTPLCVTLQCTNYAPFLINNMRGELKNCSFNMTTELRDKKQKVYSLFYRLDVVQINENQGNRSNNSNKEYRLINCNTSAITQACPKVSFEP
BG505_SOSIP.664_11MUTA    LTPLCVTLQCTNYAPFLINNMRGELKNCSFNMTTELRDKKQKVYSLFYRLDVVQINENQGNRSNNSNKEYRLINCNTSAITQACPKVSFEP
BG505_SOSIP_MD39_11MUTB   LTPLCVTLQCTNYAPNLLSNMRGELKNCSFNMTTELRDKKQKVYSLFYRLDVVQINENQGNRSNNSNKEYRLINCNTSAITQACPKVSFEP

BG505_SOSIP.664           IPIHYCAPAGFAILKCKDKKFNGTGPCPSVSTVQCTHGIKPVVSTQLLLNGSLAEEEVMIRSENITNNAKNILVQFNTPVQINCTRPNNNT
BG505_SOSIP.664_2MUT      IPIHYCAPAGFAILKCKDKKFNGTGPCPSVSTVQCTHGIKPVVSTQLLLNGSLAEEEVMIRSENITNNAKNILVQFNTPVQINCTRPNNNT
BG505_SOSIP.664_3MUT      IPIHYCAPAGFAILKCKDKKFNGTGPCPSVSTVQCTHGIKPVVSTQLLLNGSLAEEEVMIRSENITNNAKNILVQFNTPVQINCTRPNNNT
BG505_SOSIP.664_5MUT      IPIHYCAPAGFAILKCKDKKFNGTGPCPSVSTVQCTHGIKPVVSTQLLLNGSLAEEEVMIRSENITNNAKNILVQFNTPVQINCTRPNNNT
BG505_SOSIP.664_6MUT      IPIHYCAPAGFAILKCKDKKFNGTGPCPSVSTVQCTHGIKPVVSTQLLLNGSLAEEEVMIRSENITNNAKNILVQFNTPVQINCTRPNNNT
BG505_SOSIP.664_7MUT      IPIHYCAPAGFAILKCKDKKFNGTGPCPSVSTVQCTHGIKPVVSTQLLLNGSLAEEEVMIRSENITNNAKNILVQFNTPVQINCTRPNNNT
BG505_SOSIP.664_9MUTA     IPIHYCAPAGFAILKCKDKKFNGTGPCPSVSTVQCTHGIKPVVSTQLLLNGSLAEEEVMIRSENITNNAKNILVQFNTPVQINCTRPNNNT
BG505_gp120_9MUTB         IPIHYCAPAGFAILKCKDKKFNGTGPCPSVSTVQCTHGIKPVVSTQLLLNGSLAEEEVMIRSENITNNAKNILVQFNTPVQINCTRPNNNT
BG505_gp120_10MUT_KO      IPIHYCAPAGFAILKCKDKKFNGTGPCPSVSTVQCTHGIKPVVSTQLLLNGSLAEEEVMIRSENITNNAKNILVQFNTPVQINCTRPNNNA
BG505_gp120_10MUT         IPIHYCAPAGFAILKCKDKKFNGTGPCPSVSTVQCTHGIKPVVSTQLLLNGSLAEEEVMIRSENITNNAKNILVQFNTPVQINCTRPNNNT
BG505_SOSIP.664_10MUT     IPIHYCAPAGFAILKCKDKKFNGTGPCPSVSTVQCTHGIKPVVSTQLLLNGSLAEEEVMIRSENITNNAKNILVQFNTPVQINCTRPNNNT
BG505_SOSIP_MD39_10MUTA   IPIHYCAPAGFAILKCKDKKFNGTGPCPSVSTVQCTHGIKPVVSTQLLLNGSLAEEEVIIRSENITNNAKNILVQLNTPVQINCTRPNNNT
BG505_SOSIP.664_11MUTA    IPIHYCAPAGFAILKCKDKKFNGTGPCPSVSTVQCTHGIKPVVSTQLLLNGSLAEEEVMIRSENITNNAKNILVQFNTPVQINCTRPNNNT
BG505_SOSIP_MD39_11MUTB   IPIHYCAPAGFAILKCKDKKFNGTGPCPSVSTVQCTHGIKPVVSTQLLLNGSLAEEEVIIRSENITNNAKNILVQLNTPVQINCTRPNNNT

BG505_SOSIP.664           RKSIRIGPGQAFYATGDIIGDIRQAHCNVSKATWNETLGKVVKQLRKHFGNNTIIRFANSSGGDLEVTTHSFNCGGEFFYCNTSGLFNSTW
BG505_SOSIP.664_2MUT      RKSIRIGPGQAFYATGDIIGDIRQAHCNVSKATWNETLGKVVKQLRKHFGNNTIIRFANSSGGDLEVTTHSFNCGGEFFYCNTSGLFNSTW
BG505_SOSIP.664_3MUT      RKSIRIGPGQAFYATGDIIGDIRQAHCNVSKATWNETLGKVVKQLRKHFGNNTIIRFANSSGGDLEVTTHSFNCGGEFFYCNTSGLFNSTW
BG505_SOSIP.664_5MUT      RKSIRIGPGQAFYATGDIIGDIRQAHCNVSKATWNETLGKVVKQLRKHFGNNTIIRFANSSGGDLEVTTHSFNCGGEFFYCNTSGLFNSTW
BG505_SOSIP.664_6MUT      RKSIRIGPGQAFYATGDIIGDIRQAHCNVSKATWNETLGKVVKQLRKHFGNNTIIRFANSSGGDLEVTTHSFNCGGEFFYCNTSGLFNSTW
BG505_SOSIP.664_7MUT      RKSIRIGPGQAFYATGDIIGDIRQAHCNVSKATWNETLGKVVKQLRKHFGNNTIIRFANSSGGDLEVTTHSFNCGGEFFYCNTSGLFNSTW
BG505_SOSIP.664_9MUTA     RKSIRIGPGQAFYATGDIIGDIRMAHCNVSKATWNETLGKVVKQLRKHFGNNTIIRFANSSGGDLEVTTHSFNCGGEFFYCNTSGLFNSTW
BG505_gp120_9MUTB         RKSIRIGPGQAFYATGDIIGDIRMAHCNVSKATWNETLGKVVKQLRKHFGNNTIIRFANSSGGDLEVTTHSFNCGGEFFYCNTSGLFNSTW
BG505_gp120_10MUT_KO      RKSIRIGPGQAFYAFGDIIGDIRMAACTVSKATWNETLGKVVKQLRKHFGNNTIIRFANSSGGDLEVTTHSFNCGGEFFYCNTSGLFNSTW
BG505_gp120_10MUT         RKSIRIGPGQAFYAFGDIIGDIRMAHCNVSKATWNETLGKVVKQLRKHFGNNTIIRFANSSGGDLEVTTHSFNCGGEFFYCNTSGLFNSTW
BG505_SOSIP.664_10MUT     RKSIRIGPGQAFYAFGDIIGDIRMAHCNVSKATWNETLGKVVKQLRKHFGNNTIIRFANSSGGDLEVTTHSFNCGGEFFYCNTSGLFNSTW
BG505_SOSIP_MD39_10MUTA   VKSIRIGPGQAFYYTGDIIGDIRMAHCNVSKATWNETLGKVVKQLRKHFGNNTIIRFAQSSGGDLEVTTHSFNCGGEFFYCNTSGLFNSTW
BG505_SOSIP.664_11MUTA    RKSIRIGPGQAFYAFGDIIGDIRMAHCNVSKATWNETLGKVVKQLRKHFGNNTIIRFANSSGGDLEVTTHSFNCGGEFFYCNTSGLFNSTW
BG505_SOSIP_MD39_11MUTB   VKSIRIGPGQAFYYFGDIIGDIRMAHCNVSKATWNETLGKVVKQLRKHFGNNTIIRFAQSSGGDLEVTTHSFNCGGEFFYCNTSGLFNSTW

BG505_SOSIP.664           ISNTSVQGSNSTGSNDSITLPCRIKQIINMWQRIGQAMYAPPIQGVIRCVSNITGLILTRDGGSTNSTTETFRPGGGDMRDNWRSELYKYK
BG505_SOSIP.664_2MUT      ISNTSVQGSNSTGSNDSITLPCRIKQIINMWQRIGQAMYAPPIQGVIRCVSNITGLILTRDGGSTNSTTETFRPGGGDMRDNWRSELYKYK
BG505_SOSIP.664_3MUT      ISNTSVQGSNSTGSNDSITLPCRIKQIINMWQRIGQAMYAPPIQGVIRCVSNITGLILTRDGGSTNSTTETFRPGGGDMRDNWRSELYKYK
BG505_SOSIP.664_5MUT      ISNTSVQGSNSTGSNDSITLPCRIKQIINMWQRIGQAMYAPPIQGVIRCVSNITGLILTRDGGSTNSTTETFRPGGGDMRDNWRSELYKYK
BG505_SOSIP.664_6MUT      ISNTSVQGSNSTGSNDSITLPCRIKQIINMWQRIGQAMYAPPIQGVIRCVSNITGLILTRDGGSTNSTTETFRPGGGDMRDNWRSELYKYK
BG505_SOSIP.664_7MUT      ISNTSVQGSNSTGSNDSITLPCRIKQIINMWQRIGQAMYAPPIQGVIRCVSNITGLILTRDGGSTNSTTETFRPGGGDMRDNWRSELYKYK
BG505_SOSIP.664_9MUTA     ISNTSVQGSNSTGSNDSITLPCRIKQIINMWQRIGQAMYAPPIQGVIRCVSNITGLILTRDGGSTNSTTETFRPGGGDMRDNWRSELYKYK
BG505_gp120_9MUTB         ISNTSVQGSNSTGSNDSITLPCRIKQIINMWQRIGQAMYAPPIQGVIRCVSNITGLILTRDGGSTNSTTETFRPGGGDMRDNWRSELYKYK
BG505_gp120_10MUT_KO      ISNTSVQGSNSTGSNDSITLPCRIKQIINMWQRIGQAMYAPPIQGVIRCVSNITGLILTRDGGSTNSTTETFRPGGGDMRDNWRSELYKYK
BG505_gp120_10MUT         ISNTSVQGSNSTGSNDSITLPCRIKQIINMWQRIGQAMYAPPIQGVIRCVSNITGLILTRDGGSTNSTTETFRPGGGDMRDNWRSELYKYK
BG505_SOSIP.664_10MUT     ISNTSVQGSNSTGSNDSITLPCRIKQIINMWQRIGQAMYAPPIQGVIRCVSNITGLILTRDGGSTNSTTETFRPGGGDMRDNWRSELYKYK
BG505_SOSIP_MD39_10MUTA   ISNTSVQGSNSTGSNDSITLPCRIKQIINMWQRIGQAMYAPPIQGVIRCVSNITGLILTRDGGSTNSTTETFRPGGGDMRDNWRSELYKYK
BG505_SOSIP.664_11MUTA    ISNTSVQGSNSTGSNDSITLPCRIKQIINMWQRIGQAMYAPPIQGVIRCVSNITGLILTRDGGSTNSTTETFRPGGGDMRDNWRSELYKYK
BG505_SOSIP_MD39_11MUTB   ISNTSVQGSNSTGSNDSIVLPCRIKQIINMWQRIGQAMYAPPIQGVIRCVSNITGLILTRDGGSTNSTTETFRPGGGDMRDNWRSELYKYK

BG505_SOSIP.664           VVKIEPLGVAPTRCKRRVVGRRRRRRAVGIGAVFLGFLGAAGSTMGAASMTLTVQARNLLSGIVQQQSNLLRAPEAQQHLLKLTVWGIKQL
BG505_SOSIP.664_2MUT      VVKIEPLGVAPTRCKRRVVGRRRRRRAVGIGAVFLGFLGAAGSTMGAASMTLTVQARNLLSGIVQQQSNLLRAPEAQQHLLKLTVWGIKQL
BG505_SOSIP.664_3MUT      VVKIEPLGVAPTRCKRRVVGRRRRRRAVGIGAVFLGFLGAAGSTMGAASMTLTVQARNLLSGIVQQQSNLLRAPEAQQHLLKLTVWGIKQL
BG505_SOSIP.664_5MUT      VVKIEPLGVAPTRCKRRVVGRRRRRRAVGIGAVFLGFLGAAGSTMGAASMTLTVQARNLLSGIVQQQSNLLRAPEAQQHLLKLTVWGIKQL
BG505_SOSIP.664_6MUT      VVKIEPLGVAPTRCKRRVVGRRRRRRAVGIGAVFLGFLGAAGSTMGAASMTLTVQARNLLSGIVQQQSNLLRAPEAQQHLLKLTVWGIKQL
BG505_SOSIP.664_7MUT      VVKIEPLGVAPTRCKRRVVGRRRRRRAVGIGAVFLGFLGAAGSTMGAASMTLTVQARNLLSGIVQQQSNLLRAPEAQQHLLKLTVWGIKQL
BG505_SOSIP.664_9MUTA     VVKIEPLGVAPTRCKRRVVGRRRRRRAVGIGAVFLGFLGAAGSTMGAASMTLTVQARNLLSGIVQQQSNLLRAPEAQQHLLKLTVWGIKQL
BG505_gp120_9MUTB         VVKIEP-------------------------------------------------------------------------------------
BG505_gp120_10MUT_KO      VVKIEP-------------------------------------------------------------------------------------
BG505_gp120_10MUT         VVKIEP-------------------------------------------------------------------------------------
BG505_SOSIP.664_10MUT     VVKIEPLGVAPTRCKRRVVGRRRRRRAVGIGAVFLGFLGAAGSTMGAASMTLTVQARNLLSGIVQQQSNLLRAPEAQQHLLKLTVWGIKQL
BG505_SOSIP_MD39_10MUTA   VVKIEPLGVAPTRCKRRVVGRRRRRRAVGIGAVSLGFLGAAGSTMGAASMTLTVQARNLLSGIVQQQSNLLRAPEPQQHLLKDTHWGIKQL
BG505_SOSIP.664_11MUTA    VVKIEPLGVAPTRCKRRVVGRRRRRRAVGIGAVFLGFLGAAGSTMGAASMTLTVQARNLLSGIVQQQSNLLRAPEAQQHLLKLTVWGIKQL
BG505_SOSIP_MD39_11MUTB   VVKIEPLGVAPTRCKRRVVGRRRRRRAVGIGAVSLGFLGAAGSTMGAASMTLTVQARNLLSGIVQQQSNLLRAPEPQQHLLKDTHWGIKQL

BG505_SOSIP.664           QARVLAVERYLRDQQLLGIWGCSGKLICCTNVPWNSSWSNRNLSEIWDNMTWLQWDKEISNYTQIIYGLLEESQNQQEKNEQDLLALD
BG505_SOSIP.664_2MUT      QARVLAVERYLRDQQLLGIWGCSGKLICCTNVPWNSSWSNRNLSEIWDNMTWLQWDKEISNYTQIIYGLLEESQNQQEKNEQDLLALD
BG505_SOSIP.664_3MUT      QARVLAVERYLRDQQLLGIWGCSGKLICCTNVPWNSSWSNRNLSEIWDNMTWLQWDKEISNYTQIIYGLLEESQNQQEKNEQDLLALD
BG505_SOSIP.664_5MUT      QARVLAVERYLRDQQLLGIWGCSGKLICCTNVPWNSSWSNRNLSEIWDNMTWLQWDKEISNYTQIIYGLLEESQNQQEKNEQDLLALD
BG505_SOSIP.664_6MUT      QARVLAVERYLRDQQLLGIWGCSGKLICCTNVPWNSSWSNRNLSEIWDNMTWLQWDKEISNYTQIIYGLLEESQNQQEKNEQDLLALD
BG505_SOSIP.664_7MUT      QARVLAVERYLRDQQLLGIWGCSGKLICCTNVPWNSSWSNRNLSEIWDNMTWLQWDKEISNYTQIIYGLLEESQNQQEKNEQDLLALD
BG505_SOSIP.664_9MUTA     QARVLAVERYLRDQQLLGIWGCSGKLICCTNVPWNSSWSNRNLSEIWDNMTWLQWDKEISNYTQIIYGLLEESQNQQEKNEQDLLALD
BG505_gp120_9MUTB         ----------------------------------------------------------------------------------------
BG505_gp120_10MUT_KO      ----------------------------------------------------------------------------------------
BG505_gp120_10MUT         ----------------------------------------------------------------------------------------
BG505_SOSIP.664_10MUT     QARVLAVERYLRDQQLLGIWGCSGKLICCTNVPWNSSWSNRNLSEIWDNMTWLQWDKEISNYTQIIYGLLEESQNQQEKNEQDLLALD
BG505_SOSIP_MD39_10MUTA   QARVLAVEHYLRDQQLLGIWGCSGKLICCTNVPWNSSWSNRNLSEIWDNMTWLQWDKEISNYTQIIYGLLEESQNQQEKNEQDLLALD
BG505_SOSIP.664_11MUTA    QARVLAVERYLRDQQLLGIWGCSGKLICCTNVPWNSSWSNRNLSEIWDNMTWLQWDKEISNYTQIIYGLLEESQNQQEKNEQDLLALD
BG505_SOSIP_MD39_11MUTB   QARVLAVEHYLRDQQLLGIWGCSGKLICCTNVPWNSSWSNRNLSEIWDNMTWLQWDKEISNYTQIIYGLLEESQNQQEKNEQDLLALD
```

Figure S4

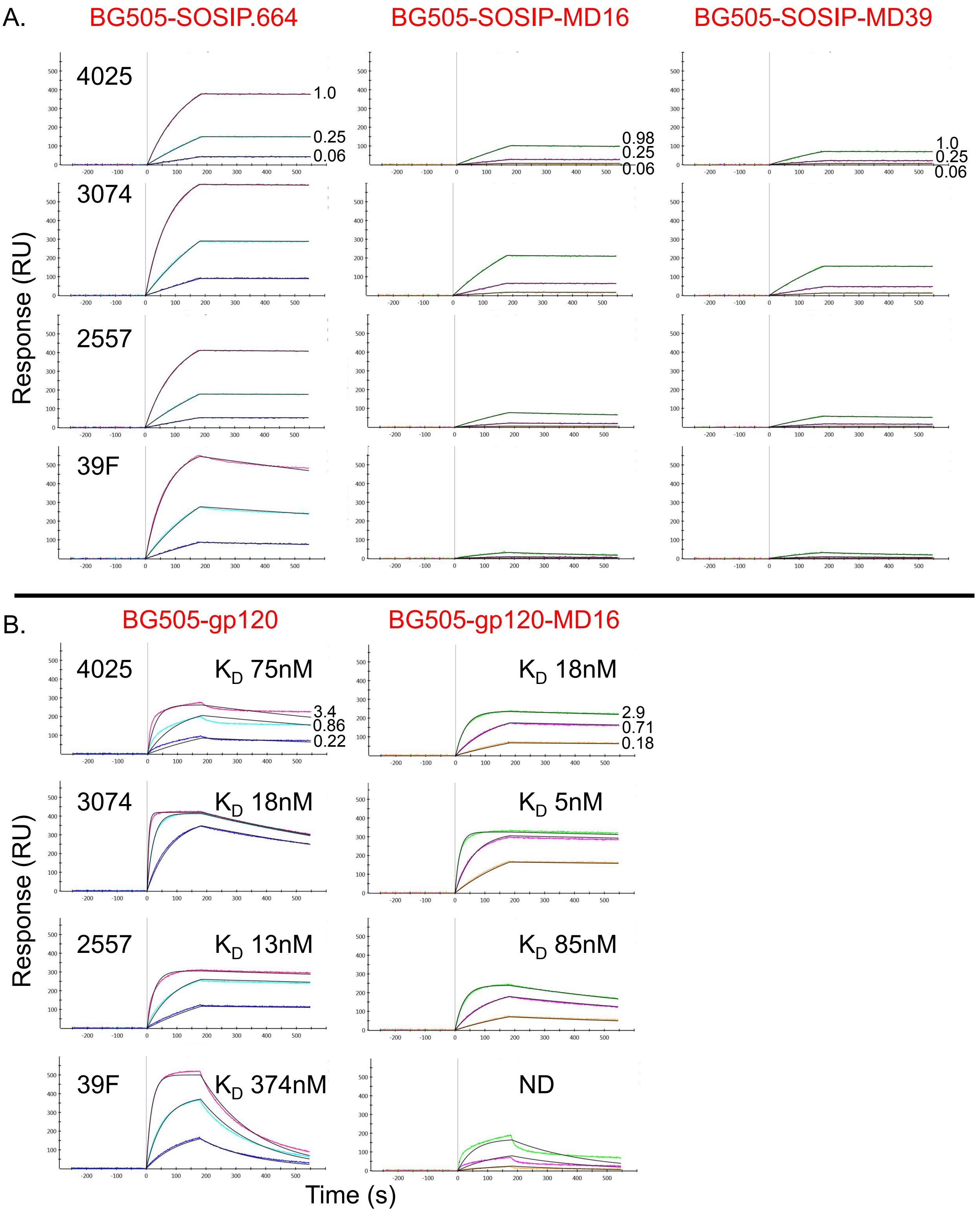
A.
BG505-SOSIP.664
BG505-SOSIP-MD16
BG505-SOSIP-MD39
4025
3074
2557
39F
1.0
0.25
0.06
0.98
0.25
0.06
1.0
0.25
0.06
Response (RU)
B.
BG505-gp120
BG505-gp120-MD16
K_D 75nM
K_D 18nM
3.4
0.86
0.22
2.9
0.71
0.18
K_D 18nM
K_D 5nM
K_D 13nM
K_D 85nM
K_D 374nM
ND
Response (RU)
Time (s)

Figure S5

A.

```
BG505_SOSIP.664     AENLWVTVYYGVPVWKDAETTLFCASDAKAYETEKHNVWATHACVPTDPNPQEIHLENVTEEFNMWKNNMVEQMHTDIIS
BG505_SOSIP_rare3   AENLWVTVYYGVPVWKDAETTLFCASDAKAYETEKHNVWATHACVPTDPNPQEIHLENVTEEFNMWKNNMVEQMHEDIIS
BG505_SOSIP_MD16    AENLWVTVYYGVPVWKDAETTLFCASDAKAYETEKHNVWATHACVPTDPNPQEIHLENVTEEFNMWKNNMVEQMHTDIIS
BG505_SOSIP_MD2     AENLWVTVYYGVPVWKDAETTLFCASDAKAYETEKHNVWATHACVPTDPNPQEIHLENVTEEFNMWKNNMVEQMHTDIIS
BG505_SOSIP_MD33    AENLWVTVYYGVPVWKDAETTLFCASDAKAYETEKHNVWATHACVPTDPNPQEIHLENVTEEFNMWKNNMVEQMHTDIIS
BG505_SOSIP_MD39    AENLWVTVYYGVPVWKDAETTLFCASDAKAYETEKHNVWATHACVPTDPNPQEIHLENVTEEFNMWKNNMVEQMHEDIIS

BG505_SOSIP.664     LWDQSLKPCVKLTPLCVTLQCTNVTNNITDDMRGELKNCSFNMTTELRDKKQKVYSLFYRLDVVQINENQGNRSNNSNKE
BG505_SOSIP_rare3   LWDQSLKPCVKLTPLCVTLQCTNVTNNITDDMRGELKNCSFNMTTELRDKKQKVYSLFYRLDVVQINENQGNRSNNSNKE
BG505_SOSIP_MD16    LWDQSLKPCVKLTPLCVTLQCTNVTNNITDDMRGELKNCSFNMTTELRDKKQKVYSLFYRLDVVQINENQGNRSNNSNKE
BG505_SOSIP_MD2     LWDQSLKPCVKLTPLCVTLQCTNVTNNITDDMRGELKNCSFNMTTELRDKKQKVYSLFYRLDVVQINENQGNRSNNSNKE
BG505_SOSIP_MD33    LWDQSLKPCVKLTPLCVTLQCTNVTNNITDDMRGELKNCSFNMTTELRDKKQKVYSLFYRLDVVQINENQGNRSNNSNKE
BG505_SOSIP_MD39    LWDQSLKPCVKLTPLCVTLQCTNVTNNITDDMRGELKNCSFNMTTELRDKKQKVYSLFYRLDVVQINENQGNRSNNSNKE

BG505_SOSIP.664     YRLINCNTSAITQACPKVSFEPIPIHYCAPAGFAILKCKDKKFNGTGPCPSVSTVQCTHGIKPVVSTQLLLNGSLAEEEV
BG505_SOSIP_rare3   YRLINCNTSAITQACPKVSFEPIPIHYCAPAGFAILKCKDKKFNGTGPCPSVSTVQCTHGIKPVVSTQLLLNGSLAEEEV
BG505_SOSIP_MD16    YRLINCNTSAITQACPKVSFEPIPIHYCAPAGWAILKCKDKKFNGTGPCPSVSTVQCTHGIKPVVSTQLLLNGSLAEEEV
BG505_SOSIP_MD2     YRLINCNTSAITQACPKVSFEPIPIHYCAPAGFAILKCKDKKFNGTGPCPSVSTVQCTHGIKPVVSTQLLLNGSLAEEEV
BG505_SOSIP_MD33    YRLINCNTSAITQACPKVSFEPIPIHYCAPAGFAILKCKDKKFNGTGPCPSVSTVQCTHGIKPVVSTQLLLNGSLAEEEV
BG505_SOSIP_MD39    YRLINCNTSAITQACPKVSFEPIPIHYCAPAGFAILKCKDKKFNGTGPCPSVSTVQCTHGIKPVVSTQLLLNGSLAEEEV

BG505_SOSIP.664     MIRSENITNNAKNILVQFNTPVQINCTRPNNNTRKSIRIGPGQAFYATGDIIGDIRQAHCNVSKATWNETLGKVVKQLRK
BG505_SOSIP_rare3   IIRSENITNNAKNILVQLNAPVQINCTRPNNNTRKSIRIGPGQAFYATGDIIGDIRQAHCNVSKATWNETLGKVVKQLRK
BG505_SOSIP_MD16    MIRSENITNNAKNILVQFNTPVQINCTRPNNNTVKSIRIGPGQAFYYTGDIIGDIRQAHCNVSKATWNETLGKVVKQLRK
BG505_SOSIP_MD2     MIRSENITNNAKNILVQFNTPVQINCTRPNNNTRKSIRIGPGQAFYATGDIIGDIRQAHCNVSKATWNETLGKVVKQLRK
BG505_SOSIP_MD33    MIRSENITNNAKNILVQFNTPVQINCTRPNNNTRKSIRIGPGQAFYATGDIIGDIRQAHCNVSKATWNETLGKVVKQLRK
BG505_SOSIP_MD39    IIRSENITNNAKNILVQLNTPVQINCTRPNNNTVKSIRIGPGQAFYYTGDIIGDIRQAHCNVSKATWNETLGKVVKQLRK

BG505_SOSIP.664     HFGNNTIIRFANSSGGDLEVTTHSFNCGGEFFYCNTSGLFNSTWISNTSVQGSNSTGSNDSITLPCRIKQIINMWQRIGQ
BG505_SOSIP_rare3   HFGNNTIIRFAQSSGGDLEVTTHSFNCGGEFFYCNTSGLFNSTWISNTSVQGSNSTGSNDSITLPCRIKQIINMWQRIGQ
BG505_SOSIP_MD16    HFGNNTIIRFANSSGGDLEVTTHSFNCGGEFFYCNTSGLFNSTWISNTSVQGSNSTGSNDSITLPCRIKQIINMWQRIGQ
BG505_SOSIP_MD2     HFGNNTIIRFANSSGGDLEVTTHSFNCGGEFFYCNTSGLFNSTWISNTSVQGSNSTGSNDSITLPCRIKQIINMWQRIGQ
BG505_SOSIP_MD33    HFGNNTIIRFANSSGGDLEVTTHSFNCGGEFFYCNTSGLFNSTWISNTSVQGSNSTGSNDSITLPCRIKQIINMWQRIGQ
BG505_SOSIP_MD39    HFGNNTIIRFAQSSGGDLEVTTHSFNCGGEFFYCNTSGLFNSTWISNTSVQGSNSTGSNDSITLPCRIKQIINMWQRIGQ

BG505_SOSIP.664     AMYAPPIQGVIRCVSNITGLILTRDGGSTNSTTETFRPGGGDMRDNWRSELYKYKVVKIEPLGVAPTRCKRRVVGRRRRR
BG505_SOSIP_rare3   AMYAPPIQGVIRCVSNITGLILTRDGGSTNSTTETFRPGGGDMRDNWRSELYKYKVVKIEPLGVAPTRCKRRVVGRRRRR
BG505_SOSIP_MD16    AMYAPPIQGVIRCVSNITGLILTRDGGSTNSTTETFRPGGGDMRDNWRSELYKYKVVKIEPLGVAPTRCKRRVVGRRRRR
BG505_SOSIP_MD2     AMYAPPIQGVIRCVSNITGLILTRDGGSTNSTTETFRPGGGDMRDNWRSELYKYKVVKIEPLGVAPTRCKRRVVGRRRRR
BG505_SOSIP_MD33    AMYAPPIQGVIRCVSNITGLILTRDGGSTNSTTETFRPGGGDMRDNWRSELYKYKVVKIEPLGVAPTRCKRRVVGRRRRR
BG505_SOSIP_MD39    AMYAPPIQGVIRCVSNITGLILTRDGGSTNSTTETFRPGGGDMRDNWRSELYKYKVVKIEPLGVAPTRCKRRVVGRRRRR

BG505_SOSIP.664     RAVGIGAVFLGFLGAAGSTMGAASMTLTVQARNLLSGIVQQQSNLLRAPEAQQHLLKLTVWGIKQLQARVLAVERYLRDQ
BG505_SOSIP_rare3   RAVGIGAVFLGFLGAAGSTMGAASMTLTVQARNLLSGIVQQQSNLLRAPEAQQHLLKLTVWGIKQLQARVLAVERYLRDQ
BG505_SOSIP_MD16    RAVGIGAVFLGFLGAAGSTMGAASMTLTVQARNLLSGIVQQQSNLLRAPEAQQHLLKDTVWGIKQLQARVLAVERYLRDQ
BG505_SOSIP_MD2     RAVGIGAVFLGFLGAAGSTMGAASMTLTVQARNLLSGIVQQQSNLLRAPEAQQHLLKDTVWGIKQLQARVLAVERYLRDQ
BG505_SOSIP_MD33    RAVGIGAVSLGFLGAAGSTMGAASMTLTVQARNLLSGIVQQQSNLLRAPEPQQHLLKDTHWGIKQLQARVLAVEHYLRDQ
BG505_SOSIP_MD39    RAVGIGAVSLGFLGAAGSTMGAASMTLTVQARNLLSGIVQQQSNLLRAPEPQQHLLKDTHWGIKQLQARVLAVEHYLRDQ

BG505_SOSIP.664     QLLGIWGCSGKLICCTNVPWNSSWSNRNLSEIWDNMTWLQWDKEISNYTQIIYGLLEESQNQQEKNEQDLLALD
BG505_SOSIP_rare3   QLLGIWGCSGKLICCTNVPWNSSWSNRNLSEIWDNMTWLQWDKEISNYTQIIYGLLEESQNQQEKNEQDLLALD
BG505_SOSIP_MD16    QLLGIWGCSGKLICCTNVPWNSSWSNRNLSEIWDNMTWLQWDKEISNYTQIIYGLLEESQNQQEKNEQDLLALD
BG505_SOSIP_MD2     QLLGIWGCSGKLICCTNVPWNSSWSNRNLSEIWDNMTWLQWDKEISNYTQIIYGLLEESQNQQEKNEQDLLALD
BG505_SOSIP_MD33    QLLGIWGCSGKLICCTNVPWNSSWSNRNLSEIWDNMTWLQWDKEISNYTQIIYGLLEESQNQQEKNEQDLLALD
BG505_SOSIP_MD39    QLLGIWGCSGKLICCTNVPWNSSWSNRNLSEIWDNMTWLQWDKEISNYTQIIYGLLEESQNQQEKNEQDLLALD
```

B.

Trimer	Tm (°C)
BG505 SOSIP	66.65
rare3	68.08
MD16	66.5
MD2	66.99
MD33	70.65
MD39	77.17

C.

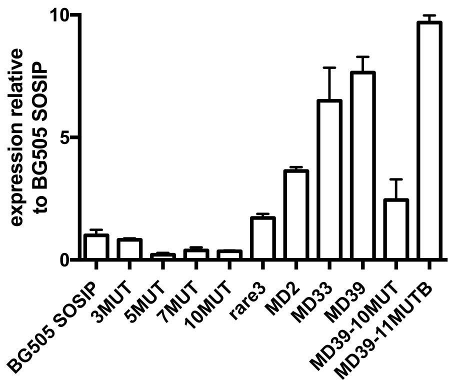

Figure S6

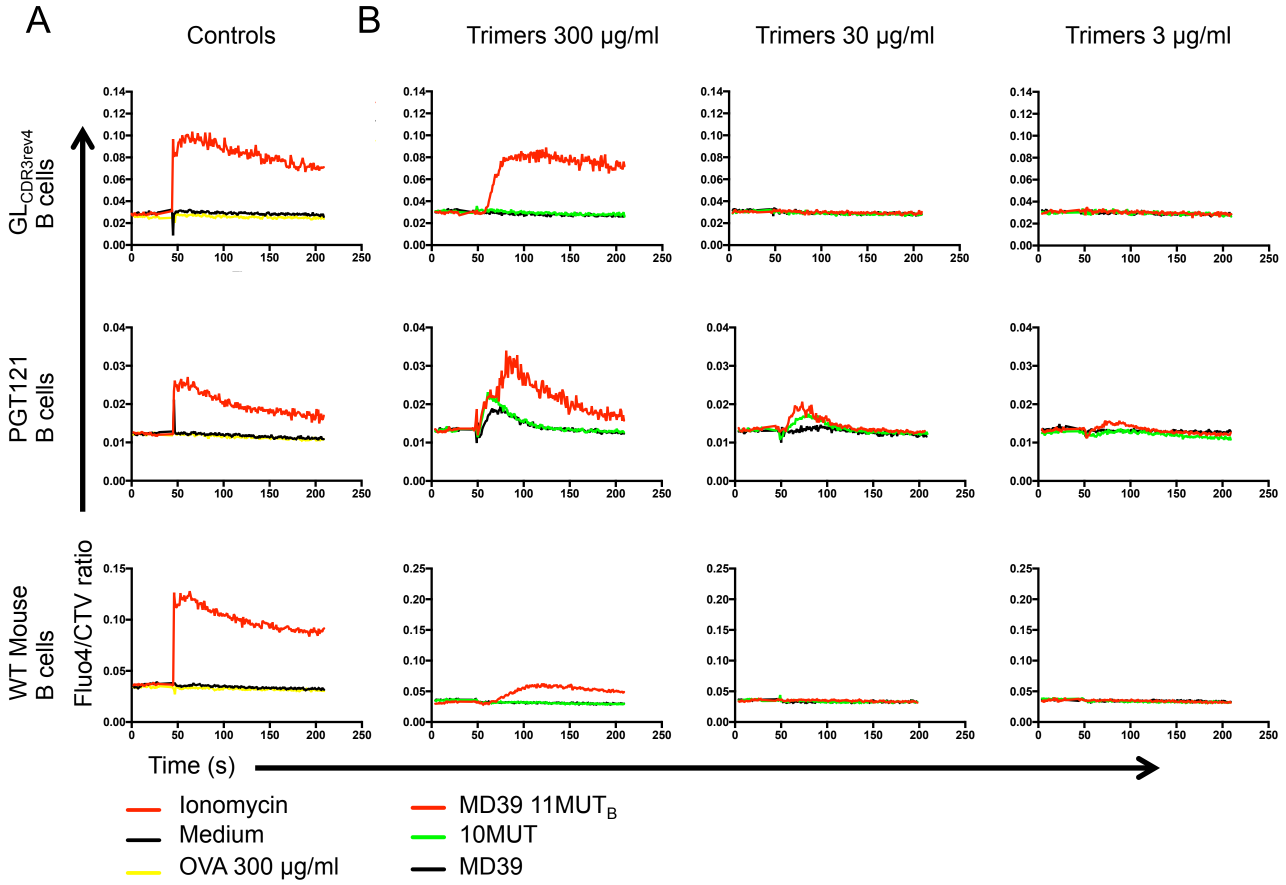
A
Controls
B
Trimers 300 µg/ml
Trimers 30 µg/ml
Trimers 3 µg/ml
GLCDR3rev4 B cells
PGT121 B cells
WT Mouse B cells
Fluo4/CTV ratio
Time (s)
Ionomycin
Medium
OVA 300 µg/ml
MD39 11MUTB
10MUT
MD39

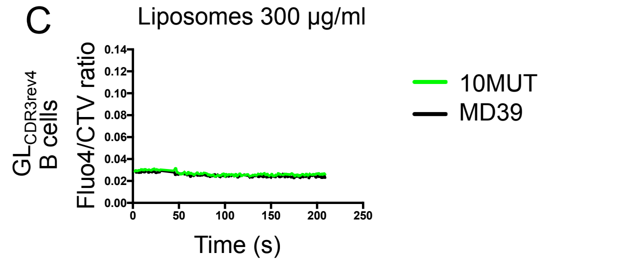
C
Liposomes 300 µg/ml
GLCDR3rev4 B cells
Fluo4/CTV ratio
Time (s)
10MUT
MD39

Figure S7

A

	Dissociation constant K_d (nM)							
	VRC01	121	128	151	145	1400	Tm (°C)	Yield PEI
**BG505**	131	22	8	10	6	2	66	1
**MD39-VLC1**	416	42	5	10	31	-	73	13.7
**MD39-VLC2**	124	21	12	11	20	12	77	6.2
**MD39-VLC3**	449	15	7	11	33	-	77	7.0
**MD39-VLC4**	167	13	2	11	27	-	76	6.3
**MD39-VLC5**	121	50	7	10	11	-	74	10.5

B

```
BG505_SOSIP_D664_MD39     AENLWVTVYYGVPVWKDAETTLFCASDAKAYETEKHNVWATHACVPTDPNPQEIHLENVTEEFNMWKNNMVE
BG505_SOSIP_MD39_VLC1-03 AENLWVTVYYGVPVWKDAETTLFCASDAKAYETEKHNVWATHACVPTDPNPQEIHLENVTEEFNMWKNNMVE
BG505_SOSIP_MD39_VLC2-04 AENLWVTVYYGVPVWKDAETTLFCASDAKAYETEKHNVWATHACVPTDPNPQEIHLENVTEEFNMWKNNMVE
BG505_SOSIP_MD39_VLC2-08 AENLWVTVYYGVPVWKDAETTLFCASDAKAYETEKHNVWATHACVPTDPNPQEIHLENVTEEFNMWKNNMVE
BG505_SOSIP_MD39_VLC3-13 AENLWVTVYYGVPVWKDAETTLFCASDAKAYETEKHNVWATHACVPTDPNPQEIHLENVTEEFNMWKNNMVE

BG505_SOSIP_D664_MD39     QMHEDIISLWDQSLKPCVKLTPLCVTLQC------TNVTNNITDD----MRGELKNCSFNMTTELRDKKQKV
BG505_SOSIP_MD39_VLC1-03 QMHEDIISLWDQSLKPCVKLTPLCVTLQC------TDWDNATLAN----MTGEIKNCSFNMTTELRDKKQKV
BG505_SOSIP_MD39_VLC2-04 QMHEDIISLWDQSLKPCVKLTPLCVTLQCSDYEGNTTRQNITMKE----EKGEIKNCSFNMTTELRDKKQKV
BG505_SOSIP_MD39_VLC2-08 QMHEDIISLWDQSLKPCVKLTPLCVTLQCVTLKNCSNSNCSISRNISIEMDGEIKNCSFNMTTELRDKKQKV
BG505_SOSIP_MD39_VLC3-13 QMHEDIISLWDQSLKPCVKLTPLCVTLQCTNAAL-TNVTITNGPN----ITEEIRNCSFNMTTELRDKKQKV

BG505_SOSIP_D664_MD39     YSLFYRLDVVQINEN-----------QGNRSNNSNKEYRLINCNTSAITQACPKVSFEPIPIHYCAPAGFAI
BG505_SOSIP_MD39_VLC1-03 YSLFYELDIIPIENE-----------YISNNNTSNTSYRLINCNTSAITQACPKVSFEPIPIHYCAPAGFAI
BG505_SOSIP_MD39_VLC2-04 YSLFYKLDITPIEEDNNS--NNSSSANSSNSNANYTNYRLINCNTSAITQACPKVSFEPIPIHYCAPAGFAI
BG505_SOSIP_MD39_VLC2-08 YSLFYRLDIVPIESSNNSQLSNNSQVSNNSQSSNYSQYRLINCNTSAITQACPKVSFEPIPIHYCAPAGFAI
BG505_SOSIP_MD39_VLC3-13 YSLFYKLDLVQINGS-------------------GGEYRLINCNTSAITQACPKVSFEPIPIHYCAPAGFAI

BG505_SOSIP_D664_MD39     LKCKDKKFNGTGPCPSVSTVQCTHGIKPVVSTQLLLNGSLAEEEVIIRSENITNNAKNILVQLNTPVQINCT
BG505_SOSIP_MD39_VLC1-03 LKCKDKKFNGTGPCPSVSTVQCTHGIKPVVSTQLLLNGSLAEEEVIIRSENITNNAKNILVQLNTPVQINCT
BG505_SOSIP_MD39_VLC2-04 LKCKDKKFNGTGPCPSVSTVQCTHGIKPVVSTQLLLNGSLAEEEVIIRSENITNNAKNILVQLNTPVQINCT
BG505_SOSIP_MD39_VLC2-08 LKCKDKKFNGTGPCPSVSTVQCTHGIKPVVSTQLLLNGSLAEEEVIIRSENITNNAKNILVQLNTPVQINCT
BG505_SOSIP_MD39_VLC3-13 LKCKDKKFNGTGPCPSVSTVQCTHGIKPVVSTQLLLNGSLAEEEVIIRSENITNNAKNILVQLNTPVQINCT

BG505_SOSIP_D664_MD39     RPNNNTVKSIRIGPGQAFYYTGDIIGDIRQAHCNVSKATWNETLGKVVKQLRKHFGNNTIIRFAQSSGGDLE
BG505_SOSIP_MD39_VLC1-03 RPNNNTVKSIRIGPGQAFYYTGDIIGDIRQAHCNVSATQWEQTLKGIAAKLLEHFGNNTIIRFAQSSGGDLE
BG505_SOSIP_MD39_VLC2-04 RPNNNTVKSIRIGPGQAFYYTGDIIGDIRQAHCNVSGTKWKNTLKQIVKKLGDHFGNNTIIRFAQSSGGDLE
BG505_SOSIP_MD39_VLC2-08 RPNNNTVKSIRIGPGQAFYYTGDIIGDIRQAHCNVSKKDWEKTLQQVATKLGQHFGNNTIIRFAQSSGGDLE
BG505_SOSIP_MD39_VLC3-13 RPNNNTVKSIRIGPGQAFYYTGDIIGDIRQAHCNVSGTKWNETLKQVAGKLRKHFGNNTIIRFAQSSGGDLE

BG505_SOSIP_D664_MD39     VTTHSFNCGGEFFYCNTSGLFNSTWISNT-----SVQGSNSTGSNDSITLPCRIKQIINMWQRIGQAMYAPP
BG505_SOSIP_MD39_VLC1-03 VTTHSFNCGGEFFYCNTSGLFNGSSWNLN-----KTKENTTNLENGTITLPCRIKQIINMWQRIGQAMYAPP
BG505_SOSIP_MD39_VLC2-04 VTTHSFNCGGEFFYCNTSGLFNSTWNRTNGTWN-DVEGLNYTNGNDTITLPCRIKQIINMWQRIGQAMYAPP
BG505_SOSIP_MD39_VLC2-08 VTTHSFNCGGEFFYCNTSGLFNSTWIRNSSNSTWNSSASNSTELNSNITLPCRIKQIINMWQRIGQAMYAPP
BG505_SOSIP_MD39_VLC3-13 VTTHSFNCGGEFFYCNTSGLFNSTWPENG-----TMEGS-----NGTITLPCRIKQIINMWQRIGQAMYAPP

BG505_SOSIP_D664_MD39     IQGVIRCVSNITGLILTRDGGSTN-STTETFRPGGGDMRDNWRSELYKYKVVKIEPLGVAPTRCKRRVVGRR
BG505_SOSIP_MD39_VLC1-03 IQGVIRCVSNITGLILTRDGGNKS-AGIETFRPGGGDMRDNWRSELYKYKVVKIEPLGVAPTRCKRRVVGRR
BG505_SOSIP_MD39_VLC2-04 IQGVIRCVSNITGLILTRDGGNDT-DKNETFRPGGGDMRDNWRSELYKYKVVKIEPLGVAPTRCKRRVVGRR
BG505_SOSIP_MD39_VLC2-08 IQGVIRCVSNITGLILTRDGGHETENKTETFRPGGGDMRDNWRSELYKYKVVKIEPLGVAPTRCKRRVVGRR
BG505_SOSIP_MD39_VLC3-13 IQGVIRCVSNITGLILTRDGGNST-TDTETFRPGGGDMRDNWRSELYKYKVVKIEPLGVAPTRCKRRVVGRR

BG505_SOSIP_D664_MD39     RRRRAVGIGAVSLGFLGAAGSTMGAASMTLTVQARNLLSGIVQQQSNLLRAPEPQQHLLKDTHWGIKQLQAR
BG505_SOSIP_MD39_VLC1-03 RRRRAVGIGAVSLGFLGAAGSTMGAASMTLTVQARNLLSGIVQQQSNLLRAPEPQQHLLKDTHWGIKQLQAR
BG505_SOSIP_MD39_VLC2-04 RRRRAVGIGAVSLGFLGAAGSTMGAASMTLTVQARNLLSGIVQQQSNLLRAPEPQQHLLKDTHWGIKQLQAR
BG505_SOSIP_MD39_VLC2-08 RRRRAVGIGAVSLGFLGAAGSTMGAASMTLTVQARNLLSGIVQQQSNLLRAPEPQQHLLKDTHWGIKQLQAR
BG505_SOSIP_MD39_VLC3-13 RRRRAVGIGAVSLGFLGAAGSTMGAASMTLTVQARNLLSGIVQQQSNLLRAPEPQQHLLKDTHWGIKQLQAR

BG505_SOSIP_D664_MD39     VLAVEHYLRDQQLLGIWGCSGKLICCTNVPWNSSWSNRNLSEIWDNMTWLQWDKEISNYTQIIYGLLEESQN
BG505_SOSIP_MD39_VLC1-03 VLAVEHYLRDQQLLGIWGCSGKLICCTNVPWNSSWSNRNLSEIWDNMTWLQWDKEISNYTQIIYGLLEESQN
BG505_SOSIP_MD39_VLC2-04 VLAVEHYLRDQQLLGIWGCSGKLICCTNVPWNSSWSNRNLSEIWDNMTWLQWDKEISNYTQIIYGLLEESQN
BG505_SOSIP_MD39_VLC2-08 VLAVEHYLRDQQLLGIWGCSGKLICCTNVPWNSSWSNRNLSEIWDNMTWLQWDKEISNYTQIIYGLLEESQN
BG505_SOSIP_MD39_VLC3-13 VLAVEHYLRDQQLLGIWGCSGKLICCTNVPWNSSWSNRNLSEIWDNMTWLQWDKEISNYTQIIYGLLEESQN

BG505_SOSIP_D664_MD39     QQEKNEQDLLALD
BG505_SOSIP_MD39_VLC1-03 QQEKNEQDLLALD
BG505_SOSIP_MD39_VLC2-04 QQEKNEQDLLALD
BG505_SOSIP_MD39_VLC2-08 QQEKNEQDLLALD
BG505_SOSIP_MD39_VLC3-13 QQEKNEQDLLALD
```

C

Position	N332-epitope Diversity
**156**	N(97%)
**158**	S(89%), T(10%)
**295**	N(66%), V(13%)
**297**	T(81%), I(12%)
**299**	P(98%)
**301**	N(95%)
**303**	T(94%)
**320**	T(90%)
**321**	G(79%)
**322**	D(49%), E(22%)
**323**	I(85%)
**324**	G(99%)
**325**	D(80%), N(16%)
**326**	I(96%)
**327**	R(98%)
**328**	Q(65%), K(24%)
**330**	H(66%), Y(31%)
**332**	N(72%), E(12%)
**334**	S(67%), N(29%)
**386**	N(90%)
**388**	T(48%), S(48%)
**389**	Q(26%), K(25%), G(19%)
**392**	N(92%)
**394**	T(79%)
**415**	T(71%), I(22%)
**417**	P(79%), Q(16%)
**419**	R(81%), K(18%)
**442**	N(27%), Q(22%)
**444**	T(29%), R(29%), N(20%)

Supplemental Figure Legends

Figure S1. Mammalian display overview related to Figure 1.
(A) Schematic of mammalian display procedure.
(B) Example FACS plots for unsorted mammalian display library (top) and the same library sorted 3 times (bottom), see Extended Experimental Procedures.
(C) Sequences of BG505-SOSIP and BG505-gp120 used for mammalian display. Leader peptides are shown in red, the cMyc epitope is shown in green and the PDGFR TM is shown in blue.

Figure S2. Germline-reverted PGT121 Abs, related to Figure 1. Sequences of PGT121 and germline-reverted variants with mutations highlighted relative to germline V4-59/D3-3/J6 (heavy chain) and V3-21/J3 (light chain) genes.

Figure S3. PGT121 germline targeting gp120s and gp140s, related to Figure 1. Designed germline targeting trimer and gp120 sequences are shown with mutations from BG505-SOSIP highlighted. BG505-SOSIP.D664 contains the mutation T332N, not highlighted.

Figure S4. SPR binding data for V3 Abs binding to gp140 SOSIPs and their matching gp120s, related to Figure 2.
(A) Comparison of V3 binding between WT BG505-SOSIP and BG505-SOSIP variants containing R304V and A319Y V3 mutations. For the 4025 SPR plots, the gp140 concentrations tested are shown in μM next to each relevant sensogram and are equivalent for all V3 Abs tested.
(B) Comparison of V3 binding to WT gp120 and gp120-MD16 containing the R304V and A319Y mutations. For the 4025 SPR plots, the gp120 concentrations tested are shown in μM next to each relevant sensogram and are equivalent for all V3 Abs tested.

Figure S5. Trimers with improved thermostability, expression, or antigenic profile, related to Figure 2.
(A) Sequences of designed stabilized native-like trimers with mutations from BG505 SOSIP.D664 highlighted.
(B) Melting temperature of stabilized trimers as assessed by DSC.
(C) Native-like trimers with and without stabilizing mutations were transiently transfected in 293F cells and expression levels were determined by capture ELISA using PGT145 Fab for immobilization and PGT151 IgG for detection. Values are the mean ± SD of 3 replicate transfections.

Figure S6. Ex vivo B cell activation assay, related to Figure 5.
Ca^{2+} flux transients detected as increases in Fluo-4 fluorescence after addition of (A) control activators (Ionomycin and IgM positive controls, Ovalbumin negative control), (B) trimer (MD39, 10MUT, MD39-$11MUT_B$) at the indicated concentrations, or (C) trimer-liposomes for 10MUT or MD39 at 300 μg/mL. In (A) and (B), data are shown for germline-reverted PGT121 ($GL_{CDR3rev4}$) B cells (top), mature PGT121 B cells (middle), and WT mouse B cells (bottom).

Figure S7. Sequences and biophysical properties of the MD39-based VLC native-like trimer cocktail, and N332-epitope sequence diversity, Related to Figure 7.
A) Biophysical characterization of the MD39-based VLC cocktail.
(B) Sequences of MD39-based VLC cocktail members, with changes relative to MD39 highlighted in green.
(C) List of interface positions on the BG505 SOSIP trimer near N332-supersite bnAb epitopes (PGT122, PGT128, PGT135), showing the frequencies of the amino acids found at those positions in 10% or more of 3,897 unique HIV Env sequences isolated from infected individuals obtained from www.hiv.lanl.gov.

Table S1. The binding affinities of germline targeting gp120s, related to Figure 1.

BG505-gp120	PGT121	3H3L	GL+9	GL+3	$GL_{CDR3-mat}$	$GL_{CDR3-rev5}$	$GL_{CDR3-rev4}$	$GL_{CDR3-rev3}$	$GL_{CDR3-rev2}$	$GL_{CDR3-rev1}$	GL_{H-rev4} 121_L	121_H GL_{L-rev4}
WT (T332N)	7.5	250	28000	>128000	>128000	>8000	>84000	>128000	-	>8000	600	>38000
2MUT	2.7	-	4900	>40000	-	-	>40000	-	-	-	63	>40000
3MUT	4.6	19	1600	>28000	>21000	-	>28000	>11000	-	-	22	>28000
5MUT	5.7	2.5	18	WB	WB	-	>34000	-	-	-	6	13000
6MUT	1.4	-	19	>24000	-	-	>24000	-	-	-	5	>24000
7MUT	1.2	0.25	3	12200	44000	-	>36000	-	-	-	1.3	>36000
$9MUT_A$	0.57	-	-	2700	2900	-	>70000	-	-	-	-	57000
$9MUT_B$	1.5	28	-	29000	WB	-	>107000	-	-	-	220	39000
10MUT	0.59	0.04	1.2	1200	790	WB	>150000	WB	>150000	WB	-	47000
10MUT-KO	435	-	-	-	>21000	-	>21000	-	-	-	20000	>21000
$11MUT_A$	-	-	-	-	1200	-	WB	-	-	-	-	51000
$11MUT_B$	0.15	0.075	0.6	600	840	7700	WB	3000	-	5200	-	-

Values are K_Ds (nM) measured by SPR.
WB, weak binding, not quantified.
-, not measured.

Table S2. Data collection and refinement statistics, related to Figure 3.

Beamline	APS 23-ID-D
Wavelength (Å)	1.03322
Resolution (Å)[a]	49.6 – 4.5 (4.58 – 4.5)
Space group	$P6_3$
Unit cell (Å, °)	127.92, 127.92, 313.89 90, 90, 120
Total reflections	73,804 (3970)
Unique reflections	17,067 (887)
Multiplicity	4.3 (4.4)
Completeness (%)	98.4 (98.9)
Mean (I/σ_I)	4.75 (1.1)
R_{merge}[b]	0.213 (1.00)
R_{meas}[c]	0.207 (1.00)
R_{pim}[d]	0.175 (1.00)
$CC_{1/2}$[e]	0.76 (0.63)
R_{work}	0.272 (0.382)
R_{free}	0.309 (0.372)
# reflections used in refinement (work/free)	16142/844
# Protein atoms	11183
# Carbohydrate atoms	653
# Waters	0
# Protein residues	1452
RMS (bonds)	0.012
RMS (angles)	1.55
Ramachandran favored, outliers (%)	96.2, 0.3
Clashscore[f]	7.8
Wilson B (Å^2)	105.6
Average B (Å^2)	186.0

[a]Numbers in parentheses are for highest resolution shell
[b]$R_{merge} = \Sigma_{hkl} \Sigma_{i=1,n}|I_i(hkl) - \langle I(hkl)\rangle| / \Sigma_{hkl} \Sigma_{i=1,n} I_i(hkl)$
[c]$R_{meas} = \Sigma_{hkl} \sqrt{(n/n-1)}\, \Sigma_{i=1,n}|I_i(hkl) - \langle I(hkl)\rangle| / \Sigma_{hkl} \Sigma_{i=1,n} I_i(hkl)$
[d]$R_{pim} = \Sigma_{hkl} \sqrt{(1/n-1)}\, \Sigma_{i=1,n}|I_i(hkl) - \langle I(hkl)\rangle| / \Sigma_{hkl} \Sigma_{i=1,n} I_i(hkl)$
[e]$CC_{1/2}$ = Pearson Correlation Coefficient between two random half datasets
[f]Number of unfavorable all-atom steric overlaps ≥ 0.4Å per 1000 atoms

Table S3. Sequential boosting pairs that were eliminated based on violation of directionality, related to Figure 7.

Sequential boosting pair	Directionality violation
11B ➔ 10/9A	11B contains the native residue N137 which is mutated to F in 10/9A
6 ➔ 3	6 contains the native glycosylation site at N133 which is mutated in 3
5 ➔ 3	5 contains native glycosylation sites at N133 and N137 and both are mutated in 3
5 ➔ 2	5 contains native glycosylation site at N137 which is mutated in 2

Any boosting pair in which the first immunogen contains a native residue that is mutated in the second immunogen is a violation of directionality. The immunogen names have "MUT" removed for simplicity.

Table S4. Characteristics of sequential boosting pairs that obey directionality, related to Figure 7.

Sequential boosting pair	Affinity drop#	# of AA changes	# of AA closer to WT	comment
11B/10 ➔ 7	Small (5/3)	6/3	4/3	Shown in Figure 7.
11B/10 ➔ 6	Medium (32/16)	7/4	5/4	Shown in Figure 7.
11B/10 ➔ 5	Medium (30/15)	7/5	6/5	Shown in Figure 7.
11B/10 ➔ 3	Large (2700/1300)	9/7	8/7	Shown in Figure 7.
11B/10 ➔ WT	Large (47000/23000)	10/9	10/9	Shown in Figure 7.
7 ➔ 5	Small (6)	2	2	Shown in Figure 7.
7 ➔ 3	Medium (530)	4	4	Shown in Figure 7.
7 ➔ WT	Large (9300)	6	6	Shown in Figure 7.
6 ➔ WT	Large (1500)	5	5	Shown in Figure 7.
5 ➔ WT	Large (1600)	4	4	Shown in Figure 7.
3 ➔ WT	Medium (18)	2	2	Shown in Figure 7.
11B/10 ➔ 2	Large (8200/4100)	10/8	9/8	Would be followed by: 2 ➔ WT
10 ➔ 9A	Small (4)*	1	1	Small affinity drop and only 1 mutation, thus inefficient
7 ➔ 6	Small (6)	1	1	Small affinity drop and only 1 mutation, thus inefficient
6 ➔ 5	Small (1)	1	1	Small affinity drop and only 1 mutation, thus inefficient
6 ➔ 2	Medium (260)	4	4	Would be followed by: 2 ➔ WT
3 ➔ 2	Small (3)	1	1	Small affinity drop and only 1 mutation, thus inefficient
2 ➔ WT	Small (6)	1	1	Small affinity drop and only 1 mutation, thus inefficient

#, Affinity drops were calculated based on binding to the GL+9 antibody, as described in the text, except where noted otherwise. Affinity drops were defined as small (<10), medium (10-1000), or large (>1000).
*, Affinity drops were calculated based on binding to the $GL_{CDR3\text{-}mat}$ antibody.
The immunogen names have "MUT" removed for simplicity.
WT, BG505-T332N.

Supplemental Experimental Procedures

DNA gene synthesis. Genes were synthesized at Genscript, Inc. Gp120 and gp140 variants in pHLsec contained a C-terminal GTKHHHHHH tag. Genes in pENTR contained a C-terminal cMyc epitope followed by a PDGFR transmembrane domain. IgGs were cloned into pFUSEss and Fabs were in a modified version of pFUSEss (pFABss). DNA was maxi-prepped using a BenchPro 2100.

Protein production. BG505-gp120 and variants based on BG505 contained the L111A mutation for more efficient production of monomer compared to other species (Hoffenberg et al., 2013) and the T332N mutation and were expressed in 293F cells grown in 293 Freestyle media (Life Technologies) by transient transfection with 293Fectin (Invitrogen). Protein was harvested from the supernatant 96 h post transfection and purified by nickel affinity chromatography on a HIS-TRAP column (GE) followed by HiLoad 16/600 Superdex 200 size exclusion chromatography (GE Healthcare). Gp140 SOSIPs were expressed in 293F cells grown in 293 Freestyle media by transient transfection with either 293Fectin or PEI. The protein was purified from the supernatant using a HIS-TRAP column, starting with a wash buffer (20 mM Imidazole, 500 mM NaCl, 20 mM Na_2HPO_4) and mixing with elution buffer (500 mM Imidazole, 500 mM NaCl, 20 mM Na_2HPO_4) using a linear gradient. The trimer fraction was collected and further purified on an S200Increase 10-300 column (GE) in HBS (10 mM HEPES, 150 mM NaCl). The oligomeric state of the SOSIP trimers were then confirmed by size exclusion chromatography-multi-angle light scattering (SECMALS) using the DAWN HELEOS II multi-angle light scattering system with Optilab T-rEX refractometer (Wyatt Technology). The trimers were frozen in thin-walled PCR tubes at 1 mg/ml using liquid nitrogen and stored at -80°C (Jardine et al., 2015). Fabs and mAbs were produced in 293F cells as described previously (Jardine et al., 2013). For crystallography, SOSIP_MD39_10MUTA was expressed in 293S cells.

ELISA quanitification of SOSIP expression. BG505 SOSIP variants were expressed using the Freestyle 293F expression system (Thermo Scientific) according to manufacturer's instructions. After 4 days, supernatants were harvested by centrifugation and stored at 4˚C until analysis. Capture ELISAs were performed essentially as described previously (Schiffner et al., 2016). Briefly, ELISA plates were coated overnight with trimer specific PGT145 Fab at 4 μg/mL in PBS at 4˚C followed by blocking with 2% w/v bovine serum albumin (BSA) in washing buffer (PBS + 0.05% v/v tween20). SOSIP expression supernatants were diluted 100x in sample buffer (washing buffer + 1% w/v BSA) and for each variant, a standard curve with known concentration of matching purified protein was prepared in sample buffer. Supernatants and standard curves were added to ELISA plates and detected with trimer preferring IgG PGT151 at 10 μg/mL in sample buffer. Samples were labeled with horseradish peroxidase coupled Fcg-specific anti-human IgG (Jackson Immunoresearch), developed and stopped with 1-Step Ultra TMB-ELISA substrate (Thermo Scientific) as per manufacturer's instructions, and optical densities were read at 450 nm and 570 nm. After background subtraction, data were fit to a "one-site specific binding with hill slope" curve in graphpad prism, and supernatant concentrations were extrapolated from standard curves.

Surface plasmon resonance (SPR). Kinetics and affinities of antibody-antigen interactions were measured on a ProteOn XPR36 (Bio-Rad) using GLC Sensor Chip (Bio-Rad) and 1x HBS-EP+ pH 7.4 running buffer (20x stock from Teknova, Cat. No H8022) supplemented with BSA at 1mg/ml. Human Antibody Capture Kit was used according to manufacturer's instructions (Cat. No BR-1008-39 from GE) to immobilize about 6000 RUs of capture mAb onto each flow cell. In a typical experiment, approximately 300-400 RUs of mAbs were captured onto each flow cell and analytes were passed over the flow cell at 50 μL/min for 3 min followed by a 5 min dissociation time. Regeneration was accomplished using 3M Magnesium Chloride with 180 seconds contact time and injected four times per cycle. Raw sensograms were analyzed using ProteOn Manager software (Bio-Rad), including interspot and column double referencing, and either Equilibrium fits or Kinetic fits with Langmuir model, or both, were employed when applicable. Analyte concentrations were measured on a NanoDrop 2000c Spectrophotometer using Absorption signal at 280 nm (Jardine et al., 2015). We measured kinetics and affinity of antibody-Fab-fragment antigen interactions on ProteOn XPR36 (Bio-Rad) using HTE Sensor Chip (Bio-Rad) and running buffer with 20 mM Sodium Phosphate Dibasic, pH 7.4, 500 mM Sodium Chloride, 50 mM Imidazole, supplemented with BSA at 1mg/ml and Tween 20 detergent at 0.05% v/v. We used 0.1 M Nickel sulfate as activation solution. 0.5 M EDTA was our regeneration solution with 300 seconds contact time and injected two times per cycle (one

time each for vertical and horizontal orientation). Raw sensograms were analyzed using ProteOn Manager software (Bio-Rad), interspot and column double referencing, Equilibrium or Kinetic with Langmuir model or both where applicable. Analyte concentrations were measured on NanoDrop 2000c Spectrophotometer using Absorption signal at 280 nm.

Design of PGT121 germline-targeting immunogens.
BG505-gp120 T332N fused to the PDGFR transmembrane domain (TM) was subjected to random mutagenesis using error prone PCR (gene morph II Agilent), and the resulting PCR product was gel purified and ligated into a modified version of the gateway cloning entry vector pENTR/D-TOPO (Ota et al., 2012) using the circular polymerase extension cloning (CPEC) method (Quan and Tian, 2014). The ligated vector containing the error prone library was purified using the PCR purification kit (Qiagen) and concentrated. The concentrated library was then transformed into electroMAX DH5a-E competent cells (Invitrogen) and grown overnight at 37°C in a 125 mL culture. The plasmid was purified using the BenchPro® 2100 (Invitrogen) and the gp120 insert was transferred to the lentiviral vector pLenti CMVTRE3G puro Dest (Ota et al., 2012) using the LR Clonase II enzyme mix (Invitrogen). The LR clonase reaction was scaled up ~10-fold to increase library size. The LR clonase product was again purified, concentrated and transformed into electroMAX stbl4 competent cells (Invitrogen) and grown overnight at 30°C in a 125 mL culture. This plasmid DNA was purified and ready for use in transfection. 293T cells cultured in Advanced DMEM (Gibco) supplemented with 5% FCS, GlutaMAX (Gibco), 2-mercaptoethanol (Gibco) and Antibiotic-Antimycotic (Gibco) were co-transfected with the BG505-gp120 error prone PCR library in pLenti CMVTRE3G puro Dest (10.8 μg), psPAX2 (7.0 μg) and pMD2.G (3.8 μg) with fugeneHD in a T75 flask (Salmon and Trono, 2007). The cells were kept at 37°C for two days and then the media containing the virus was collected and spun down at 500g for 5min. 293T cells stably expressing rtTA3G from the pLenti CMV rtTA3G Blast vector (obtained from Dave Nemazee; (Ota et al., 2012)) were transduced at low moi (<0.1) in a T75 or T225 flask in the presence of 10 μg/mL blasticidin. The next day cells were selected with 2 μg/mL puromycin. 293T cells containing the stable library were induced with doxycycline (1μg/mL) and the following day were harvested in FACS buffer (HBSS, 1 mM EDTA, 0.5% BSA). Cells were stained with either the GL+9 or GL+3 Ab for ~30 min, washed with FACS buffer, and then stained with fluorescein isothiocyanate (FITC)-labeled α-cMyc (Immunology Consultants Laboratory) and phycoerythrin (PE)-conjugated α-human IgG (Sigma). Cells were sorted on a BD Influx (BD Biosciences) FACS sorter. Appoximately $2x10^5$ GL+9 positive cells were collected and expanded for ~one week in the presence of puromycin and blasticidin before the next round of enrichment was carried out. There was no enrichment for GL+3 positive cells after several rounds of sorting so only the GL+9 positive cells were sequenced. Once the desired population had been obtained the chromosomal DNA was extracted from the cell culture using the GenElute Mammalian Genomic DNA Miniprep Kit (Sigma). The BG505-gp120 gene was PCR amplified from the genomic DNA and ligated back into the Gateway entry vector using CPEC cloning and transformed into top10 competent cells. Later in the design process Gibson assembly was substituted for CPEC cloning. Colonies were sequenced at Genewiz. The sequences were highly enriched for two clones, one containing the N137 glycan knockout by the mutation T139I and the other containing the N133 glycan knockout by the mutation T135A in addition to the T139I mutation. These constructs were called 2MUT (T332N, T139I) and 3MUT (T332N, T135A, T139I). Measuring the affinities of gp120-2MUT and gp120-3MUT against a panel of partially mutated PGT121 Abs (table S1) indicated that knocking out both glycans gave a larger boost in affinity compared to only the N137 glycan-KO so 3MUT was used for further designs.

In parallel to screening the error prone PCR library, a combinatorial library was created based on the structure of PGT122 in complex with BG505 SOSIP (PDB IDs 4NCO and 3J5M). Because the initial SOSIP structures were low resolution and structures of germline PGT121 showed light chain conformational changes we elected to do a saturation mutagenesis combinatorial library that would roughly cover the length of the V1 loop that could potentially interact with germline PGT121 Abs. The library was generated by PCR amplifying the BG505 SOSIP construct in two partially overlapping fragments. The C-terminal fragment was amplified with a primer containing the degenerate codon NNK at four positions in the V1 loop (V134, N136, I138, and D140) as well as a degenerate base encoding N or D at position 137. The two PCR products were ligated together using a second round of PCR, and this second PCR product was inserted into the pENTR vector as described above. The resulting construct was transferred to the pLenti CMVTRE3G puro Dest vector, and lentivirus was produced. Stable cells were stained with the

GL+3 Ab and α-cMyc, and double positive cells were sorted. This resulted in a binding population that was sequenced and found to be a single unique clone containing the mutations V134Y, N136P, I138L, D140N. This clone was called 5MUT (T332N, V134Y, N136P, I138L, D140N). These mutations were combined with the T139I mutation (6MUT) or the T135A/T139I mutations (7MUT).

Next, a saturation mutagenesis scanning library was created on the gp120-7MUT construct using site directed mutagenesis with the QuikChange kit (Agilent Technologies) with a unique NNK/MNN primer pair for each position that was scanned. 11 positions in the V1 loop (T132 to M142) and 10 positions in the V3 loop (T320 to Q328) were scanned and the resulting 21 reactions were pooled, purified, concentrated, and transformed into electroMAX DH5a-E competent cells and transferred to pLenti CMVTRE3G puro Dest as described above. This library was then stained separately with $GL_{CDR3mat}$, $GL_{CDR3rev4}$, or a Chimeric Ab containing the mature PGT121 heavy chain paired with the $GL_{CDR3rev4}$ light chain ($121_H/GL_{L\text{-}rev4}$), as well as α-cMyc for expression. Double positive cells were sorted and 3 mutations were enriched in the $GL_{CDR3mat}$ sort (N137F, T320F, Q328M) and two mutations were enriched in the $121_H/GL_{L\text{-}rev4}$ sort (N135R, Q328M) whereas a binding population was not obtained in the $GL_{CDR3rev4}$ sort. Combining these mutations with 7MUT resulted in $9MUT_A$ (7MUT + N137F/Q328M), $9MUT_B$ (7MUT + N135R/Q328M), and 10MUT (7MUT + N137F/T320F/Q328M). The $9MUT_B$ protein showed improved binding to $121_H/GL_{L\text{-}rev4}$ but worse binding to all other PGT121-class antibodies tested compared to 7MUT (from which $9MUT_B$ was derived) and so $9MUT_B$ was not selected for further use except as a control for the chimeric antibody (data not shown). Gp120-10MUT showed better binding to $GL_{CDR3mat}$ compared to gp120-$9MUT_A$ and T320F was used in subsequent designs with the exception of our SOSIP-$10MUT_A$ crystal structure, which lacks the T320F mutation.

Having established ~1 μM binding to the $GL_{CDR3mat}$ Ab with 10MUT our goal was to improve the immunogen to tolerate more variation within the H-CDR3. For this we created three more V1 loop combinatorial libraries each containing four NNK codons. The three libraries contained NNK codons at positions (A135/P136/F137/L138), (F137/L138/I139/N140), and (I139/N140/D141/M150). Each library was assembled from two partially overlapping ultramers (Integrated DNA Technologies) and ligated into the gp120-10MUT gene using gibson assembly (New England Biolabs). The three libraries were pooled and screened against $GL_{CDR3rev2}$ and $GL_{CDR3rev4}$ Abs. Sorting against the $GL_{CDR3rev4}$ Ab resulted in enrichment for the D141N mutation ($11MUT_A$) and sorting against the $GL_{CDR3rev2}$ resulted in enrichment for L139 and S140 with the most frequent clone containing the sequence N137/L138/L139/S140. When these mutations were combined with the D141N mutation as well as a T415V mutation, which we had identified as being beneficial for binding to PGT121 on an engineered outer domain construct (data not shown), it resulted in $11MUT_B$.

Development of BG505-SOSIP_MD39.

BG505 SOSIP "rare amino acid" library. The BG505 SOSIP "rare amino acid" library was synthesized at GenScript. It was first sorted against PG16 followed by a sort for a high PGT145/B6 binding ratio. The cells were expanded for 1 week and then sorted for either high PGT145/B6 or high PGT151/4025. After six rounds of sorting the library was sequenced (Genewiz). PGT145, PGT151, and PG16 Fabs contained HA epitope tags and were labeled with α-HA-PE (Miltenyi Biotec). B6 and 4025 Fabs contained V5 epitope tags and were labeled with α-V5-FITC (GeneTex).

BG505 SOSIP whole gene saturation mutagenesis. The whole gene saturation mutagenesis library was synthesized at Integrated DNA Technologies in four segments that each contained ~150 NNK codons that were cloned into the BG505-SOSIP gene using either CPEC or Gibson assembly which resulted in four libraries. NNK codons were barcoded with a silent mutation on each side. The libraries created from the second and third segments were combined into one. The first, second and third libraries had NNK codons covering residues Y39-N185, N186-R500 and K502-Q658, respectively. The library that covered gp41 (502-658) was sorted for high PGT145/cMyc, high PGT145/B6, and high PGT151/cMyc. The first gp120 library (39-185) was sorted for high PGT145/B6, and high PGT151/4025. The second gp120 library (186-500) was sorted for high PGT145/cMyc, high PGT145/B6, high PGT151/4025, and high PGT151/cMyc. The sorted libraries were sequenced and analyzed essentially as described previously (Jardine et al., 2016). Positions that enriched for the same amino acid against multiple different mAb sorts (E.g. PGT145(+)/B6(-) and PGT151(+)/4025(-)) were favored for testing in follow up combinatorial libraries or directly testing in recombinantly purified protein. Combinatorial libraries based on the next generation sequencing analysis were assembled from overlapping ultramers and sorted against the same antibodies described above.

Trimer-conjugated liposome synthesis and characterization.

Materials. Lipids 1,2-distearoyl-*sn*-glycero-3-phosphocholine (DSPC) and 1,2-dioleoyl-*sn*-glycero-3-[(N-(5-amino-1-carboxypentyl)iminodiacetic acid)succinyl] (nickel salt) (DGS-NTA(Ni)) were purchased from Avanti Polar Lipids (Alabaster, AL). Cholesterol was purchased from Sigma-Aldrich (St. Louis, MO).

Liposome synthesis. Lipids in chloroform at a 66.5:28.5:5 molar ratio of DSPC:cholesterol:DGS-NTA(Ni) were dried under nitrogen followed by incubation under vacuum for 18 hr at 25 °C. Lipid films were rehydrated with pH 7.4 PBS to a final concentration of 6.5 mM lipid and vortexed 30 s every 10 min for 1 hr at 50 °C. The resulting vesicles were passed through six freeze–thaw cycles between liquid nitrogen and a 50 °C water bath followed by extrusion 21 times through 0.1 µm pore polycarbonate membranes (Whatman Inc, Sanford, ME). Post-liposome formation, 6xHis tagged gp140 trimer was mixed with liposomes at a molar ratio of 42:1 exposed Ni-NTA:trimer (50% of total lipids were assumed to be exposed on the bilayer) and incubated for 2-4 hr at 4 °C. Unconjugated gp140 trimer was then purified away from conjugated liposomes via size exclusion chromatography using a Sepharose CL-2B resin (Sigma) or airfuge (Beckman-Coulter).

Liposome characterization. Total conjugated trimer was quantified by ELISA. Liposomes were treated with 1% triton-X and 100 mM imidazole in PBS containing 1% BSA to destabilize liposomes and Ni-6xHis tag interactions, respectively. Trimer from destabilized liposomes was captured on Nunc MaxiSorp plates with VRCO1 and detected by a mouse anti-6xHis IgG-HRP conjugate (R&D Systems. Minneapolis, MN). Trimer standards were run in parallel and used to calculate final trimer concentrations in each liposome preparation. For the calculation of the number of trimers per liposome, the total lipid concentration in the final liposome preparation was determined using a phospholipid quantification assay (Sigma). This was used to further calculate a theoretical number of monodisperse, unilamellar liposomes using the following equation for the number of lipids (N_{Total}) per liposome, where a = surface area of a single phospholipid head group (0.71nm), h = bilayer width (5nm), and d = liposome diameter:

$$N_{\text{Total}} = \frac{4\pi\left[(d/2)^2\right] + 4\pi\left[(d/2) - h\right]^2}{a}$$

Trimer-conjugated liposomes were also characterized by cryoelectron microscopy (Jeol 2100F TEM) and dynamic light scattering (Wyatt Dyna Pro Plate Reader II) in the Swanson Biotechnology Center at the Koch Institute, MIT. To evaluate trimer antigenicity profiles post-liposome conjugation, intact liposomes were captured on Nunc MaxiSorp plates with VRCO1 (mouse Fc) in PBS containing 1% BSA and detected with various bNAbs or non-NAbs, followed by secondary detection with a goat anti-human IgG-HRP conjugate (Abcam, Cambridge, MA).

Ca^{2+}-flux measurements. Single cell suspensions of spleen and lymph nodes were prepared from mice expressing the predicted germline ($GL_{CDR3rev4}$) or mature heavy and light chain sequences of PGT121. B cells were enriched following the manufacturers instructions by negative selection using anti-CD43 microbeads (Miltenyi Biotec) and magnetized LS columns (Miltenyi Biotec). Enriched cells were washed once in PBS and then diluted in PBS to a concentration of 20 x 10^6 cells/ml. Fluo-4, AM, permeant (Thermo Fisher) and CellTrace Violet (Thermo Fisher) were added to cells to a final concentration of 0.5 µM. Cells were incubated in the dark at 37 °C. After 20 min, labeling was inactivated by the addition of complete medium (RPMI 1640 medium containing 10 mM HEPES and 6% serum) and incubated for 5 min at RT in the dark. Cells were centrifuged and resuspended in complete medium and incubated for an additional 20 min at 37 °C. Cells were centrifuged and resuspended in complete medium without phenol at a concentration of 20 x 10^6 cells/ml and 100 µl aliquots (2 x 10^6 cells) were prepared in FACS tubes. Ca^{2+} flux was detected by flow cytometry (BD LSRFortessa) as increases in fluorescence by Fluo-4 upon binding Ca^{2+} after the addition of stimuli, which was added in a volume of 100 µl to the cells. Ionomycin (final concentration of 1 µg/ml, Sigma) and biotinylated anti-mouse IgM (final concentration of 20 µg/ml, Jackson ImmunoResearch) with the subsequent addition of streptavidine (final concentration of 40 µg/ml, Jackson ImmunoResearch) was added as positive controls. Complete media and the irrelevant antigen

Ovalbumin (final concentration of 300 μg/ml, Sigma) was added as negative controls. Liposomes were added to cells to a final concentration of 300-, 30- and 3 μg/ml and trimers were added to cells to a final concentration of 200-, 20- and 2 μg/ml. Stimuli was added after 30 sec of acquiring un-stimulated cells on the flow cytometer. Ca^{2+}-flux data is presented as the ratio of MFI for Fluo4 and CTV.

Negative-stain electron microscopy. Purified SOSIP trimers, at concentrations between 1.0-1.5 mg/mL as determined by UV A_{280} and an extinction coefficient of $A_{280}^{0.1\%}$ 1.55, were thawed, diluted 1:100 in Tris-buffered saline, and stored on ice until negative-stain EM analysis using a protocol adapted from (de Taeye et al., 2015). Briefly, samples were applied to carbon-coated grids for 10 s, blotted with filter paper, and stained with 2% (w/v) uranyl for 45-60 s prior to blotting with clean filter paper. Data collection was performed using the FEI Tecnai T12 electron microscope and Tietz TemCam-F416 CMOS camera settings described in de Taeye et al. Between 5,000-15,000 single particles were analyzed using reference-free two-dimensional classification (Iterative MSA/MRA method; (Ogura et al., 2003)) and those particles resembling trimers were further classified as having a closed/compact appearance similar to BG505 SOSIP.664, open/breathing phenotype similar to B41 SOSIP.664, or non-native features characteristic of malformed or uncleaved trimers with weakly associated protomers (Pugach et al., 2015; Ringe et al., 2013). Reported native-like percentage is the sum of closed and breathing trimers as a fraction of all trimer particles.

Differential scanning calorimetry (DSC). DSC experiments were performed on a MicroCal VP-Capillary differential scanning calorimeter (Malvern Instruments). The HEPES buffered saline (HBS) buffer was used for baseline scans and the protein samples were diluted into HBS buffer to adjust to 0.25 mg/ml. The system was allowed to equilibrate at 20 °C for 15 min and then heat up till 90°C at a scan rate of 90°C/h. Buffer correction, normalization, and baseline subtraction were applied during data analysis using Origin 7.0 software. The non-two-state model was used for data fitting.

Protein complex formation. Fabs PGT124 and 35022 were produced by transient transfection of 293 FreeStyle™ cells (Invitrogen), and purified by affinity chromatography on a CaptureSelect LC-lambda column (ThermoFisher Scientific), followed by size exclusion chromatography with a S200, 16/60 column (GE Healthcare). SOSIP_MD39_10MUTA was mixed with a 20% molar excess of Fabs PGT124 and 35022, incubated on ice for 15 minutes and then deglycosylated with EndoH (NEB) at 37° in 200mM sodium chloride, 50mM sodium citrate, pH 5.5, for 35 minutes. The ternary complex was then purified by size exclusion chromatography as for the Fabs. The final sample was concentrated to 10.3 mg/mL.

Crystallization and data collection. The crystal used for data collection was obtained at 4° C in a sitting drop tray with precipitant of 5% Peg6000, 0.1M citric acid, pH 4.1. The crystal was briefly immersed in the well solution augmented with 30% Peg200 and flash-cooled in liquid nitrogen. Data were collected at the Advanced Photon Source, beamline 23-ID-D, and processed with HKL-2000 (Otwinowski and Minor, 1997) resulting in data to 4.5Å resolution with 98.4% completeness.

Structure solution and refinement. The structure was determined by molecular replacement with Phaser (McCoy et al., 2007) using model PDB 5CEZ (Garces et al., 2015) with model building carried out using Coot (Emsley et al., 2010). Initial rounds of refinement were carried out with Phenix (Adams et al., 2010), using the 5CEZ coordinates as reference model restraints, with group B factors and TLS refinement, while final rounds of refinement were carried out with Refmac5 (Murshudov et al., 2011), with reference model restraints, jelly body restraints, and TLS refinement. Statistics for data collection and final refinement are listed in Table S1.

ELISA to characterize antigenic profile of native-like trimers. 96-well plates were coated overnight at 4°C with 6x-His Epitope Tag Antibody (Thermofisher) at 2 mg/ml in PBS. Plates were washed 3 times with PBS, 0.05% Tween (PBS-T), and blocked with 10% milk PBS for 1h. Subsequently, 2 mg/ml of the purified His-tagged SOSIP protein was added for 2 h in 1% milk PBS-T, after which the plates were washed three times with PBS-T. Serial dilutions of mAbs in 1% milk PBS-T were added to the plates for 1 h, after which the plates were washed again three times with PBS-T before the addition of anti-human Fc region -conjugated alkaline phosphatase (Jackson Immunoresearch) at 1:1000 for 1 h. After four final

washes, binding was detected by the addition of alkaline phosphatase substrate and measured by absorbance at 405 nm.

Development of variable loop cocktail (VLC) trimers. Using BG505 SOSIP MD39 trimer as a base, a series of new trimers were engineered by replacing the immunodominant variable loops of the BG505 strain with loops from alternative strains. Given the vast number of HIV strains available, we created three separate criteria to guide our loop selection. For the first set of variable loop transplants, we cataloged the number of glycans within each variable loop and the length of each variable loop (Figure 7C). Certain combinations of variable loop lengths and glycans were observed more frequently than others across HIV strains (e.g. 20.48 % of HIV strains have a 14 amino acid variable loop 2 with one glycan, Figure 7C). We searched for strains that contain the most common loop length/glycan combination for each of the variable loops (V1,V2,V4,V5). For the second set of variable loop transplants, we searched for strains with variable loops of the same length and number of glycans as BG505, but with very different amino acid sequence and glycan positioning within the loops as compared to BG505. No single strain had all the same variable loop lengths and number of glycans as BG505, so we relaxed our criteria and matched each variable loop independently for this set of variable loop transplants only. For the third set of variable loop transplants, we searched for strains with exceptionally long variable loops (V1,V2,V4 must be ≥ 4 amino acids longer than BG505). Under each of these criteria, we were able to obtain one or two trimers that had a reasonable level of expression and formed well-behaved native-like trimers (Figure S7A). The loops of the VLCs are defined as: VLC-1 (V1: BES10.EF363127, V2: BL8157. DQ886035, V4: BF1P51.JQ250880, V5: CZM197.DQ388515), VLC-2(BG505. DQ208458), VLC-3 (PRLS08.FJ469757) VLC-4(GHJ193.AB231897), VLC-5(OUR2478P.EF165541). A region defined as 335-351 (HxB2) underneath V4 was included when transplanting V4, due to high variability and close contact with V4. Including BG505, we report a set of 5 trimers with diverse variable loops. One version of the VLC trimers that did not have the MD39 mutations, and instead contained an extra stabilizing disulfide (DS21: V120C-Q315C) in order to staple down the tip of the V3, this version of the VLC trimers was used in an accompanying manuscript (Escolano et al., 2016).

Structural alignment of trimers. The alignment of native-like trimers was done using the alignMolecules program from MSL (Kulp et al., 2012) and verified using PyMOL. The following residues from all three subunits were used to align 5CEZ, 4TVP and the MD39 structure reported here: 32-60+65-132+153-184+189-396+411-505+518-546+572-664.

Supplemental References

Adams, P.D., Afonine, P.V., Bunkoczi, G., Chen, V.B., Davis, I.W., Echols, N., Headd, J.J., Hung, L.W., Kapral, G.J., Grosse-Kunstleve, R.W., *et al.* (2010). PHENIX: a comprehensive Python-based system for macromolecular structure solution. Acta Crystallogr D *66*, 213-221.

Hoffenberg, S., Powell, R., Carpov, A., Wagner, D., Wilson, A., Kosakovsky Pond, S., Lindsay, R., Arendt, H., Destefano, J., Phogat, S., *et al.* (2013). Identification of an HIV-1 clade A envelope that exhibits broad antigenicity and neutralization sensitivity and elicits antibodies targeting three distinct epitopes. J Virol *87*, 5372-5383.

Kulp, D.W., Subramaniam, S., Donald, J.E., Hannigan, B.T., Mueller, B.K., Grigoryan, G., and Senes, A. (2012). Structural informatics, modeling, and design with an open-source Molecular Software Library (MSL). Journal of computational chemistry *33*, 1645-1661.

McCoy, A.J., Grosse-Kunstleve, R.W., Adams, P.D., Winn, M.D., Storoni, L.C., and Read, R.J. (2007). Phaser crystallographic software. J Appl Crystallogr *40*, 658-674.

Murshudov, G.N., Skubak, P., Lebedev, A.A., Pannu, N.S., Steiner, R.A., Nicholls, R.A., Winn, M.D., Long, F., and Vagin, A.A. (2011). REFMAC5 for the refinement of macromolecular crystal structures. Acta Crystallogr D *67*, 355-367.

Ogura, T., Iwasaki, K., and Sato, C. (2003). Topology representing network enables highly accurate classification of protein images taken by cryo electron-microscope without masking. Journal of structural biology *143*, 185-200.

Otwinowski, Z., and Minor, W. (1997). Processing of X-ray diffraction data collected in oscillation mode. Method Enzymol *276*, 307-326.

Quan, J., and Tian, J. (2014). Circular polymerase extension cloning. Methods in molecular biology *1116*, 103-117.

Ringe, R.P., Sanders, R.W., Yasmeen, A., Kim, H.J., Lee, J.H., Cupo, A., Korzun, J., Derking, R., van Montfort, T., Julien, J.P.*, et al.* (2013). Cleavage strongly influences whether soluble HIV-1 envelope glycoprotein trimers adopt a native-like conformation. Proceedings of the National Academy of Sciences of the United States of America *110*, 18256-18261.

Salmon, P., and Trono, D. (2007). Production and titration of lentiviral vectors. Current protocols in human genetics / editorial board, Jonathan L Haines [et al] *Chapter 12*, Unit 12 10.

Schiffner, T., de Val, N., Russell, R.A., de Taeye, S.W., de la Pena, A.T., Ozorowski, G., Kim, H.J., Nieusma, T., Brod, F., Cupo, A.*, et al.* (2016). Chemical Cross-Linking Stabilizes Native-Like HIV-1 Envelope Glycoprotein Trimer Antigens. Journal of virology *90*, 813-828.
